# Uncommon Polyketides from *Penicillium steckii* AS-324, a Marine Endozoic Fungus Isolated from Deep-Sea Coral in the Magellan Seamount

**DOI:** 10.3390/ijms23116332

**Published:** 2022-06-06

**Authors:** Xue-Yi Hu, Xiao-Ming Li, Bin-Gui Wang, Ling-Hong Meng

**Affiliations:** 1 CAS and Shandong Province Key Laboratory of Experimental Marine Biology, Institute of Oceanology, Chinese Academy of Sciences, Nanhai Road 7, Qingdao 266071, China; xueyihu61@163.com (X.-Y.H.); lixmqd@qdio.ac.cn (X.-M.L.); 2 Laboratory of Marine Biology and Biotechnology, Qingdao National Laboratory for Marine Science and Technology, Wenhai Road 1, Qingdao 266237, China; 3 College of Marine Science, University of Chinese Academy of Sciences, Yuquan Road 19A, Beijing 100049, China; 4 Center for Ocean Mega-Science, Chinese Academy of Sciences, Nanhai Road 7, Qingdao 266071, China

**Keywords:** marine endozoic fungus, *Penicillium steckii*, polyketide derivatives, natural products, structure elucidation, biological activity

## Abstract

Four unusual steckwaic acids E–H (**1**–**4**), possessing a rarely described acrylic acid unit at C-4 (**1**–**3**) or a double bond between C-12 and C-13 (**4**) are reported for the first time, along with four new analogues (**5**–**8**) and two known congeners (**9** and **10**). They were purified from the organic extract of *Penicillium steckii* AS-324, an endozoic fungus obtained from a deep-sea coral *Acanthogorgiidae* sp., which was collected from the Magellan Seamount at a depth of 1458 m. Their structures were determined by the interpretation of NMR and mass spectroscopic data. The relative and absolute configurations were determined by NOESY correlations, X-ray crystallographic analysis, and ECD calculations. All compounds were tested for their antimicrobial activities against human- and aquatic-pathogenic bacteria and plant-related pathogenic fungi.

## 1. Introduction

Deep-sea-living organisms have evolved under extreme environmental conditions, which have influenced the development of various biochemical functions compared to those living in shallow-water organisms [1,2]. Although the area of deep-sea habitats is much larger than that of shallow-sea habitats, compounds isolated from deep-sea organisms accounted for only ~2% of the more than 30,000 marine natural products [3,4], whereas approximately 75% of these molecules exhibited notable bioactivities. Improved technological capacity for sampling from the deep-sea environment has improved the discovery of deep-sea-derived natural products. In recent years, the number of deep-sea-sourced natural products increased rapidly, and these compounds usually display high bioactivity hits in bioassays [5]. Although the nature of the associations between a host and its associated microbes is far from understood, there is growing evidence that some coral-associated fungi have adopted the ability to produce secondary metabolites that are structurally divergent from their terrestrial counterparts [6,7,8]. Marine animal-related fungi often produce bioactive metabolites that might be interpreted as chemically mediated defense mechanisms to protect their host organisms from environmental hazards such as predation and pathogenic invasion [9]. Some studies have reported on the isolation of marine coral-associated *Penicillium* spp. as producers of bioactive metabolites [10,11,12].

**Figure 1:**
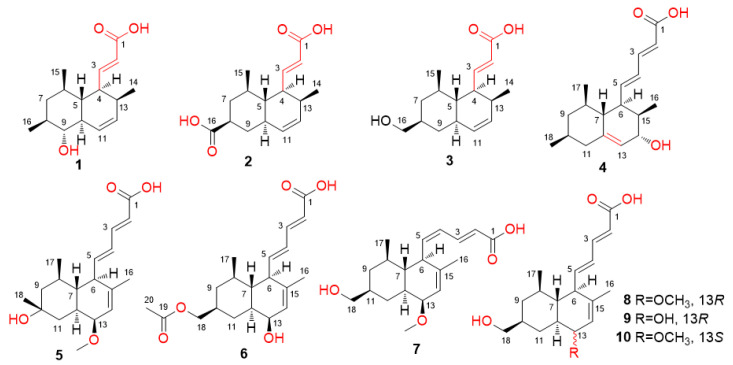
Structures of compounds **1**–**10**.

Following our ongoing research about secondary metabolites from marine-derived fungi collected from deep-sea habitats [13,14], an endozoic fungus *Penicillium steckii* AS-324, obtained from the fresh tissues of deep-sea coral *Acanthogorgiidae* sp. collected from Magellan seamount in the Western Pacific Ocean at a depth of 1458 m, was cultured and chemically investigated. *P. steckii* has recently been proved to be an excellent source of antibacterial compounds based on genome sequencing and mining, as well as antibacterial screening of the crude extracts [15]. Eight new tanzawaic acid derivatives (**1**–**8**), together with two known analogues, tanzawaic acid H (**9**) [16] and tanzawaic acid S (**10**) [17], were isolated and identified. We recently reported 10 new tanzawaic acid derivatives, including steckwaic acids A–D from *P. steckii* AS-324 [18]. Further work on the remaining portions of this fungus resulted in the characterization of eight new (**1**–**8**) and two known (**9** and **10**) tanzawaic acid analogues (Figure 1). Among these compounds, steckwaic acids E–G (**1**–**3**) possess a rarely described acrylic acid unit at C-4, while compound **4** has a double bond at C-12, which is uncommon among the reported tanzawaic acids [16,17,18]. Details of the isolation, structure elucidation, and antimicrobial activity of compounds **1**–**10** are described herein.

## 2. Results

The culture of *P. steckii* AS-324 was extracted with EtOAc to gain the organic extract, which was then fractionated and purified by various chromatographic methods to yield compounds **1**–**10** (Figure 1).

**Figure 2:**
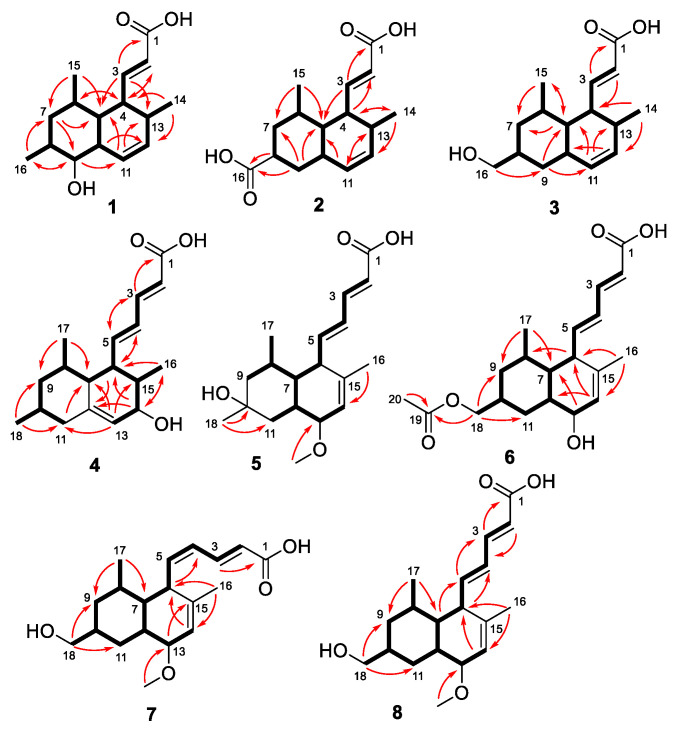
Key COSY (bold lines) and HMBC (arrows) correlations of compounds **1**–**8**.

**Figure 3:**
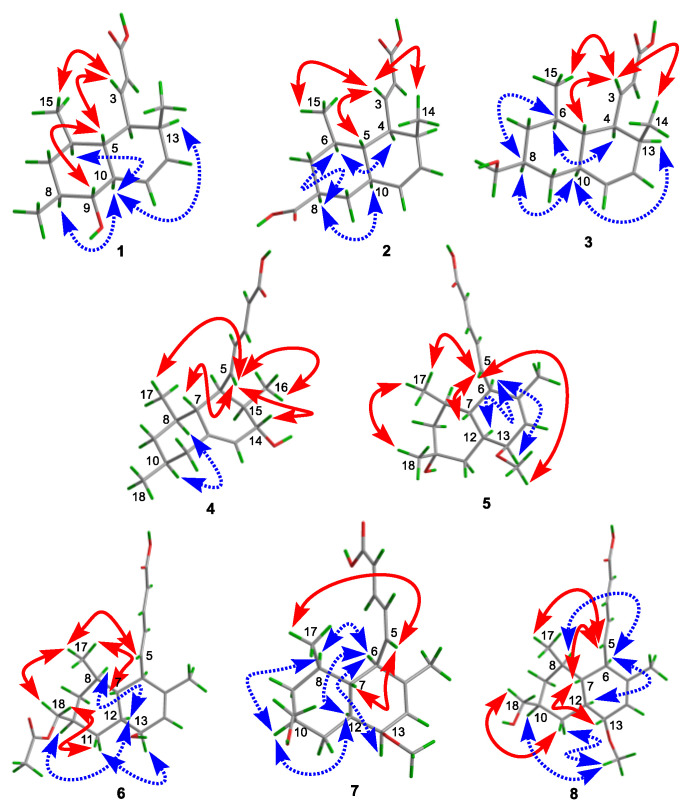
Key NOESY correlations of compounds **1**–**8**.

Steckwaic acid E (**1**) was initially obtained as a white amorphous powder, and the molecular formula was assigned as C_16_H_24_O_3_ by negative HRESIMS data. The ^1^H and ^13^C NMR data (Table 1) revealed the presence of three doublet methyl substitutions, one methylene, eleven methines (with one oxygenated and four olefinic), and one carboxyl carbon. A large spin system incorporating H-2 through H-13 and three methyls, H_3_-14, H_3_-15, and H_3_-16, confirmed the presence of a decalin skeleton with three methyl-substitution at C-6, C-8, and C-13 (Figure 2). HMBC correlations from H-3 to C-1, C-5, and C-13 and from H-2 to C-4 indicated the position of one acrylic acid side chain at C-4 (Figure 2). The large coupling constant between H-2 and H-3 (*J* = 15.4 Hz) suggested the *E*-geometry of the double bond, whereas the smaller coupling constant between H-11 and H-12 (*J* = 9.8 Hz) revealed the *Z*-geometry. Thus, the planar structure of **1** was determined. The coupling constants between H-4 and H-5, between H-5 and H-10, and between H-8 and H-9, as well as between H-9 and H-10, were all 9.5 Hz, indicating the *trans*-orientation of these adjacent proton pairs in the cyclohexane/cyclohexene units. NOESY correlations from H-3 to H-5 and H_3_-15, as well as from H-5 to H-9, demonstrated them on the same face of these protons, while correlations from H-10 to H-6, H-8, and H-13 placed them on the opposite side (Figure 3).

**Figure 4:**
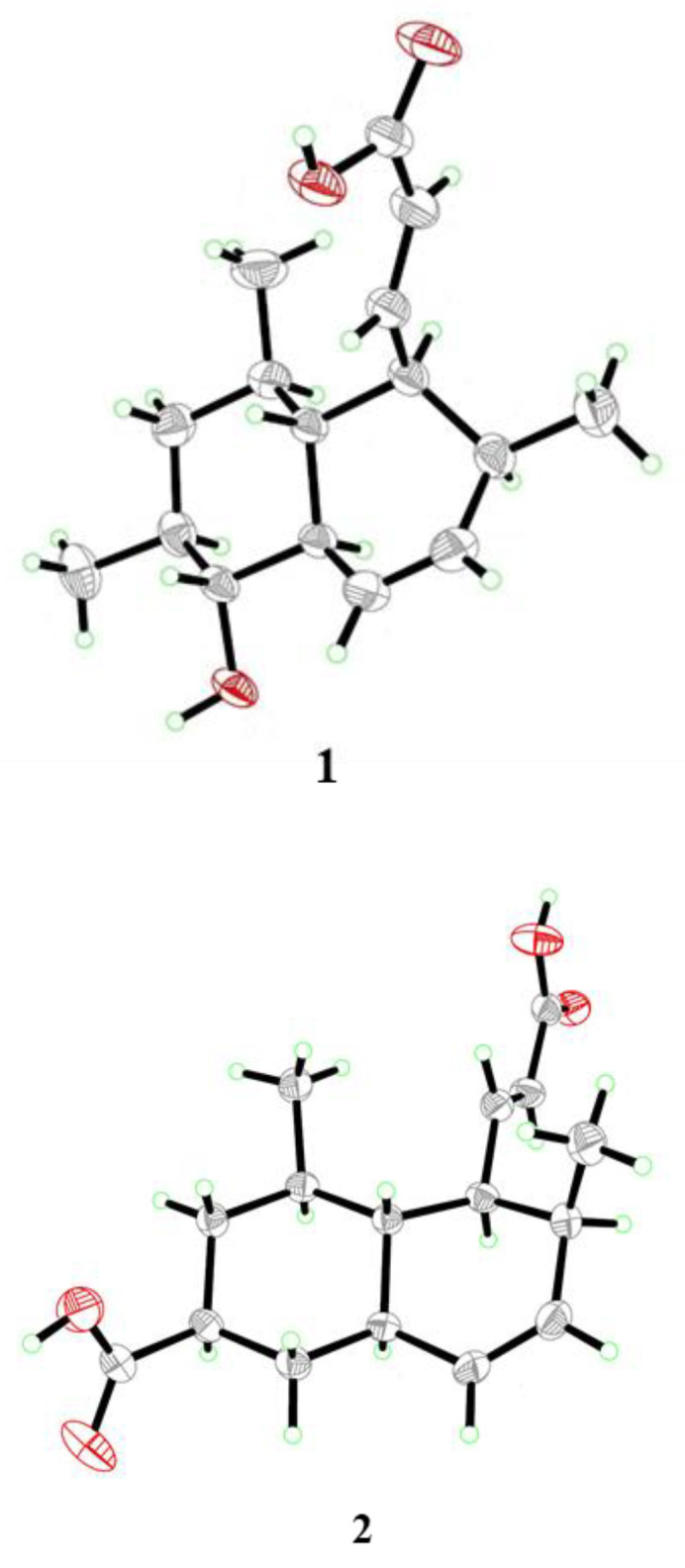
X-ray crystallographic structures of compounds **1** and **2** (with a thermal ellipsoid probability of 50%).

To unambiguously confirm the structure and configuration of compound **1**, attempts to cultivate quality crystals were performed, and suitable crystals were obtained by dissolving the samples in MeOH and refrigerating them to evaporate the solvent slowly. Single-crystal X-ray diffraction analysis using Cu K*α* radiation confirmed the structure of **1**, and the absolute configuration was 4*S*, 5*R*, 6*R*, 8*S*, 9*R*, 10*R*, and 13*S* with the Flack parameter −0.09(13) (Figure 4).

Steckwaic acid F (**2**), obtained as colorless crystals, was assigned a molecular formula of C_16_H_22_O_4_ according to the HRESIMS analysis. Detailed inspection of its NMR data revealed the same skeleton as that of compound **1**, and the main differences are that the oxygenated methine resonating at *δ*_C_/*δ*_H_ 77.0/2.59 (CH-9) in **1** was replaced by methylene resonating at *δ*_C_/*δ*_H_ 35.4/1.93 and 1.11 (CH_2_-9) in **2**, whereas a carboxyl carbon resonating at *δ*_C_ 176.9 (C-16) was observed by HMBC in **2**, which replaced the methyl group resonating at *δ*_C_/*δ*_H_ 19.2/0.91 (CH_3_-16) in **1**. The large spin system similar to that in **1** was otherwise intact, albeit with some chemical shift differences and the absence of the methyl group CH_3_-16 in **2** (Figure 2). HMBC correlations from H-8 and H-9 to the carboxyl carbon C-16, as well as from H-9 to C-5 and C-7, confirmed the planar structure of compound **2** (Figure 2). The coupling constant between H-2 and H-3 (*J* = 15.3 Hz) suggested the *E*-geometry of the double bond. NOE correlations from H-3 to H-5, H_3_-14, and H_3_-15 suggested the protons were in the same orientation, whereas correlations from H-6 to H-4 and H-8 as well as from H-8 to H-10 positioned them on the other side (Figure 3). The structure and absolute configuration of compound **2** were confirmed by X-ray diffraction analysis using Cu K*α* radiation, and the Flack parameter 0.05(10) permitted assignment of the absolute configuration as 4*S*, 5*S*, 6*R*, 8*S*, 10*R,* and 13*S* (Figure 4).

**Figure 5:**
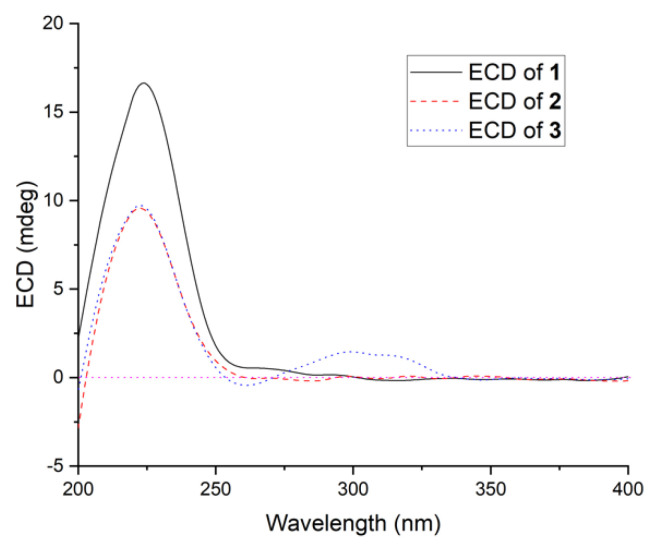
ECD spectra of compounds **1**–**3**.

Steckwaic acid G (**3**), obtained as a white amorphous powder, was assigned the molecular formula C_16_H_24_O_3_ by HRESIMS and NMR data. The chemical structure of compound **3** was almost identical to that of **2**. The main difference between **3** and **2** was evident in the nonappearance of one of the two carboxyl carbons C-16 (*δ*_C_ 176.9 in **2**) in the ^13^C-NMR spectrum of **3**, the appearance of ^1^H- and ^13^C-NMR resonances for an oxymethylene moiety CH_2_-16 (*δ*_C_ 66.3 and *δ*_H_ 3.20 in **3**), and the related changes in chemical shifts and multiplicities of nearby carbons and protons around CH-9 (Table 1). This spectral evidence suggests that the carboxyl carbon C-16 in **2** was reduced to form an oxymethylene unit in **3**, and this assignment was further confirmed by COSY and HMBC correlations (Figure 2). The coupling constant between H-2 and H-3 (*J* = 15.4 Hz) was similar to that observed in compound **2**, which indicated the *E*-geometry of the double bond at C-2. NOESY correlations from H-3 to H-5, H_3_-14, and H_3_-15 supported the cofacial orientation of these protons, while correlations from H-6 to H-4 and H-8, as well as from H-10 to H-8 and H-13, placed these groups on the opposite face (Figure 3). The ECD spectrum of **3** closely matched that of **1** and **2**, which showed the same positive cotton effects near 225 nm (Figure 5), enabling the assignment of the absolute configurations of compound **3** as 4*S*, 5*S*, 6*R*, 8*S*, 10*R*, and 13*S*.

**Figure 6:**
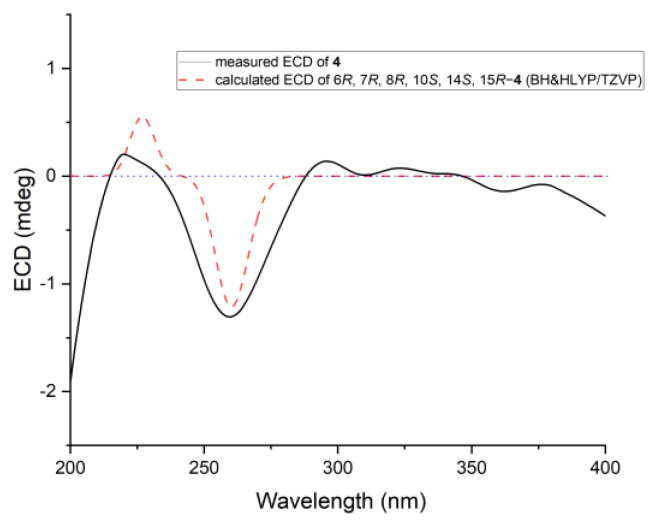
Experimental and calculated ECD of compound **4**.

Steckwaic acid H (**4**) was determined to have the molecular formula C_18_H_26_O_3_ based on the negative HRESIMS data. Its NMR data (Table 2) displayed typical signals of a decalin skeleton with three methyl signals for C-8, C-10, and C-15, and the appropriately modified acrylic acid substituent in **1**–**3** was changed to a penta-2,4-dienoic acid moiety in **4**. HMBC correlations from the olefinic proton H-13 to C-7, C-11, and C-15, as well as from H-6 and H-14 to the nonprotonated olefinic carbon C-12, were located in the position of a double bond between C-12 and C-13. In addition, HMBC correlations from the oxymethine proton H-14 to C-6, C-12, and C-18 confirmed the hydroxy-substituent at C-14. Large coupling constants between H-2 and H-3 (*J* = 15.3 Hz), as well as H-4 and H-5 (*J* = 15.1 Hz), showed the *E*-geometry of two double bonds at C-2 and C-4. The relative configuration of **4** was determined by a NOESY spectrum. NOE correlations from H-5 to H-7, H-14, H_3_-16, and H_3_-17 placed them on the same face of the molecule, whereas correlations from H-8 to H-10 indicated these protons were on the other side. To clarify the absolute configuration of **4**, the ECD spectra of minimum energy conformers by the TDDFT method at BH&HLYP/TZVP and CAM-B3LYP/TZVP levels were calculated, and the experimental ECD spectrum of **4** matched well with that of the calculated spectrum for (6*R*, 7*R*, 8*R*, 10*S*, 14*S*, and 15*R*)-**4** (Figure 6 and Figure S59).

**Figure 7:**
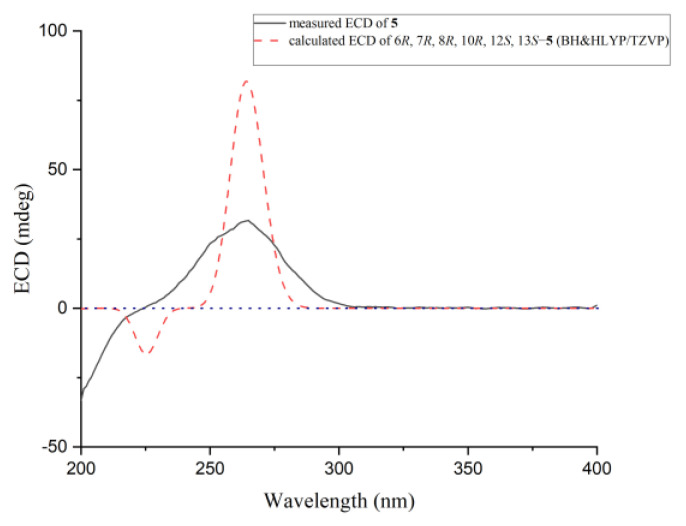
Experimental and calculated ECD of compound **5**.

**Figure 8:**
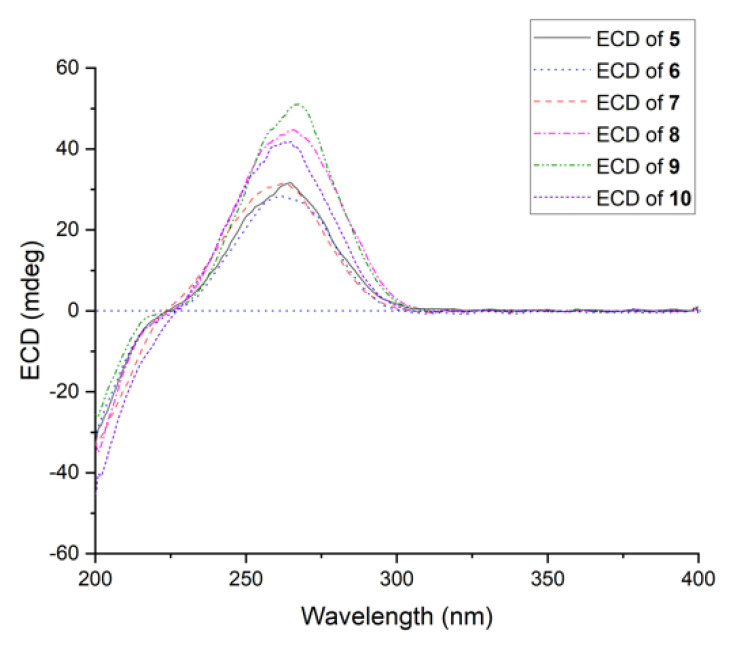
Experimental ECD of compounds **5**–**10**.

Compound **5** was obtained as a white amorphous powder, and the molecular formula was assigned as C_19_H_28_O_4_ by HRESIMS data. Detailed analysis of the ^1^H and ^13^C data (Table 2) showed that it was similar to tanzawaic acid U [17] except that resonances for the methine unit at *δ*_C_ 32.3 and *δ*_H_ 1.46 (CH-10) in tanzawaic acid U were replaced by an oxygenated/nonprotonated carbon at *δ*_C_ 68.0 (C-10) in **5**. These observations were further supported by relevant COSY and HMBC correlations (Figure 2). The coupling constants of two double bonds at C-2 and C-4 were 15.2 and 15.3 Hz, respectively, suggesting *E*-geometry for the double bonds. NOE correlations from H-5 to H-7 and H_3_-17, as well as from H_3_-17 to H_3_-18, indicated the same orientation of these protons, while correlations from H-6 to H-12 and H-13 showed them on the opposite side. The absolute configuration of **5** was determined by both ECD calculation and comparisons with known compounds **9** and **10**. The experimental ECD spectrum of **5** aptly matched the calculated spectra of (6*R*, 7*R*, 8*R*, 10*R*, 12*S*, 13*S*)-**5** at BH&HLYP/TZVP, CAM-B3LYP/TZVP, and PBE0/TZVP levels (Figure 7 and Figure S59). Besides, **5** showed a positive Cotton effect at approximately 264 nm similar to that of compounds **9** and **10** (Figure 8), which also confirmed the absolute configuration of **5**. Compound **5** was named 10-hydroxytanzawaic acid U.

Compound **6** was isolated as a white amorphous powder and assigned the molecular formula C_20_H_28_O_5_ by negative HRESIMS data. A detailed comparison of the NMR spectral data (Table 2) with the known compound tanzawaic acid R [17] suggested that they were very similar. However, signals for a carboxyl carbon at *δ*_C_ 170.4 and a methyl group at *δ*_C_/*δ*_H_ 20.7/2.00 related to an acetoxyl group were observed in the NMR spectra of **6**. HMBC correlations from H_2_-18 to C-9, C-11, and C-19 as well as H_3_-20 to C-19 placed the acetoxyl group at C-18 (Figure 2). Large coupling constants between H-2 and H-3, as well as between H-4 and H-5 (*J* = 15.3 Hz), indicated the *E*-geometry of two double bonds. NOESY correlations from H_3_-17 to H-5, H-7, and H_2_-18, as well as from H_2_-18 to H-11*β*, suggested the same orientation of these protons, while correlations from H-12 to H-8 and H-10 as well as from H-13 to H-11*α* placed these groups on the opposite face. From the ECD data and biogenetic considerations, the absolute configuration of compound **6** was assigned as 6*R*, 7*R*, 8*R*, 10*S*, 12*S*, and 13*S*. Compound **6** was named 18-*O*-acetyltanzawaic acid R.

Steckwaic acid I (**7**) was obtained as a white amorphous powder, and the molecular formula was assigned as C_19_H_28_O_4_ by analysis of the HRESIMS data. A detailed comparison of the NMR spectral data between **7** (Table 3) and tanzawaic acid S (**10**) [17] suggested that they were very similar, except the coupling constant between H-4 and H-5 (*J* = 11.1 Hz) in **7** was much smaller than that of **10** (*J*_4,5_ = 15.3 Hz), indicating the geometry of the double bond at C-4 changed from *E* in **10** to *Z* in **7**. The planar structure was further determined by COSY and HMBC correlations (Figure 2), and the relative configuration was confirmed by the NOESY spectrum. The NOESY correlations from H-5 to H-7 and H_3_-17 showed these groups to be on the same side, whereas correlations from H-6 to H-8, H-12, and H-13, as well as from H-10 to H-8 and H-12, indicated the opposite side of these protons. The absolute configuration of **7** was confirmed by comparing the ECD spectrum with those of known compounds **9** and **10**. The same positive Cotton effects around 265 nm demonstrated consistent absolute configurations with those of 6*R*, 7*R*, 8*R*, 10*S*, 12*S*, and 13*S*.

Compound **8** was also obtained as a white amorphous powder with the molecular formula C_19_H_28_O_4_ as measured by HRESIMS. Analysis of ^1^H and ^13^C NMR data (Table 3) showed similarities to those reported for tanzawaic acid S (**10**) that were measured in methanol-*d*_4_ [17] and our isolates that were measured in DMSO-*d*_6_ (Experimental section). The primary differences between **8** and **10** were the signals of an oxygenated methine at *δ*_C_/*δ*_H_ 80.3/3.32 (CH-13) in **8** replaced by signals at *δ*_C_/*δ*_H_ 75.3/3.37 (CH-13) in **10**, revealing the configuration at C-13 had been changed. This deduction was further supported by the relevant COSY and HMBC correlations shown in Figure 2, as well as NOESY data shown in Figure 3. NOE correlations from H-5 to H-7 and H_3_-17, from H-7 to H-13, and from H_2_-18 to H-11*β* suggested they were on one side, whereas correlations from H-6 to H-8 and H-12 and from H-13-OCH_3_ to H-10 and H-11*α* placed them on another face. By comparing the ECD spectrum with that of compounds **9** and **10**, the absolute configuration of **8** was assigned as 6*R*, 7*R*, 8*R*, 10*S*, 12*S,* and 13*R*. Compound **8** was named 13*R*-tanzawaic acid S.

In addition to the new compounds **1**−**8**, two structurally related known analogues **9** and **10** were also isolated, and their structures were identified as tanzawaic acid H (**9**) and tanzawaic acid S (**10**) based on the comparison of NMR data, optical rotations, and ECD spectra with those described in literature reports [16,17].

The absolute configurations of major chiral centers for compounds **1**−**10** are consistent except for position C-13. In comparison to 13*S* methoxy-substituent (compounds **5**, **7**, and **10**), the resonance of C-13 in the 13*R* isomer (compound **8**) shifted ~5 ppm downfield in the ^13^C NMR spectra. An analogous carbon (4.4 ppm) was observed for the analogous hydroxy-substituent when comparing compound **6** with compound **9**. Besides, for the compounds with the same absolute configuration at C-13 (**5**, **6**, **7,** and **10**), the methoxy-substituent (**5**, **7,** and **10**) could shift downfield approximately 10 ppm in the ^13^C NMR spectra compared to that of hydroxy-substituent (**6**).

Although some tanzawaic acid derivatives were tested for their cytotoxic [16] and lipid-lowering [17] activities, a few of them exhibited significant activities. Compounds **1**–**10** were assayed for their antibacterial activities against one human and nine aquatic pathogenic bacteria as well as seven plant-pathogenic fungi. Compound **8** showed moderate activity against the human pathogenic bacterium *Escherichia coli* with an MIC value of 8 μg/mL (the MIC value of the positive control chloramphenicol was 1 μg/mL), while compound **10** exhibited inhibitory activity against the aquatic pathogenic bacterium *Edwardsiella tarda* with an MIC value of 16 μg/mL (the MIC value of the positive control chloramphenicol was 2 μg/mL) (Table 4). The results suggested that the absolute configuration of C-13 influenced the antibacterial activities of different bacteria (**8** vs. **10**) as supported by the fact that compound **8** with a 13*R* configuration showed stronger activity against *E. coli* but weaker activity against *E. tarda*, while compound **10** with a 10*S* configuration showed stronger activity against *E. tarda* but no activity against *E. coli*. The geometry of the double bond at C-4 also affected the activity (**7** vs. **10**), revealed by the fact that the *E*-geometry (**10**) exhibited stronger activity against *E. tarda* while compound **7** with *Z*-geometry at C-4 did not show any activity. Other compounds did not exhibit antimicrobial activities (MIC > 64 μg/mL).

## 3. Materials and Methods

### 3.1. General Experimental Procedures

General experimental procedures were the same as previously reported [19,20].

### 3.2. Fungal Material

The fungal strain *Penicillium steckii* AS-_324_ was obtained from fresh tissues of *Acanthogorgiidae* sp., which were collected from Magellan seamount. Taxonomic identification of the fungus was accomplished by comparing its ITS region sequence to that of *Penicillium steckii* (MT582790.1), which showed 99.64% similarity. The sequence data of the fungus AS-324 were submitted and deposited in GenBank with the accession no. OK605032. The fungal strain is preserved at the Key Laboratory of Experimental Marine Biology, Institute of Oceanology, Chinese Academy of Sciences (IOCAS, Qingdao, China).

### 3.3. Fermentation, Extraction, and Isolation

For chemical investigations, the fresh mycelia of *P. steckii* AS-324 were grown on PDA medium at 28 °C for five days and were then inoculated into 125 × 1 L Erlenmeyer flasks with rice solid medium (70 g rice, 0.1 g corn syrup, 0.3 g peptone, 0.1 g methionine, and 100 mL naturally sourced and filtered seawater that was obtained from the Huiquan Gulf of the Yellow Sea near the campus of IOCAS), and statically cultured for 30 days at room temperature. After incubation, the fermented rice substrate was extracted three times with EtOAc. The combined EtOAc extracts were filtered and evaporated under reduced pressure to yield organic extract (120 g), which was subjected to vacuum liquid chromatography (VLC) eluting with different solvents of increasing polarity from petroleum ether (PE) to MeOH to yield nine fractions (Frs. 1–9). Fr. 6 (11 g), eluted with PE–EtOAc (1:1), was purified by column chromatography (CC) over Lobar LiChroprep RP-18 with a MeOH-H_2_O gradient (from 10:90 to 100:0) to afford seven subfractions (Frs. 6.1–6.7). Fr. 6.4 (eluted with MeOH-H_2_O, 40:60, 260 mg) was further purified by CC on Si gel eluting with a CH_2_Cl_2_-MeOH gradient (from 100:1 to 20:1) to gain Frs. 6.4.1−6.4.3. Fr. 6.4.1 was further purified by prep. TLC (plate: 20 × 20 cm, developing solvents: PE-Acetone, 1:1) and Sephadex LH-20 (MeOH) to obtain compound **1** (42.3 mg). Fr. 6.4.2 was further purified by semi-preparative HPLC (Elite ODS-BP column, 10 μm; 20 × 250 mm; 50% MeOH-H_2_O, 10 mL/min) to gain compounds **2** (15.6 mg, *t*_R_ = 15.5 min) and **4** (22.8 mg, *t*_R_ = 17.5 min). Fr. 6.4.3 was purified by prep. TLC (plate: 20 × 20 cm, developing solvents: CH_2_Cl_2_-Acetone, 3:1) and Sephadex LH-20 (MeOH) to obtain compound **3** (10.2 mg). Fr. 7 (5.5 g), eluted with CH_2_Cl_2_-MeOH (20:1), was purified by reverse-phase column chromatography (CC) over Lobar LiChroprep RP-18 with a MeOH-H_2_O gradient (from 10:90 to 100:0) to yield five subfractions (Frs. 7.1–7.5). Fr. 7.3 (eluted with MeOH-H_2_O, 50:50, 326 mg) was further purified by CC on Si gel eluting with a CH_2_Cl_2_-MeOH gradient (from 100:1 to 20:1), prep. TLC (plate: 20 × 20 cm, developing solvents: PE-Acetone, 1:1), and Sephadex LH-20 (MeOH) to obtain compounds **5** (3.0 mg) and **10** (38.7 mg). Fr. 7.4 (eluted with MeOH-H_2_O, 60:40, 118 mg) was fractionated by CC on Si gel eluting with a CH_2_Cl_2_-MeOH gradient (from 80:1 to 20:1) and Sephadex LH-20 (MeOH) to afford compound **6** (20.4 mg). Fr. 8 (10.0 g), eluted with CH_2_Cl_2_/MeOH (10:1), was purified by reverse-phase CC over Lobar LiChroprep RP-18 with an MeOH-H_2_O gradient (from 10:90 to 100:0) to yield three subfractions (Frs. 8.1–8.3). Fr. 8.1 (eluted with MeOH–H_2_O 30:70) was further purified by CC on Si gel eluting with a CH_2_Cl_2_-MeOH gradient (from 100:1 to 50:1) and semi-preparative HPLC (Elite ODS-BP column, 10 μm; 20 × 250 mm; 60% MeOH-H_2_O, 10 mL/min) to gain compounds **7** (13.2 mg, *t*_R_ = 18.7 min) and **9** (8.9 mg, *t*_R_ = 16.5 min). Fr. 8.3 (eluted with MeOH-H_2_O 50:50) was further purified by CC on Si gel eluting with a CH_2_Cl_2_-MeOH gradient (from 100:1 to 50:1), prep. TLC (plate: 20 × 20 cm, developing solvents: PE-Acetone, 1:1), semi-preparative HPLC (Elite ODS-BP column, 10 μm; 20 × 250 mm; 45% MeOH-H_2_O, 10 mL/min), and Sephadex LH-20 (MeOH) to obtain compound **8** (3.0 mg).

*Steckwaic acid E(***1***):* Colorless crystals (MeOH); mp 178–180 °C; ${\left[\alpha \right]}_{D}^{25}$ +56.0 (*c* 0.25, MeOH); UV (MeOH) *λ*_max_ (log *ε*) 217 (3.19) nm; ECD (0.95 mM, MeOH) *λ*_max_ (Δ*ε*) 225 (+5.42) nm; ^1^H and ^13^C NMR data, Table 1; ESIMS *m*/*z* 263 [M − H]^−^; HRESIMS *m*/*z* 263.1657 [M − H]^−^ (calcd for C_16_H_23_O_3_, 263.1653).

*Steckwaic acid F(***2***):* Colorless crystals; mp 242–244 °C; ${\left[\alpha \right]}_{D}^{25}$ +30.8 (*c* 0.26, MeOH); UV (MeOH) *λ*_max_ (log *ε*) 216 (3.12) nm; ECD (0.72 mM, MeOH) *λ*_max_ (Δ*ε*) 223 (+4.17) nm; ^1^H and ^13^C NMR data, Table 1; ESIMS *m*/*z* 277 [M − H]^−^; HRESIMS *m*/*z* 277.1439 [M − H]^−^ (calcd for C_16_H_21_O_4_, 277.1445).

*Steckwaic acid G* *(***3***):* White amorphous powder; ${\left[\alpha \right]}_{D}^{25}$ +67.9 (*c* 0.28, MeOH); UV (MeOH) *λ*_max_ (log *ε*) 213 (2.95) nm; ECD (0.95 mM, MeOH) *λ*_max_ (Δ*ε*) 223 (+3.22), 295 (+0.53) nm; ^1^H and ^13^C NMR data, Table 1; ESIMS *m*/*z* 263 [M − H]^−^; HRESIMS *m*/*z* 263.1657 [M − H]^−^ (calcd for C_16_H_23_O_3_, 263.1653).

*Steckwaic acid H(***4***):* Colorless oil; ${\left[\alpha \right]}_{D}^{25}$ −34.5 (*c* 0.29, MeOH); UV (MeOH) *λ*_max_ (log *ε*) 260 (3.00) nm; ECD (0.34 mM, MeOH) *λ*_max_ (Δ*ε*) 220 (+0.19), 295 (+0.16) nm; ^1^H and ^13^C NMR data, Table 2; ESIMS *m*/*z* 289 [M − H]^−^; HRESIMS *m*/*z* 289.1809 [M − H]^−^ (calcd for C_18_H_25_O_3_, 289.1809).

*10-Hydroxytanzawaic acid U (***5***):* White amorphous powder; ${\left[\alpha \right]}_{D}^{25}$ +50.0 (*c* 0.10, MeOH); UV (MeOH) *λ*_max_ (log *ε*) 265 (3.08) nm; ECD (1.56 mM, MeOH) *λ*_max_ (Δ*ε*) 264 (+6.12) nm; ^1^H and ^13^C NMR data, Table 2; ESIMS *m*/*z* 319 [M − H]^−^; HRESIMS *m/z* 319.1908 [M − H]^−^ (calcd for C_19_H_27_O_4_, 319.1915).

*18-O-Acetyltanzawaic acid R (***6***):* White amorphous powder; ${\left[\alpha \right]}_{D}^{25}$ +82.5 (*c* 0.40, MeOH); UV (MeOH) *λ*_max_ (log *ε*) 265 (3.32) nm; ECD (0.72 mM, MeOH) *λ*_max_ (Δ*ε*) 261 (+12.0) nm; ^1^H and ^13^C NMR data, Table 2; ESIMS *m*/*z* 347 [M − H]^−^; HRESIMS *m*/*z* 347.1861 [M − H]^−^ (calcd for C_20_H_27_O_5_, 347.1864).

*Steckwaic acid I(***7***):* White amorphous powder, ${\left[\alpha \right]}_{D}^{25}$ +165.2 (*c* 0.23, MeOH); UV (MeOH) *λ*_max_ (log *ε*) 263 (3.27) nm; ECD (0.78 mM, MeOH) *λ*_max_ (Δ*ε*) 262 (+12.2) nm; ^1^H and ^13^C NMR data, Table 3; ESIMS *m*/*z* 319 [M − H]^−^; HRESIMS *m*/*z* 319.1908 [M − H]^−^ (calcd for C_19_H_27_O_4_, 319.1915).

*13R-Tanzawaic acid S (***8***):* Colorless solid; ${\left[\alpha \right]}_{D}^{25}$ +254.5 (*c* 0.22, MeOH); UV (MeOH) *λ*_max_ (log *ε*) 265 (3.50) nm; ECD (0.78 mM, MeOH) *λ*_max_ (Δ*ε*) 266 (+17.4) nm; ^1^H and ^13^C NMR data, Table 3; ESIMS *m*/*z* 319 [M − H]^−^; HRESIMS *m*/*z* 319.1912 [M − H]^−^ (calcd for C_19_H_27_O_4_, 319.1915).

*Tanzawaic acid S (***10***):* Colorless solid; ${\left[\alpha \right]}_{D}^{25}$ +76.9 (*c* 0.13, MeOH); UV (MeOH) *λ*_max_ (log *ε*) 264 (3.39) nm; ECD (0.86 mM, MeOH) *λ*_max_ (Δ*ε*) 263 (+14.7) nm; ^1^H NMR data (DMSO-*d*_6_) *δ*_H_: 5.80 (1H, m, H-2), 7.12 (1H, dd, *J* = 14.5, 10.9 Hz, H-3), 6.27 (1H, dd, *J* = 15.3, 10.9 Hz, H-4), 5.93 (2H, m, H-5, H-14), 2.54, (1H, m, H-6), 1.23 (3H, m, H-7, H-10, H-12), 0.63 (1H, m, H-8), 1.59 (2H, m, H-9a, H-11a), 1.45 (1H, m H-9b), 1.08 (1H, m, H-11b), 3.37 (1H, dd, *J* = 6.1, 1.8 Hz, H-13), 1.57, (3H, s, H_3_-16), 0.87 (3H, d, *J* = 6.5 Hz, H_3_-17), 3.19 (2H, d, *J* = 6.2 Hz, H_2_-18), 3.22 (3H, s, H_3_-13-OCH_3_); ^13^C NMR data (DMSO-*d*_6_) *δ*_C_: 170.0 (C, C-1), 120.8 (CH, C-2), 143.7 (CH, C-3), 129.2 (CH, C-4), 148.4 (CH, C-5), 49.5 (CH, C-6), 44.2 (CH, C-7), 39.2 (CH, C-8), 39.5 (CH_2_, C-9), 40.0 (CH, C-10), 32.7 (CH_2_, C-11), 42.1 (CH, C-12), 75.3 (CH, C-13), 124.0 (CH, C-14), 139.1 (C, C-15), 22.0 (CH_3_, C-16), 21.9 (CH_3_, C-17), 66.6, (CH_2_, C-18), 55.8 (CH_3_, C-13-OCH_3_); ESIMS *m*/*z* 319 [M − H]^−^; HRESIMS *m*/*z* 319.1914 [M − H]^−^ (calcd for C_19_H_27_O_4_, 319.1915).

### 3.4. X-ray Crystallographic Analysis of Compounds **1** and **2**

Colorless crystals of compounds **1** and **2** were obtained from the solution of MeOH. Crystallographic data were collected on a Bruker D8 Venture diffractometer equipped with a graphite-monochromatic Cu K*α* radiation (*λ* = 1.54178) Å at 173 K [21]. The data were corrected for absorption by using the program SADABS [22]. The structures were solved by direct methods with the SHELXTL software package [23,24]. All non-hydrogen atoms were refined anisotropically. The H atoms connected to C atoms were calculated theoretically, and those to O atoms were assigned by difference Fourier maps. The absolute structures were determined by refinement of the Flack parameter [25]. The structures were optimized by full-matrix least-squares techniques.

*Crystal data for compound***1***:* C_16_H_24_O_3_, F.W. = 264.35, monoclinic space group *P*2(1), unit cell dimensions *a* = 5.7450(10) Å, *b* = 12.778(3) Å, *c* = 10.226(3) Å, *V* = 749.3(3) Å^3^, *α* = *γ* = 90°, *β* = 93.424(14), *Z* = 2, *d*_calcd_ = 1.172 mg/m^3^, crystal dimensions 0.200 × 0.180 × 0.160 mm, μ = 0.632 mm^−1^, *F*(000) = 288. The 2734 measurements yielded 2464 independent reflections after equivalent data were averaged. The final refinement gave *R*_1_ = 0.0421 and w*R*_2_ = 0.1125 [*I* > 2*σ*(*I*)]. The absolute structure parameter was −0.09(13).

*Crystal data for compound***2***:* C_16_H_22_O_4_, F.W. = 278.33, orthorhombic space group *P*2(1)2(1)2(1), unit cell dimensions *a* = 5.18970(10) Å, *b* = 11.7476(2) Å, *c* = 24.0950(5) Å, *V* = 1468.99(5) Å^3^, *α* = *β* = *γ* = 90°, *Z* = 4, *d*_calcd_ = 1.259 mg/m^3^, crystal dimensions 0.180 × 0.160 × 0.140 mm, μ = 0.727 mm^−1^, *F*(000) = 600. The 2681 measurements yielded 2409 independent reflections after equivalent data were averaged. The final refinement gave *R*_1_ = 0.0371 and w*R*_2_ = 0.0956 [*I* > 2*σ*(*I*)]. The absolute structure parameter was 0.05(10).

### 3.5. Computational Section

Conformational searches were performed via molecular mechanics using the MMFF method in Macromodel software, and the geometries were reoptimized at the B3LYP/6-31G(d) PCM/MeOH level using Gaussian 09 software to obtain the energy-minimized conformers [26]. Then, the optimized conformers were subjected to calculate the ECD spectra using TDDFT at the BH&HLYP/TZVP, CAM-B3LYP/TZVP, and PBE0/TZVP levels. The solvent effects of the MeOH solution were evaluated at the same DFT level using the SCRF/PCM method.

### 3.6. Antimicrobial Assays

The antimicrobial activities against one human pathogenic bacterium (*Escherichia coli* EMBLC-1), nine aquatic pathogens (*Aeromonas hydrophilia* QDIO-1, *E. tarda* QDIO-2, *Micrococcus luteus* QDIO-3, *Pseudomonas aeruginosa* QDIO-4, *Vibro alginolyticus* QDIO-5, *V. anguillarum* QDIO-6, *V. harveyi* QDIO-7, *V. parahaemolyticus* QDIO-8, and *V. vulnificus* QDIO-10), as well as seven plant-pathogenic fungi (*Alternaria solani* QDAU-1, *Colletotrichum gloeosporioides* QDAU-2, *Fusarium graminearum* QDAU-4, *F. oxysporum* QDAU-8, *Gaeumannomyces graminis* QDAU-21, *Rhizoctonia cerealis* QDAU-20, and *Valsa mali* QDAU-16), were determined by a serial dilution technique using 96-well microtiter plates [27]. The aquatic pathogenic strains and human pathogenic bacteria were obtained from IOCAS. Tested compounds and the positive control (chloramphenicol for bacteria and amphotericin B for fungi) were dissolved in DMSO to produce a stock solution.

## 4. Conclusions

In conclusion, eight new polyketide derivatives (**1**–**8**) and two related known analogues (**9** and **10**) were identified from the endozoic fungus *P. steckii* AS-324, which was obtained from the fresh tissues of deep-sea *Acanthogorgiidae* sp. coral collected from Magellan seamount in the Western Pacific Ocean at a depth of 1458 m. Compounds **1**–**3**, with an acrylic acid side chain at C-4, have been rarely observed [28], and the double bond between C-12 and C-13 in compound **4** is a new observation. Antimicrobial activities of compounds **1**–**10** were tested, and compound **8** showed potent activity against *E. coli* with an MIC value of 8 μg/mL while compound **10** exhibited inhibitory activity against *E. tarda* with an MIC value of 16 μg/mL.

## Figures and Tables

**Table 1:** ^1^H and ^13^C NMR data of compounds **1**–**3** in DMSO-*d*_6_.

No.	1		2		3	
	*δ*_C_, Type	*δ*_H_ (*J* in Hz)	*δ*_C_, Type	*δ*_H_ (*J* in Hz)	*δ*_C_, Type	*δ*_H_ (*J* in Hz)
1	167.6, C		167.4, C *^a^*		167.6, C	
2	120.5, CH	5.71, d (15.4)	120.1, CH *^b^*	5.74, d (15.3)	120.5, CH	5.71, d (15.4)
3	154.2, CH	6.84, dd (15.4, 10.9)	154.2, CH	6.88, dd (15.3, 10.9)	153.2, CH	6.86, dd (15.4, 10.9)
4	48.0, CH	2.46, ddd (10.9, 9.5, 5.6)	47.5, CH	2.46, td (10.9, 5.0)	47.2, CH	2.45, td (10.9, 5.4)
5	44.9, CH	1.00, q (9.5)	46.0, CH	0.98, m	46.3, CH	0.88, overlap
6	35.8, CH	2.11, m	35.7, CH	1.37, m	35.4, CH	1.32, dddq (10.5, 10.1, 3.2, 6.4)
7	44.4, CH_2_	1.56, dt (13.7, 3.8) 0.83, overlap	40.0, CH_2_	1.81, overlap 1.11, overlap	40.5, CH_2_	1.66, dq (14.3, 3.2) 0.72, overlap
8	40.0, CH	1.35, overlap	43.2, CH	2.35, m	40.0, CH	1.52, m
9	77.0, CH	2.59, t (9.5)	35.4, CH_2_	1.93, d (12.3) 1.11, overlap	35.8, CH_2_	1.77, overlap 0.72, overlap
10	49.2, CH	1.67, td (9.5, 2.0)	41.5, CH	1.81, overlap	41.6, CH	1.77, overlap
11	129.4, CH	6.01, dt (9.8, 2.0)	131.7, CH	5.45, br d (9.7)	131.8, CH	5.44, dt (9.6, 2.0)
12	132.2, CH	5.58, ddd (9.8, 4.3, 2.0)	132.6, CH	5.58, br d (9.7)	131.9, CH	5.56, ddd (9.6, 4.4, 2.0)
13	36.1, CH	1.35, overlap	36.7, CH	2.15, q (6.1)	36.3, CH	2.14, m
14	16.6, CH_3_	0.88, d (7.1)	16.5, CH_3_	0.90, overlap	16.1, CH_3_	0.90, d (7.0)
15	22.3, CH_3_	0.83, overlap	22.6, CH_3_	0.90, overlap	22.5, CH_3_	0.88, d (6.4)
16	19.2, CH_3_	0.91, d (6.4)	176.9, C *^a^*		66.3, CH_2_	3.20, m
1-COOH		12.09, br s		12.02, br s		
9-OH		4.56, br s				

*^a^* Detected by HMBC data. *^b^* Detected by HSQC data.

**Table 2:** ^1^H and ^13^C NMR data of compounds **4**–**6** in DMSO-*d*_6_.

No.	4		5		6	
	*δ*_C_, Type	*δ*_H_ (*J* in Hz)	*δ*_C_, Type	*δ*_H_ (*J* in Hz)	*δ*_C_, Type	*δ*_H_ (*J* in Hz)
1	167.6, C		167.7, C		167.8, C *^a^*	
2	120.6, CH	5.80, d (15.3)	120.4, CH	5.77, d (15.2)	120.7, CH	5.79, d (15.3)
3	144.2, CH	7.13, dd (15.3, 10.8)	144.4, CH	7.14, dd (15.2, 11.1)	144.0, CH	7.11, dd (15.3, 11.0)
4	128.8, CH	6.23, dd (15.1, 10.8)	129.2, CH	6.29 dd (15.3, 11.1)	129.3, CH	6.31, dd (15.3, 11.0)
5	144.9, CH	6.11, dd (15.1, 9.3)	148.9, CH	5.95, overlap	148.9, CH	6.00, dd (15.3, 9.4)
6	43.6, CH	2.52, ddd (10.9, 9.3, 2.3)	49.4, CH	2.53, m	49.5, CH	2.52, m
7	49.4, CH	1.39, dd (10.9, 7.5)	43.6, CH	1.19, m	43.1, CH	1.29, overlap
8	34.5, CH	1.48, overlap	34.6, CH	1.41, overlap	39.2, CH	1.57, dd (12.3, 2.9)
9	44.7, CH_2_	1.70, m 0.89, m	48.7, CH_2_	1.41, overlap 0.98, m	39.5, CH_2_	1.29, overlap 0.73, q (12.3)
10	36.3, CH	1.48, overlap	68.0, C		36.1, CH	1.71, m
11	44.0, CH_2_	2.14, ddd (12.1, 3.8, 1.8) 1.59, m	41.9, CH_2_	1.62, overlap 1.41, overlap	32.4, CH_2_	*α* 1.15, overlap *β* 1.52, m
12	138.8, C		37.6, CH	1.62, overlap	42.2, CH	1.15, overlap
13	124.2, CH	5.31, d (2.0)	75.2, CH	3.33, m	65.5, CH	3.66, d (6.0)
14	68.7, CH	3.59, m	124.1, CH	5.95, overlap	127.1, CH	5.74, dt (6.0, 1.5)
15	36.2, CH	1.48, overlap	139.1, C		136.1, C	
16	15.6, CH_3_	0.90, d (6.8)	22.0, CH_3_	1.58, s	21.9, CH_3_	1.52, s
17	20.4, CH_3_	0.92, d (6.3)	21.5, CH_3_	0.81, d (6.6)	22.1, CH_3_	0.88, d (5.9)
18	22.1, CH_3_	0.87, d (6.5)	31.5, CH_3_	1.08, s	68.7, CH_2_	3.82, d (6.4)
13-OCH_3_			55.8, CH_3_	3.22, s		
19					170.4, C	
20					20.7, CH_3_	2.00, s
1-COOH				12.12, br s		

*^a^* Detected by HMBC correlations.

**Table 3:** ^1^H and ^13^C NMR data of compounds **7** and **8** in DMSO-*d*_6_.

No.	7		8	
	*δ*_C_, Type	*δ*_H_ (*J* in Hz)	*δ*_C_, Type	*δ*_H_ (*J* in Hz)
1	168.8, C *^a^*		168.2, C	
2	123.9, CH	5.82, d (15.4)	121.2, CH	5.80, d (15.3)
3	141.8, CH	7.01, dd (15.4, 11.1)	144.5, CH	7.12, dd (15.3, 11.0)
4	129.6, CH	6.22, td (11.1, 5.2)	129.6, CH	6.28, dd (15.3, 11.0)
5	146.6, CH	5.78, dd (11.1, 5.2)	149.0, CH	5.94, dd (15.3, 9.2)
6	49.5, CH	2.53, m	49.6, CH	2.57, t (9.2)
7	40.0, CH	1.57, overlap	48.7, CH	0.99, m
8	42.1, CH	1.23, overlap	39.2, CH	1.37, overlap
9	39.2, CH_2_	1.23, overlap 0.63, dd (12.2, 5.6)	40.0, CH_2_	1.61, d (12.5) 0.70, q (12.5)
10	39.5, CH	1.46, m	39.2, CH	1.37, overlap
11	32.8, CH_2_	1.57, overlap 1.07, m	32.9, CH_2_	*α* 2.17, d (12.3) *β* 0.57, q (12.3)
12	44.3, CH	1.23, overlap	43.8, CH	1.10, m
13	75.3, CH	3.37, d (6.1)	80.3, CH	3.32, d (9.4)
14	123.9, CH	5.91, d (6.1)	126.3, CH	5.64, m
15	139.4, C		134.7, C	
16	22.0, CH_3_	1.58, s	21.9, CH_3_	1.55, s
17	22.0, CH_3_	0.87, d (6.5)	22.9, CH_3_	0.87 d (6.5)
18	66.6, CH_2_	3.18, d (6.1)	66.9, CH_2_	3.19, d (6.2)
13-OCH_3_	55.8, CH_3_	3.22, s	55.7, CH_3_	3.26, s
18-OH				4.35, br s

*^a^* Detected by HMBC correlations.

**Table 4:** Antibacterial activities of active compounds **5** and **8**−**10** (MIC, μg/mL) *^a^*.

	*E. tarda*	*E. coli*	*V. parahaemolyticus*	*V. vulnificus*
**5**	-	-	16	-
**8**	64	8	-	-
**9**	-	-	64	-
**10**	16	-	-	-
Chloramphenicol *^b^*	2	1	1	2

*^a^* (-) = MIC > 64 μg/mL. *^b^* Chloramphenicol as positive control.

## Data Availability

Not applicable.

## Supplementary Materials

The following supporting information can be downloaded at: https://www.mdpi.com/article/10.3390/ijms23116332/s1.
