# Supplementary material for: Uncommon Polyketides from Penicillium steckii AS-324, a Marine Endozoic Fungus Isolated from Deep-Sea Coral in the Magellan Seamount
Source: Int J Mol Sci. 2022 Jun 6;23(11):6332. doi: 10.3390/ijms23116332 (PMC9181172; doi:10.3390/ijms23116332)
Supplement: Supplementary file 1 [file ijms-23-06332-s001.zip › ijms-1729047-supplementary.pdf]

# Supporting Information

# Uncommon Polyketides from *Penicillium steckii* AS-324, a Marine Endozoic Fungus Isolated from Deep-Sea Coral in the Magellan Seamount

**Xue-Yi Hu [1,2,3], Xiao-Ming Li [1,3], Bin-Gui Wang [1,2,3,4,*] and Ling-Hong Meng [1,2,4,*]**

[1] CAS and Shandong Province Key Laboratory of Experimental Marine Biology, Institute of Oceanology, Chinese Academy of Sciences, Nanhai Road 7, Qingdao 266071, China; xueyihu61@163.com (X.-Y.H.); lixmqd@qdio.ac.cn (X.-M.L.)
[2] Laboratory of Marine Biology and Biotechnology, Qingdao National Laboratory for Marine Science and Technology, Wenhai Road 1, Qingdao 266237, China
[3] College of Marine Science, University of Chinese Academy of Sciences, Yuquan Road 19A, Beijing 100049, China
[4] Center for Ocean Mega-Science, Chinese Academy of Sciences, Nanhai Road 7, Qingdao 266071, China
* Correspondence: wangbg@ms.qdio.ac.cn (B.-G.W.); menglh@ms.qdio.ac.cn (L.-H.M.);
Tel.: +86-532-8289-8553 (B.-G.W.); +86-532-8289-8890 (L.-H.M.)

## Content

**Figure S1.** HRESI mass spectrum of compound **1**;

**Figure S2.** $^{1}$H NMR (500 MHz, DMSO-$d_6$)) spectrum of compound **1**;

**Figure S3.** $^{13}$C NMR (125 MHz, DMSO-$d_6$) and DEPT spectra of compound **1**;

**Figure S4**. COSY spectrum of compound **1**;

**Figure S5.** HSQC spectrum of compound **1**;

**Figure S6**. HMBC spectrum of compound **1**;

**Figure S7**. NOESY spectrum of compound **1**;

**Figure S8.** HRESI mass spectrum of compound **2**;

**Figure S9**. $^{1}$H NMR (500 MHz, DMSO-$d_6$) spectrum of compound **2**;

**Figure S10.** $^{13}$C NMR (125 MHz, DMSO-$d_6$) and DEPT spectra of compound **2**;

**Figure S11**. COSY spectrum of compound **2**;

**Figure S12**. HSQC spectrum of compound **2**;

**Figure S13.** HMBC spectrum of compound **2**;

**Figure S14**. NOESY spectrum of compound **2**;

**Figure S15**. HRESI mass spectrum of compound **3**;

**Figure S16**. $^{1}$H NMR (500 MHz, DMSO-$d_6$) spectrum of compound **3**;

**Figure S17**. $^{13}$C NMR (125 MHz, DMSO-$d_6$) and DEPT spectra of compound **3**;

**Figure S18.** COSY spectrum of compound **3**;

**Figure S19**. HSQC spectrum of compound **3**;

**Figure S20**. HMBC spectrum of compound **3**;

**Figure S21.** NOESY spectrum of compound **3**;

**Figure S22**. HRESI mass spectrum of compound **4**;

**Figure S23**. $^{1}$H NMR (500 MHz, DMSO-$d_6$) spectrum of compound **4**;

**Figure S24**. $^{13}$C NMR (125 MHz, DMSO-$d_6$) and DEPT spectra of compound **4**;

**Figure S25**. COSY spectrum of compound **4**;

**Figure S26.** HSQC spectrum of compound **4**;

**Figure S27**. HMBC spectrum of compound **4**;

**Figure S28.** NOESY spectrum of compound **4**;

**Figure S29**. HRESI mass spectrum of compound **5**;

**Figure S30**. $^{1}$H NMR (500 MHz, DMSO-$d_6$) spectrum of compound **5**;

**Figure S31**. $^{13}$C NMR (125 MHz, DMSO-$d_6$) and DEPT spectra of compound **5**;

**Figure S32**. COSY spectrum of compound **5**;

**Figure S33.** HSQC spectrum of compound **5**;
**Figure S34**. HMBC spectrum of compound **5**;
**Figure S35**. NOESY spectrum of compound **5**;
**Figure S36**. HRESI mass spectrum of compound **6**;
**Figure S37**. $^{1}$H NMR (500 MHz, DMSO-$d_6$) spectrum of compound **6**;
**Figure S38**. $^{13}$C NMR (125 MHz, DMSO-$d_6$) and DEPT spectra of compound **6**;
**Figure S39**. COSY spectrum of compound **6**;
**Figure S40.** HSQC spectrum of compound **6**;
**Figure S41**. HMBC spectrum of compound **6**;
**Figure S42**. NOESY spectrum of compound **6**
**Figure S43**. HRESI mass spectrum of compound **7**;
**Figure S44**. $^{1}$H NMR (500 MHz, DMSO-$d_6$) spectrum of compound **7**;
**Figure S45**. $^{13}$C NMR (125 MHz, DMSO-$d_6$) and DEPT spectra of compound **7**;
**Figure S46**. COSY spectrum of compound **7**;
**Figure S47.** HSQC spectrum of compound **7**;
**Figure S48**. HMBC spectrum of compound **7**;
**Figure S49**. NOESY spectrum of compound **7**;
**Figure S50**. HRESI mass spectrum of compound **8**;
**Figure S51**. $^{1}$H NMR (500 MHz, DMSO-$d_6$) spectrum of compound **8**;
**Figure S52**. $^{13}$C NMR (125 MHz, DMSO-$d_6$) and DEPT spectra of compound **8**;
**Figure S53**. COSY spectrum of compound **8**;
**Figure S54.** HSQC spectrum of compound **8**;
**Figure S55**. HMBC spectrum of compound **8**;
**Figure S56**. NOESY spectrum of compound **8**;
**Figure S57**. Structures and populations of conformers obtained by gas phase B3LYP/6-31G(d) reoptimization of the initial MMFF conformers of (6*R*, 7*R*, 8*R*, 10*S*, 14*S*, and 15*R*)-**4** above 2% population;
**Figure S58**. Structures and populations of conformers obtained by gas phase B3LYP/6-31G(d) reoptimization of the initial MMFF conformers of (6*R*, 7*R*, 8*R*, 10*R*, 12*S*, and 13*S*)-**5** above 2% population;
**Figure S59**. Experimental and calculated ECD at different levels of compounds **4** and **5**;
**Table S1**. Cartesian coordinates for the low-energy reoptimized MMFF conformers of **4** calculated at B3LYP/6-31G(d) level of theory in vacuo;

**Table S2**. Cartesian coordinates for the low-energy reoptimized MMFF conformers of **5** calculated at B3LYP/6-31G(d) level of theory in vacuo.

**Figure S1.** HRESI mass spectrum of compound **1**;

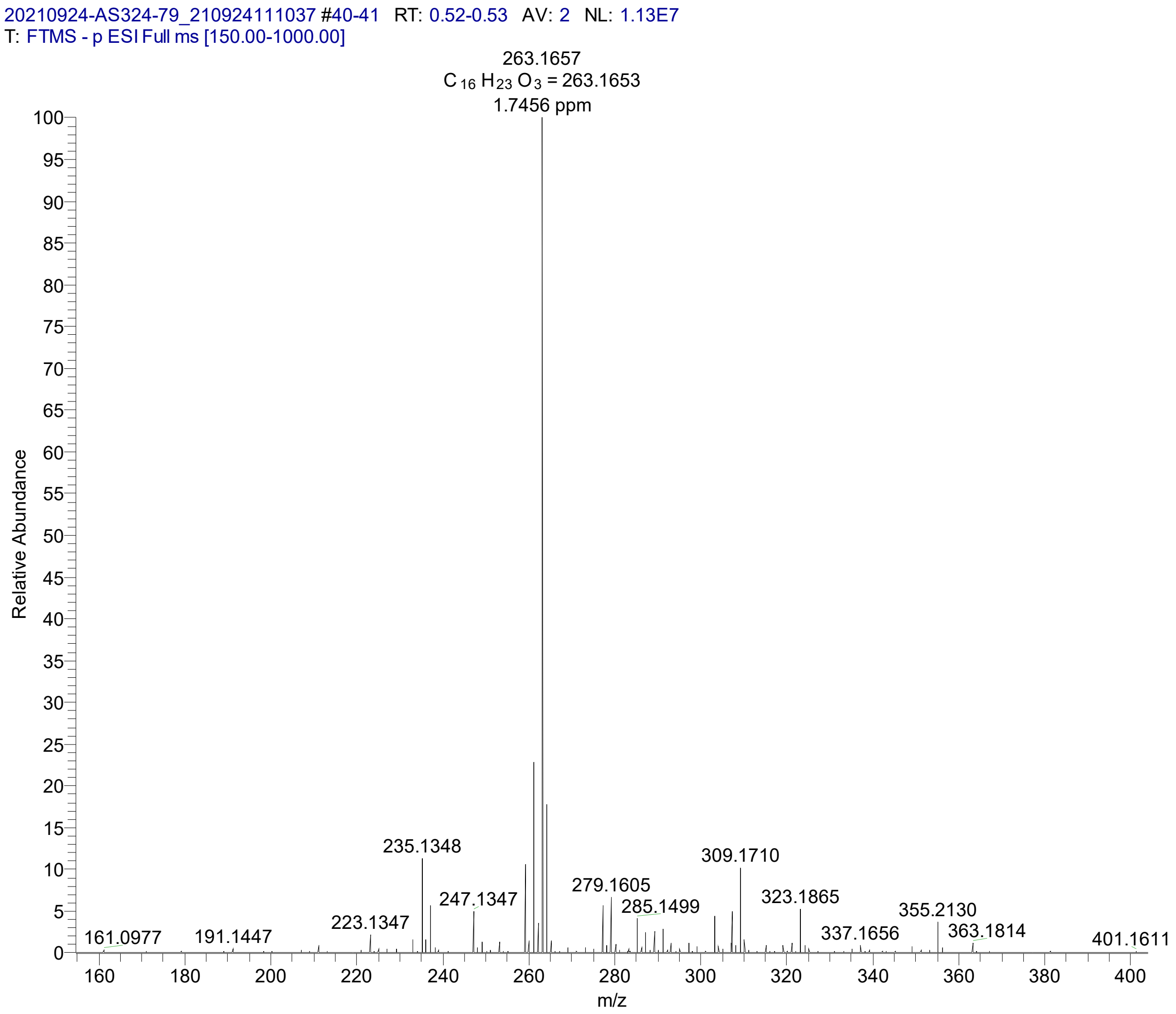

**Figure S2.** $^{1}H$ NMR (500 MHz, DMSO-$d_6$)) spectrum of compound **1**;

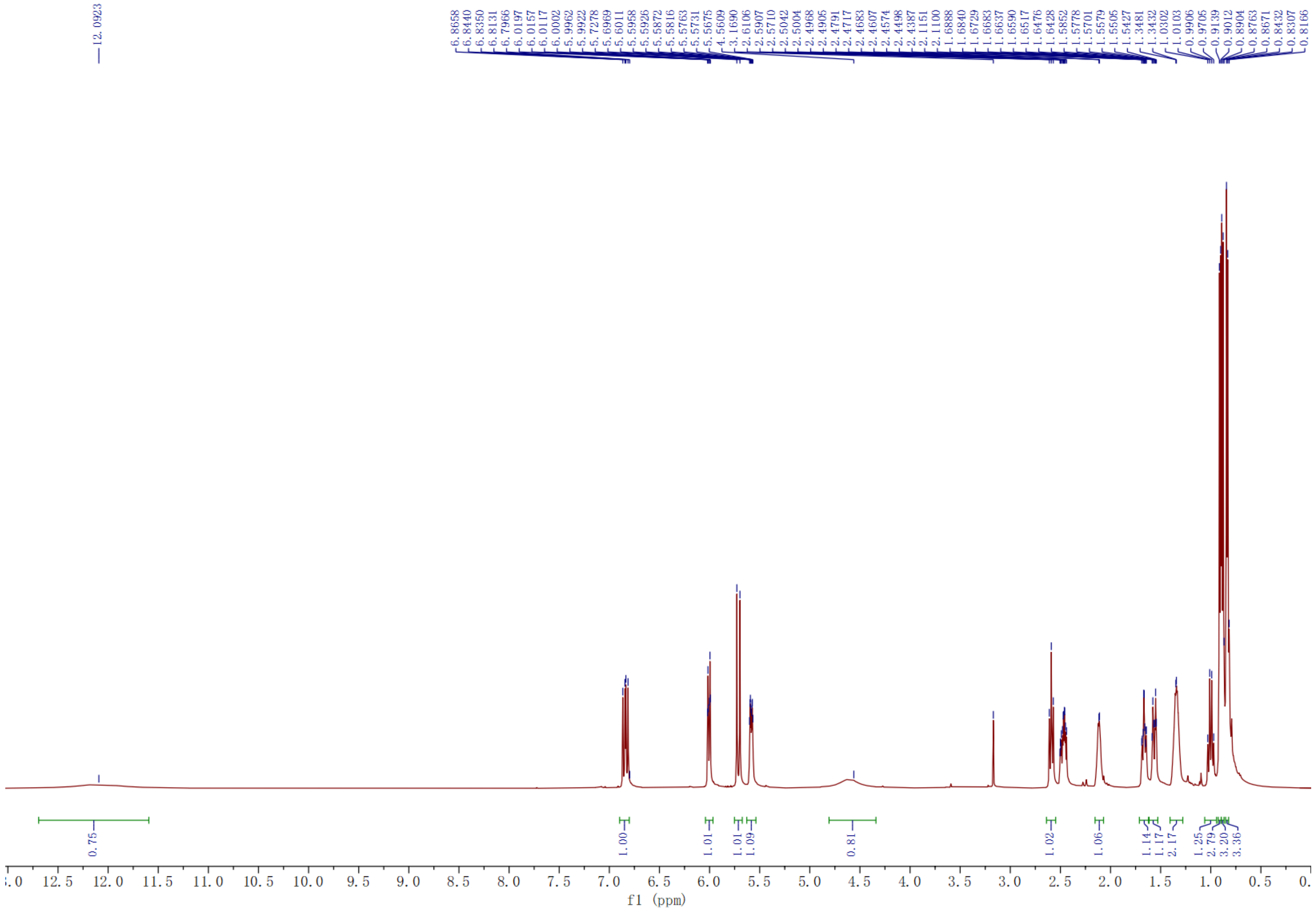

**Figure S3.** $^{13}$C NMR (125 MHz, DMSO-$d_6$) and DEPT spectra of compound **1**;

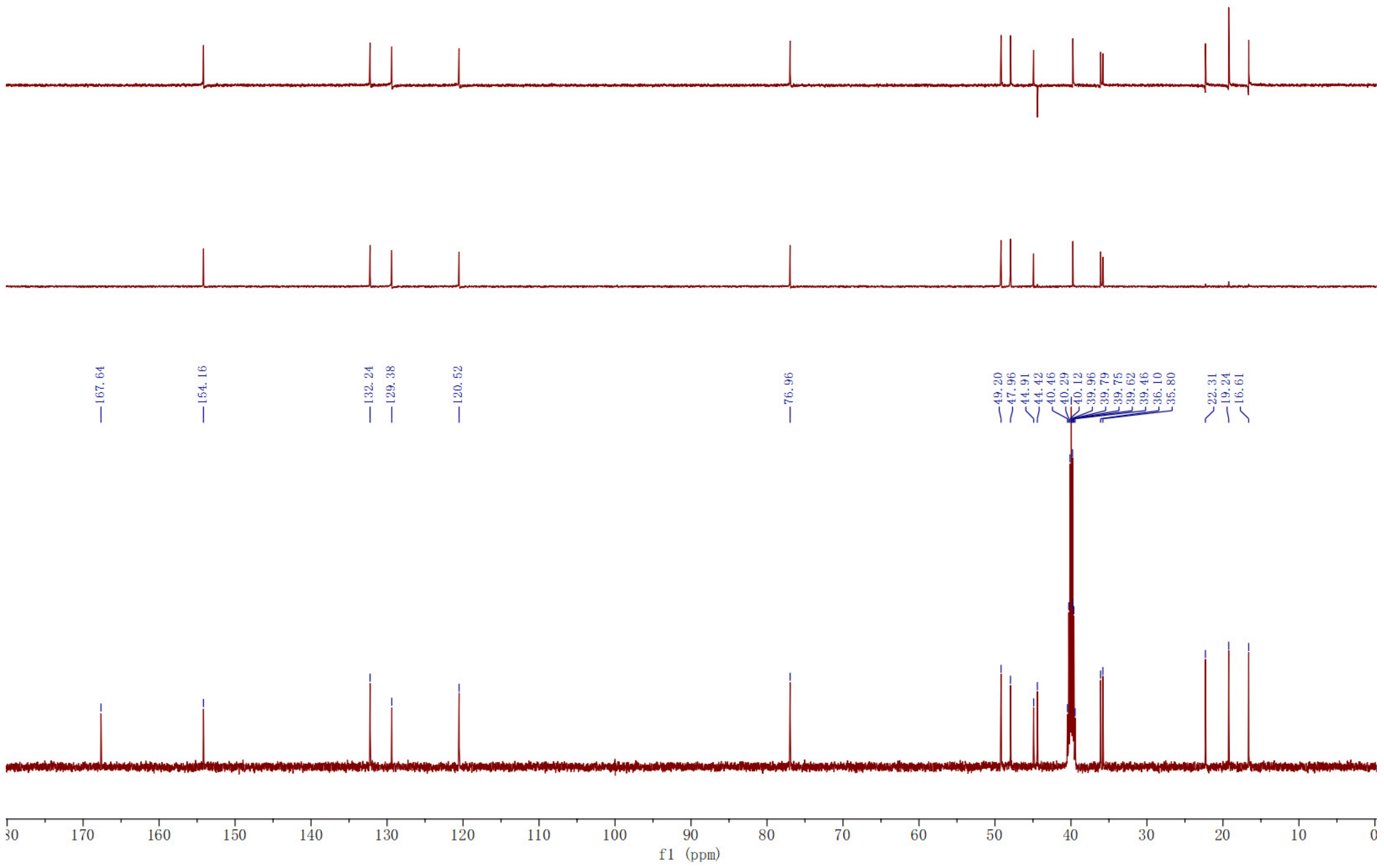

**Figure S4**. COSY spectrum of compound **1**;

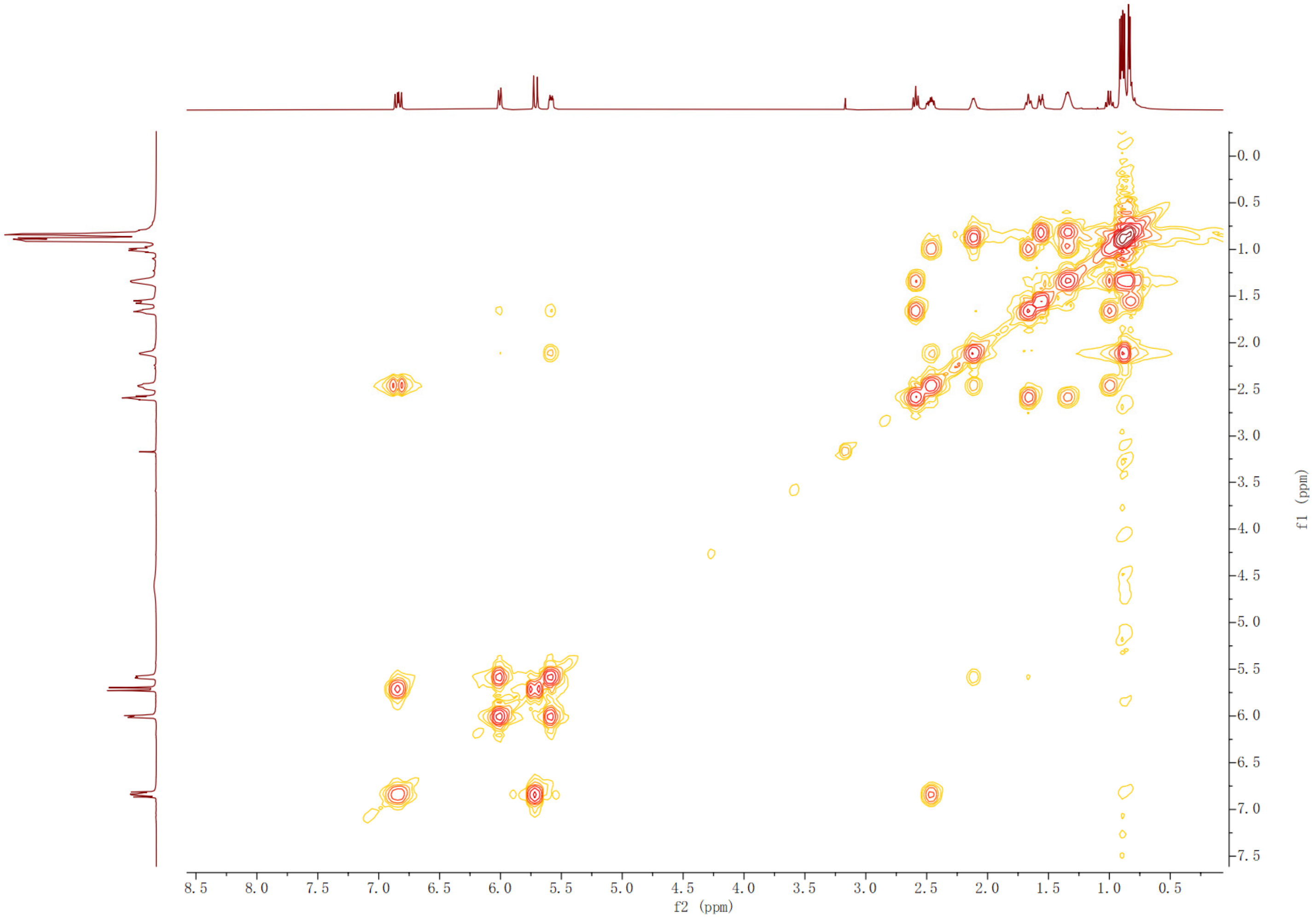

**Figure S5.** HSQC spectrum of compound **1**;

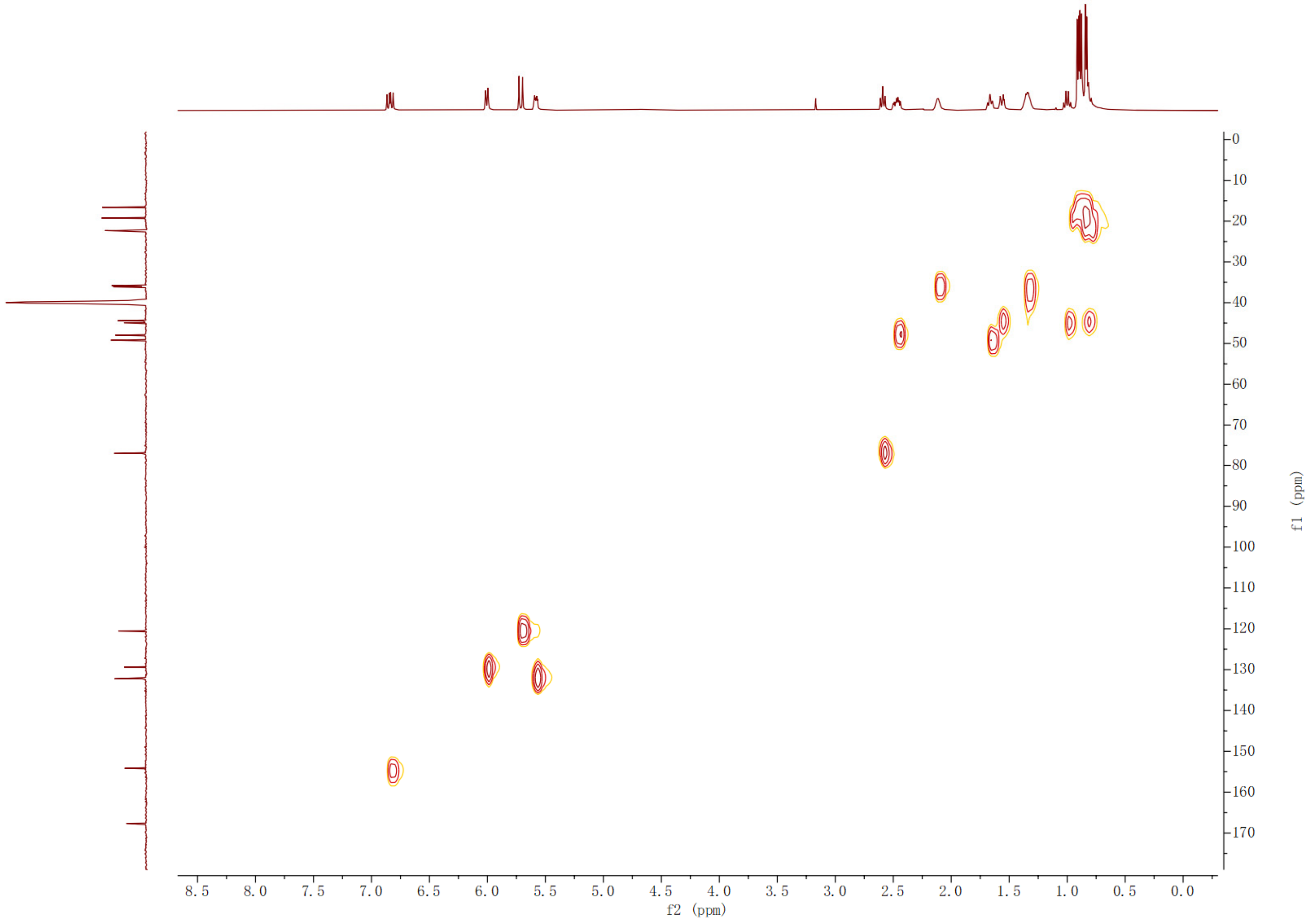

**Figure S6**. HMBC spectrum of compound **1**;

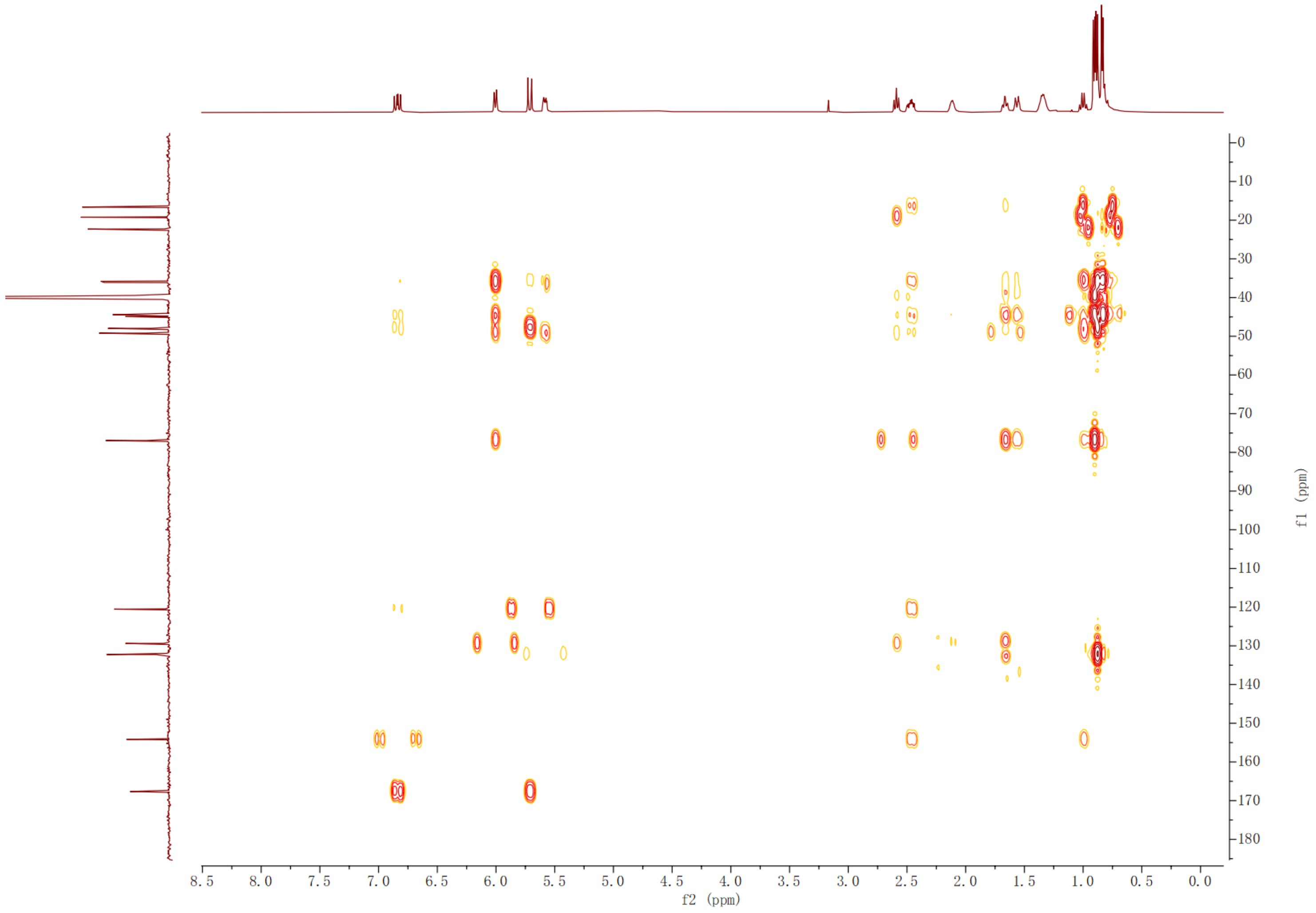

**Figure S7**. NOESY spectrum of compound **1**;

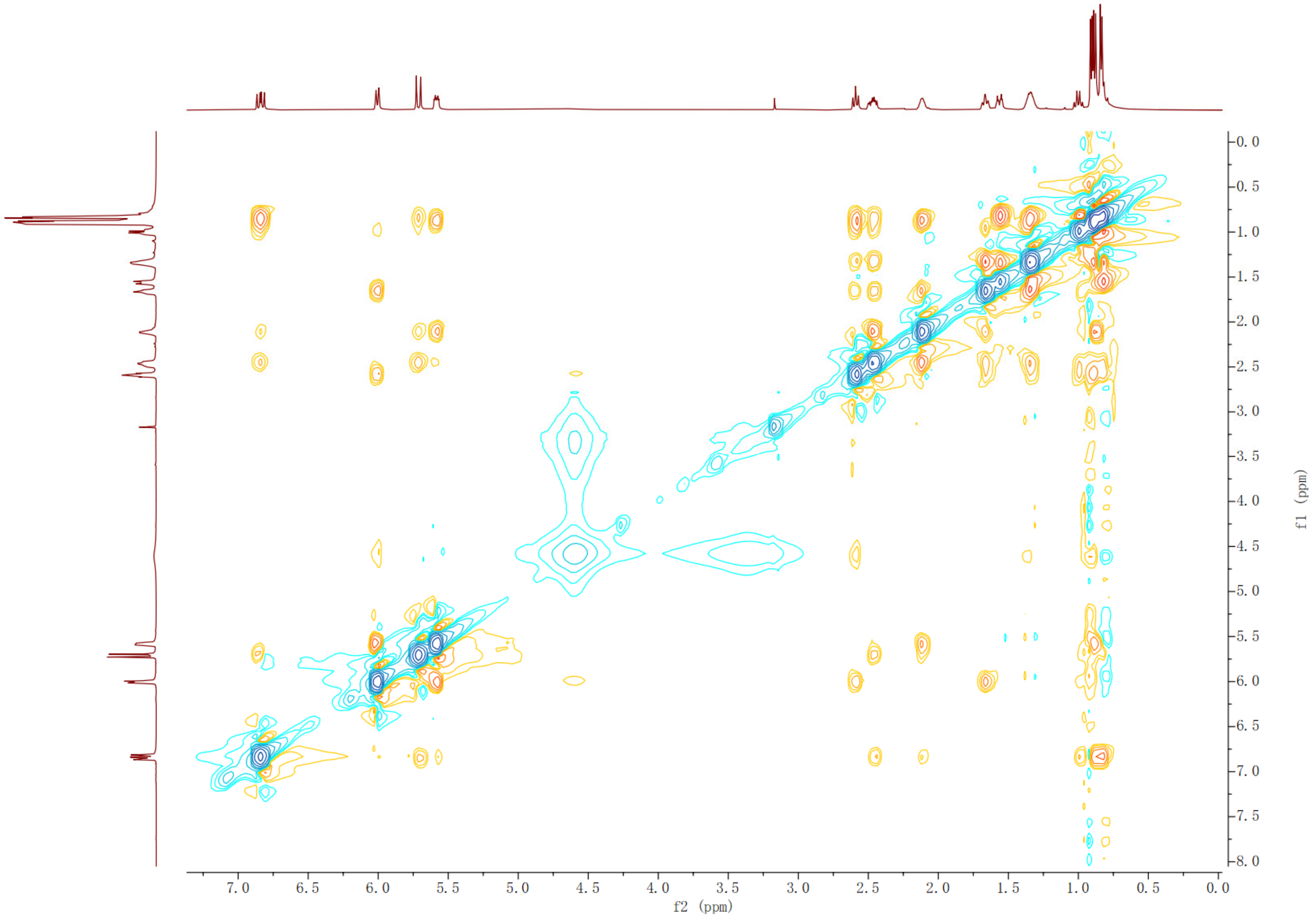

**Figure S8**. HRESI mass spectrum of compound **2**;

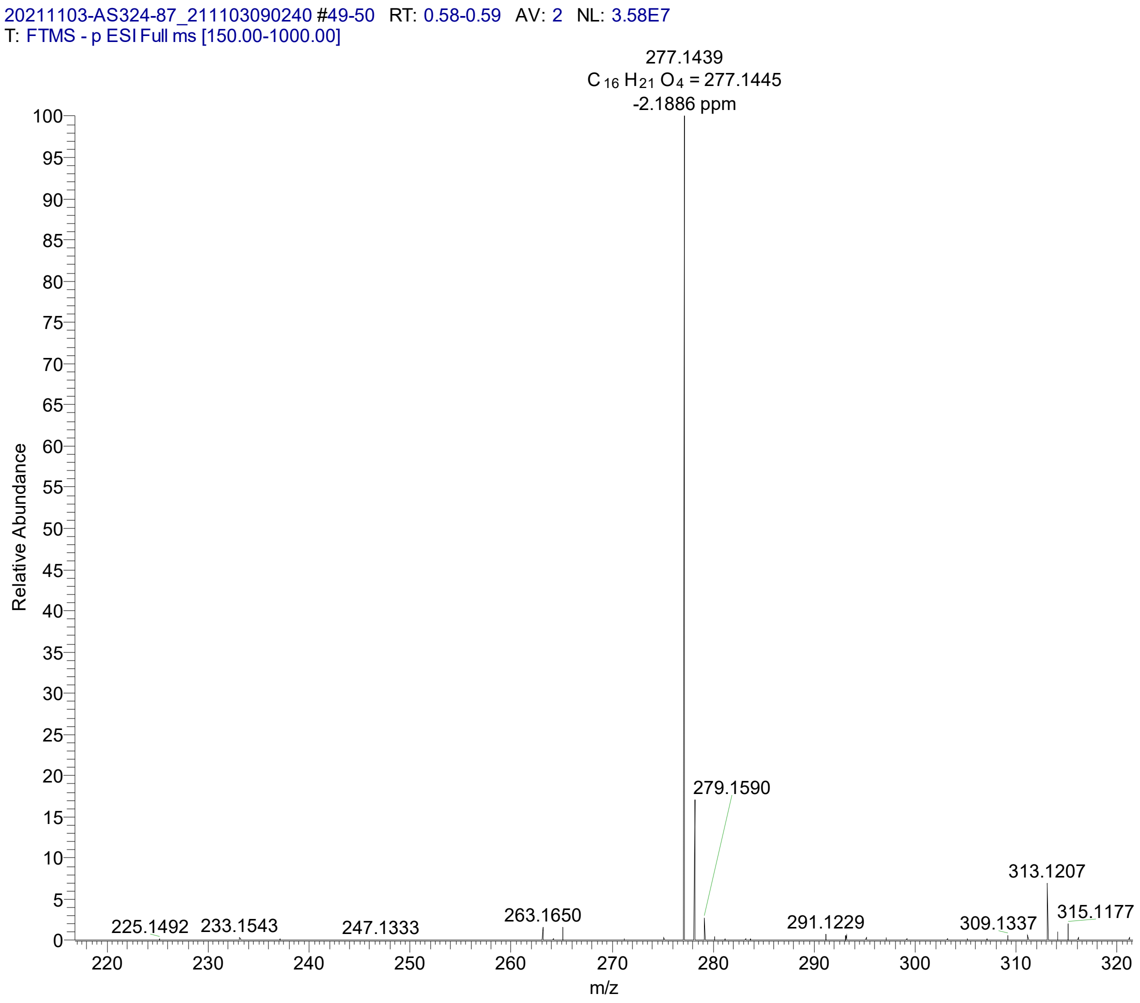

**Figure S9**. $^{1}H$ NMR (500 MHz, DMSO-$d_6$) spectrum of compound **2**;

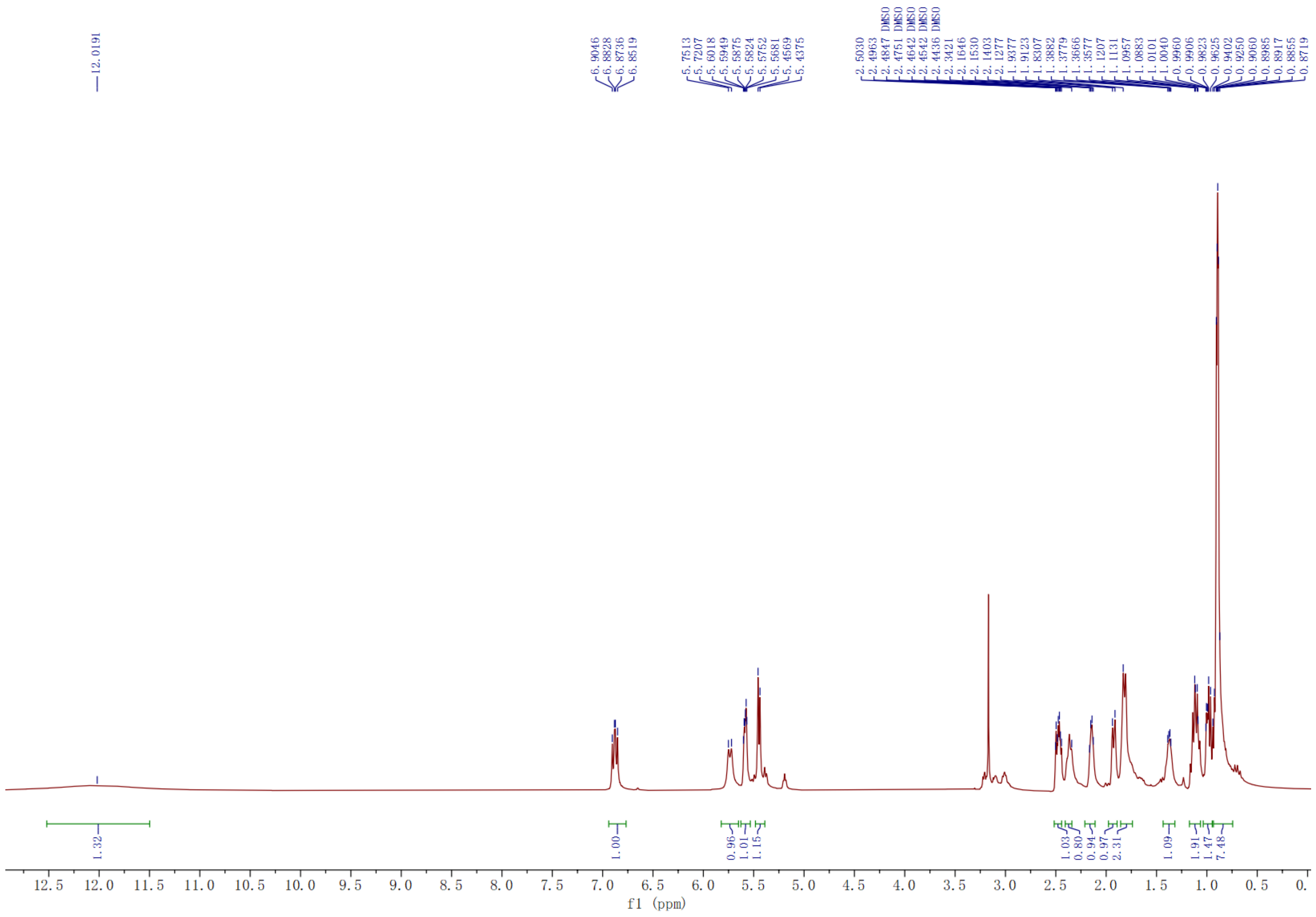

**Figure S10**. $^{13}$C NMR (125 MHz, DMSO-$d_6$) and DEPT spectra of compound **2**;

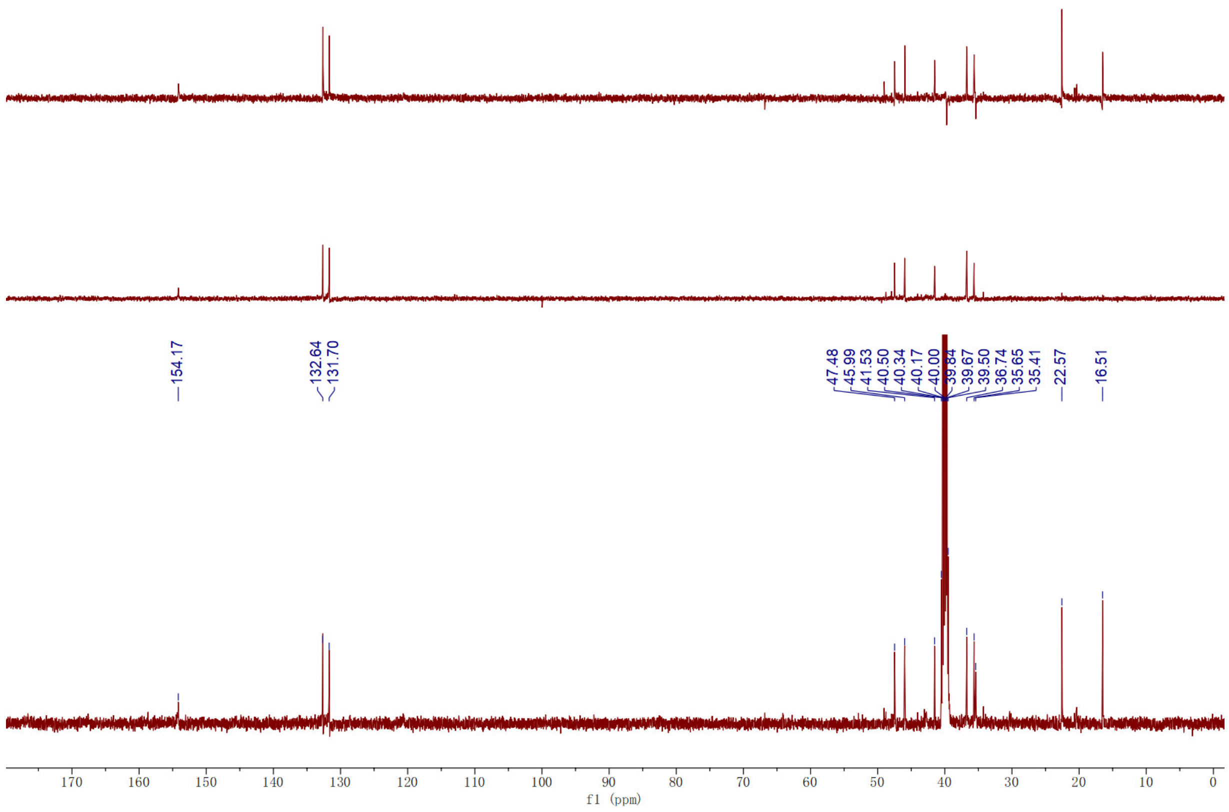

**Figure S11.** COSY spectrum of compound **2**;

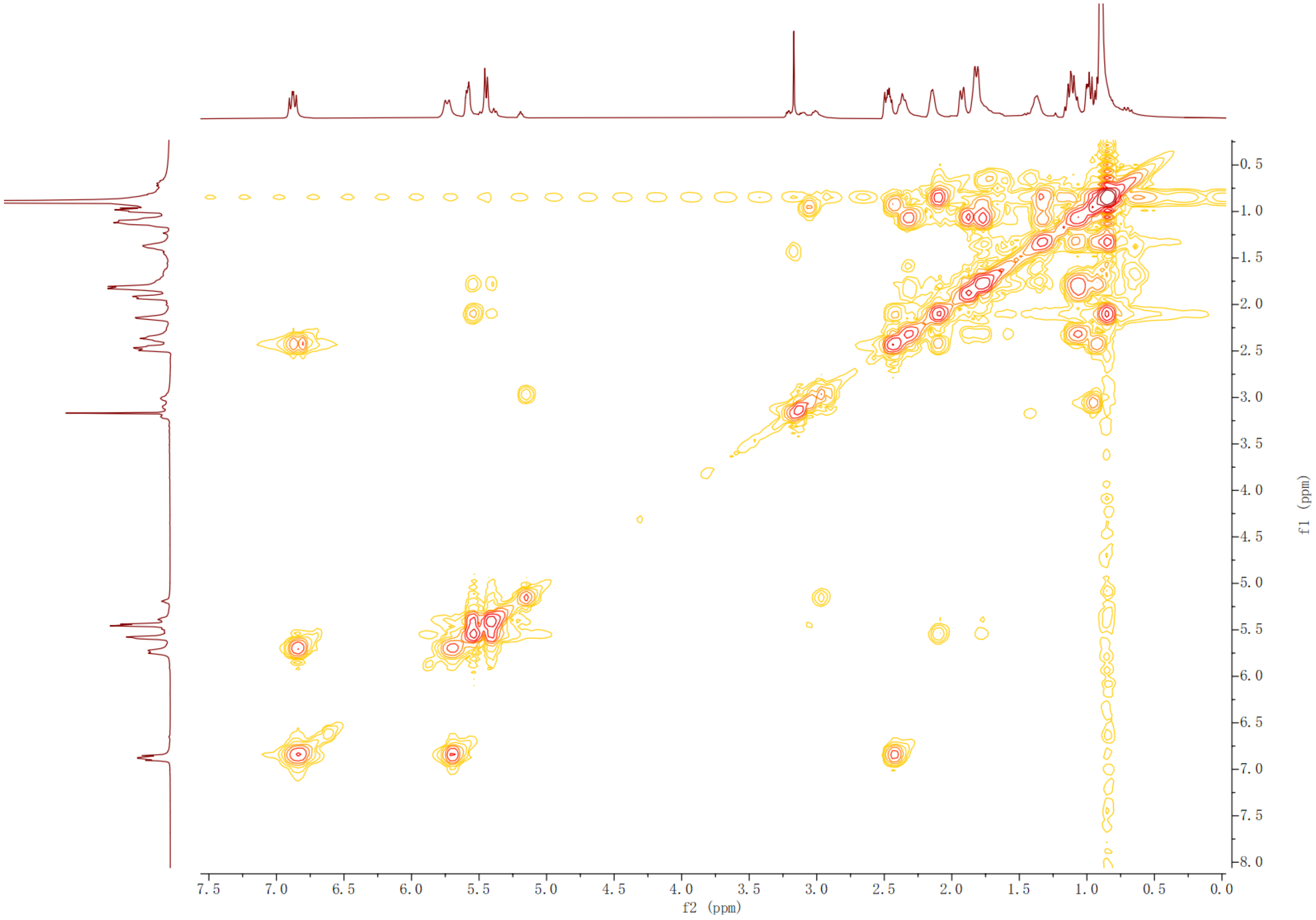

**Figure S12**. HSQC spectrum of compound **2**;

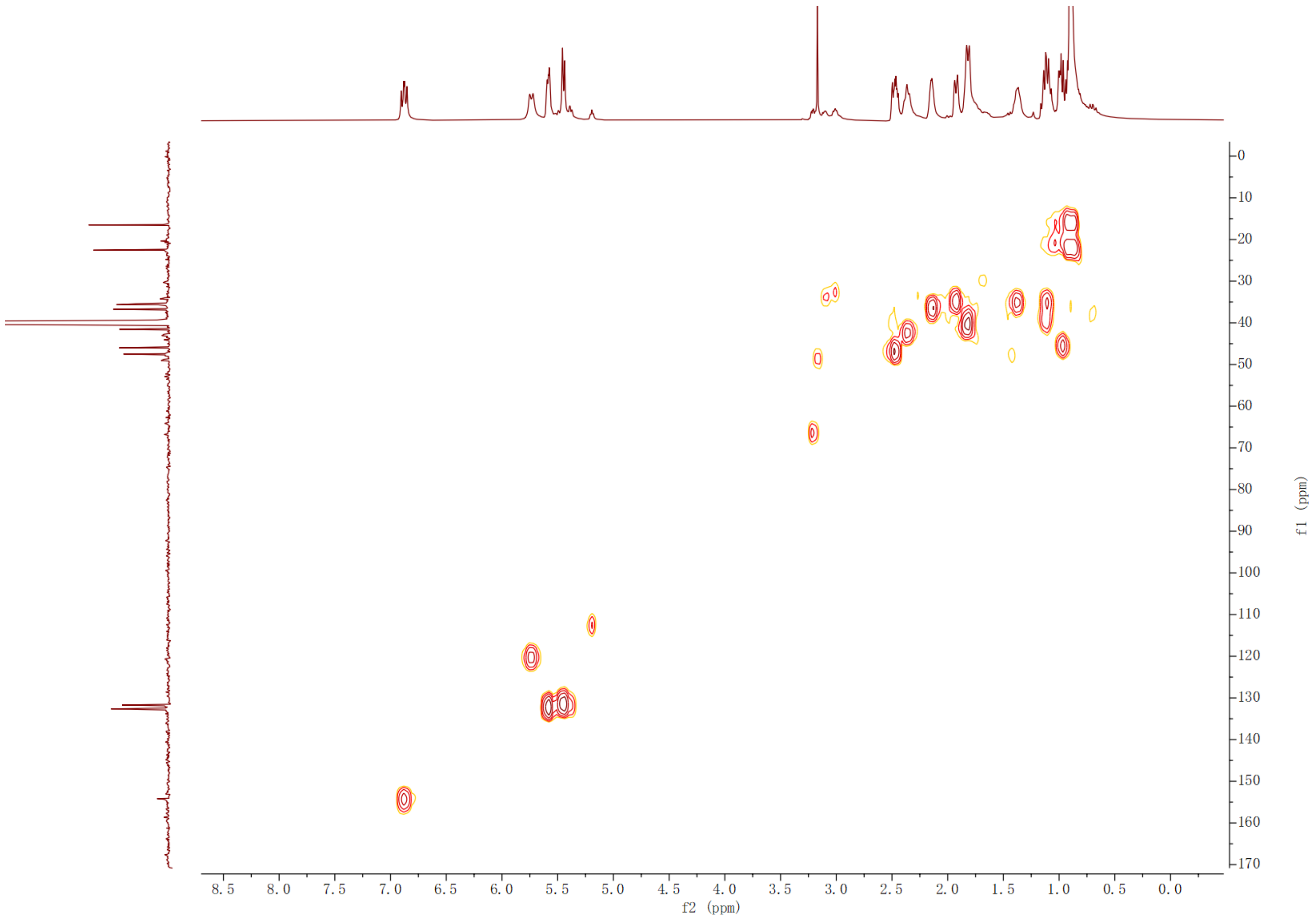

**Figure S13**. HMBC spectrum of compound **2**;

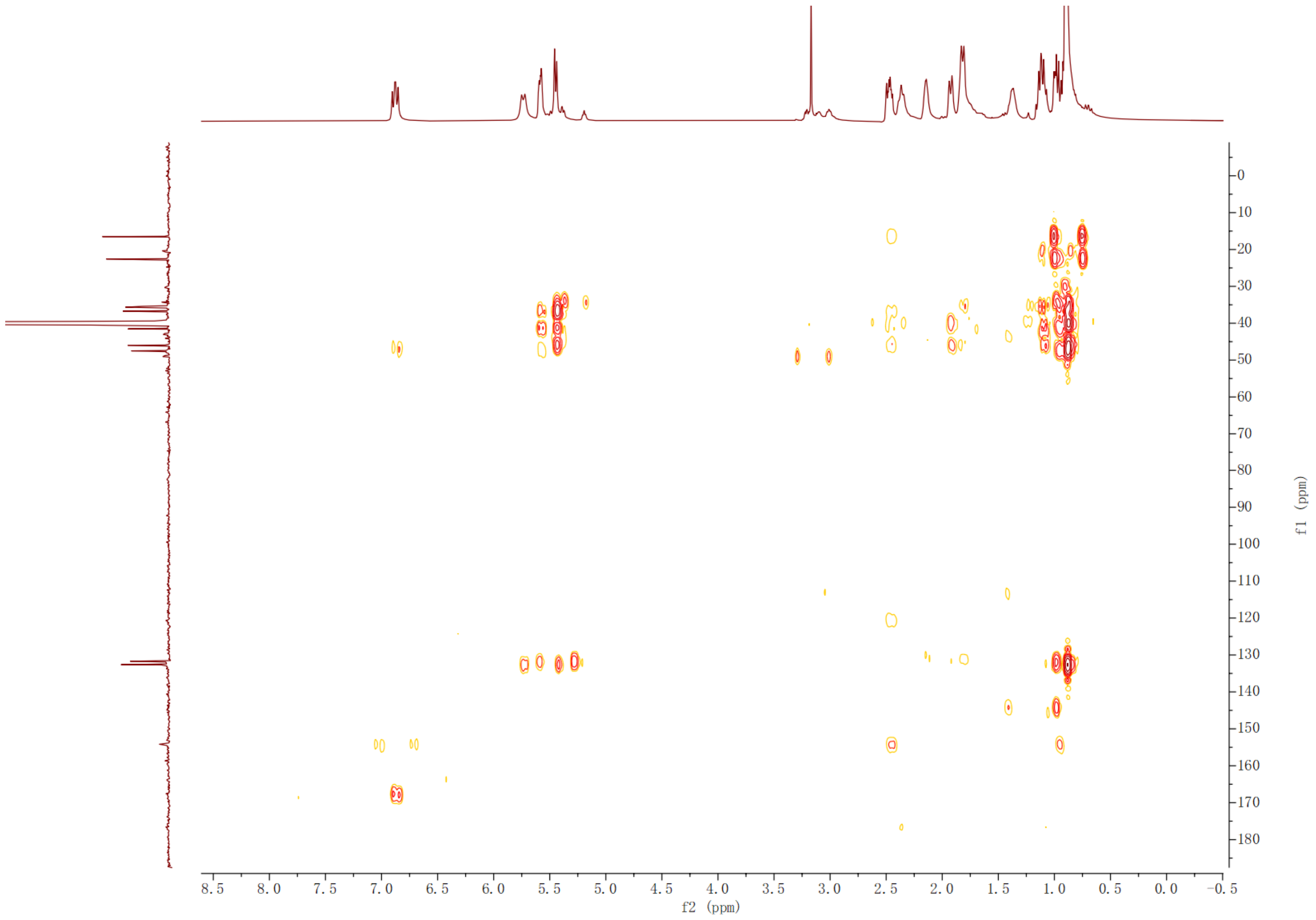

**Figure S14.** NOESY spectrum of compound **2**;

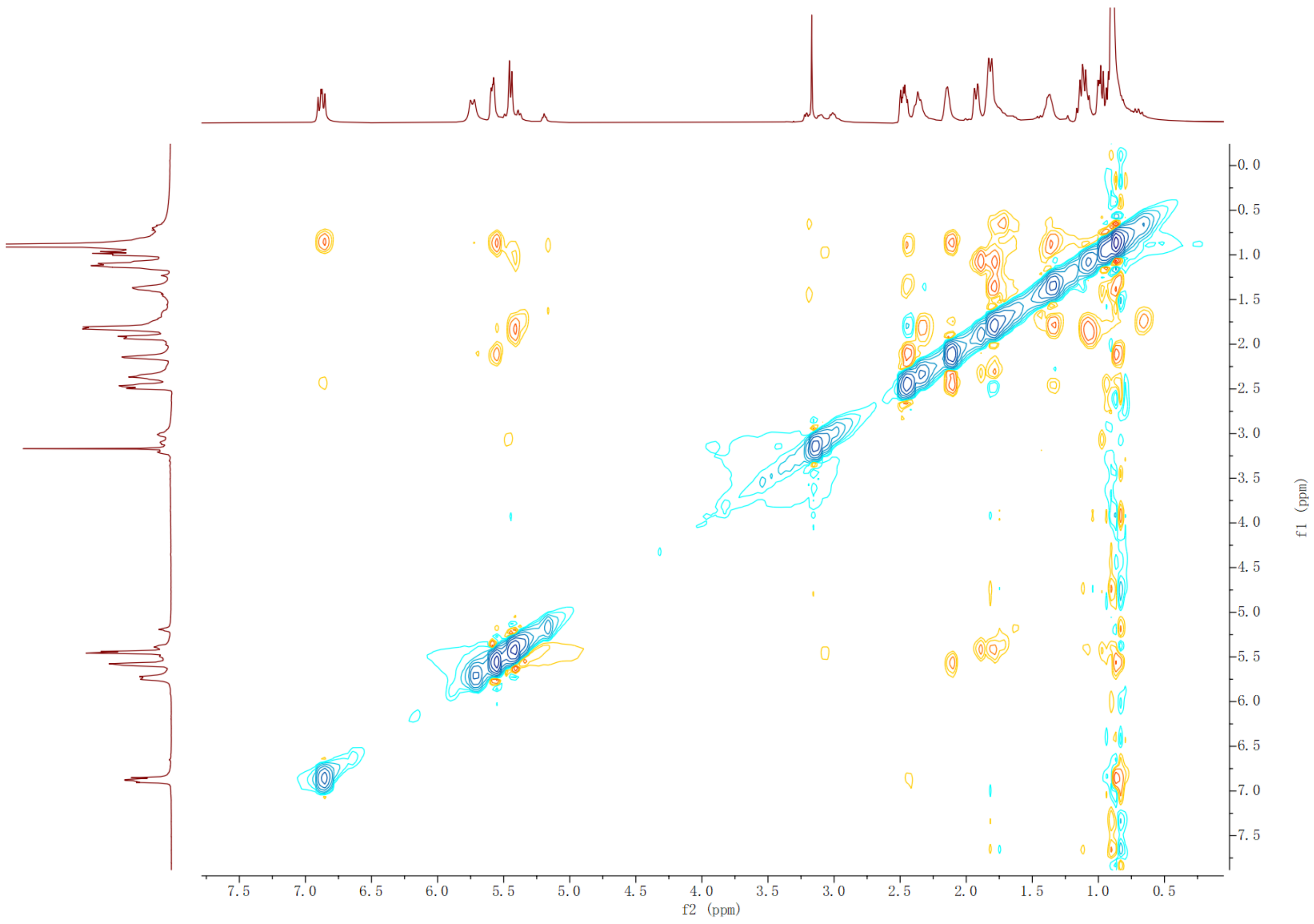

**Figure S15.** HRESI mass spectrum of compound **3**;

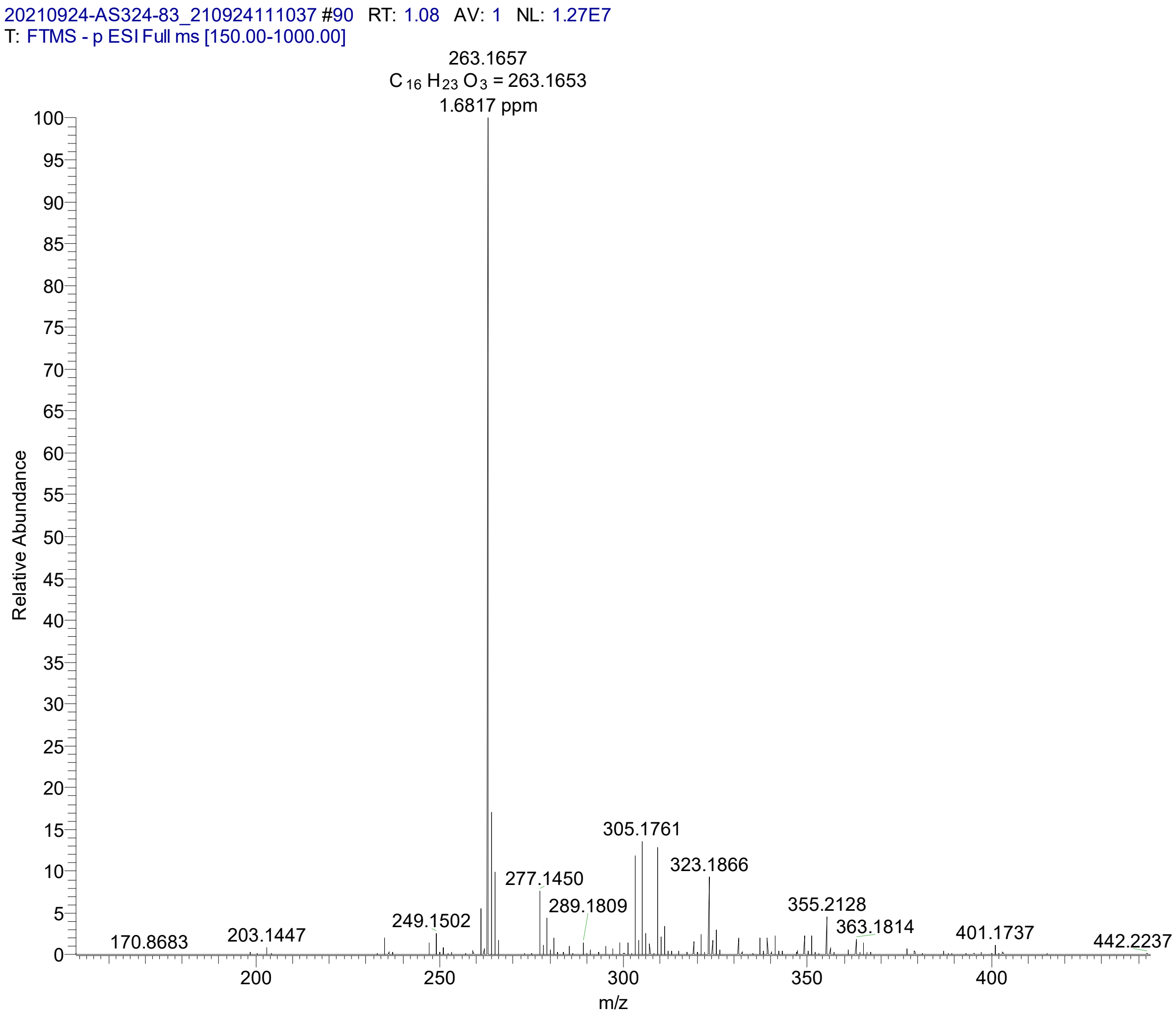

**Figure S16**. $^{1}$H NMR (500 MHz, DMSO-$d_6$) spectrum of compound **3**;

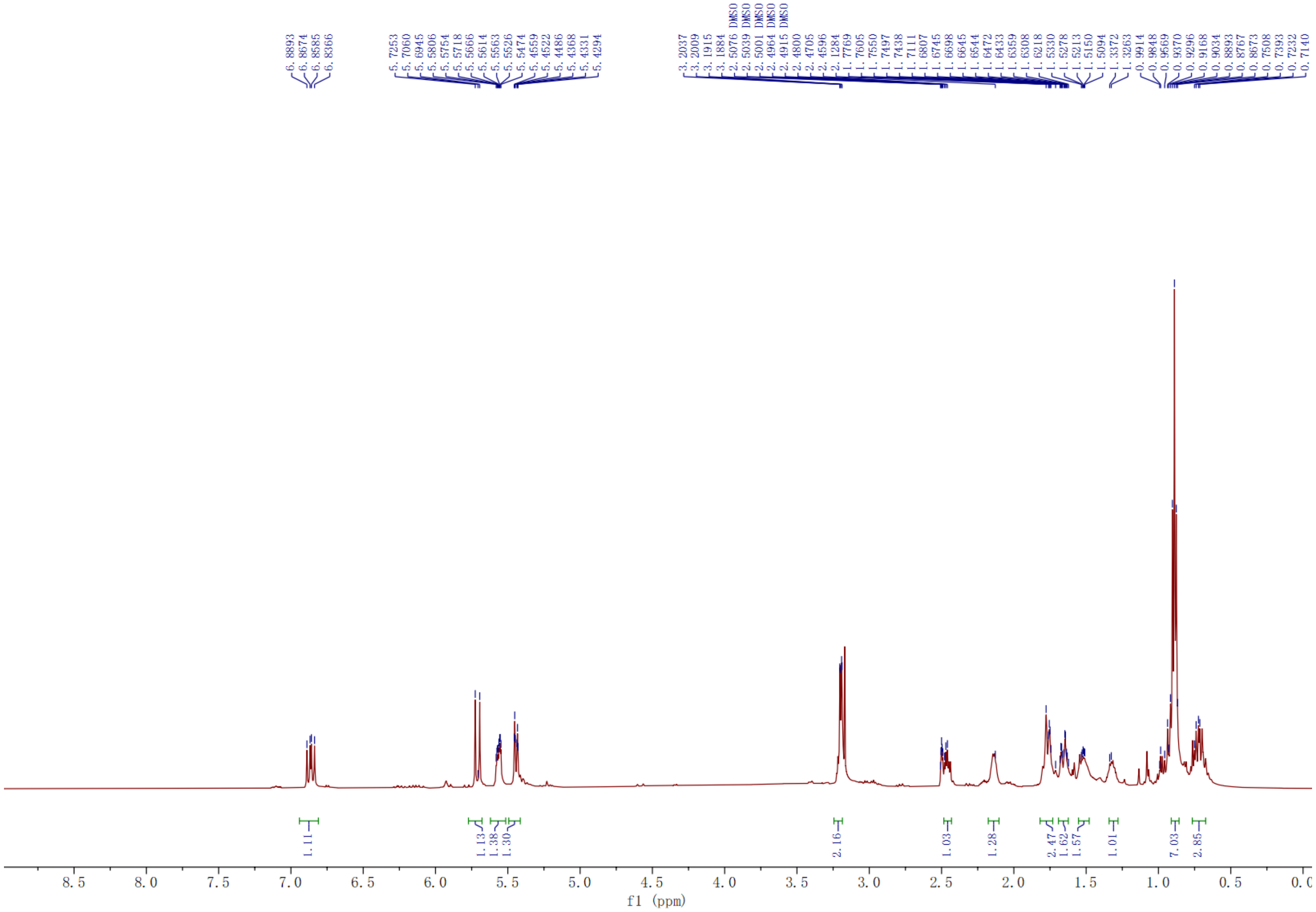

**Figure S17.** $^{13}$C NMR (125 MHz, DMSO-$d_6$) and DEPT spectra of compound **3**;

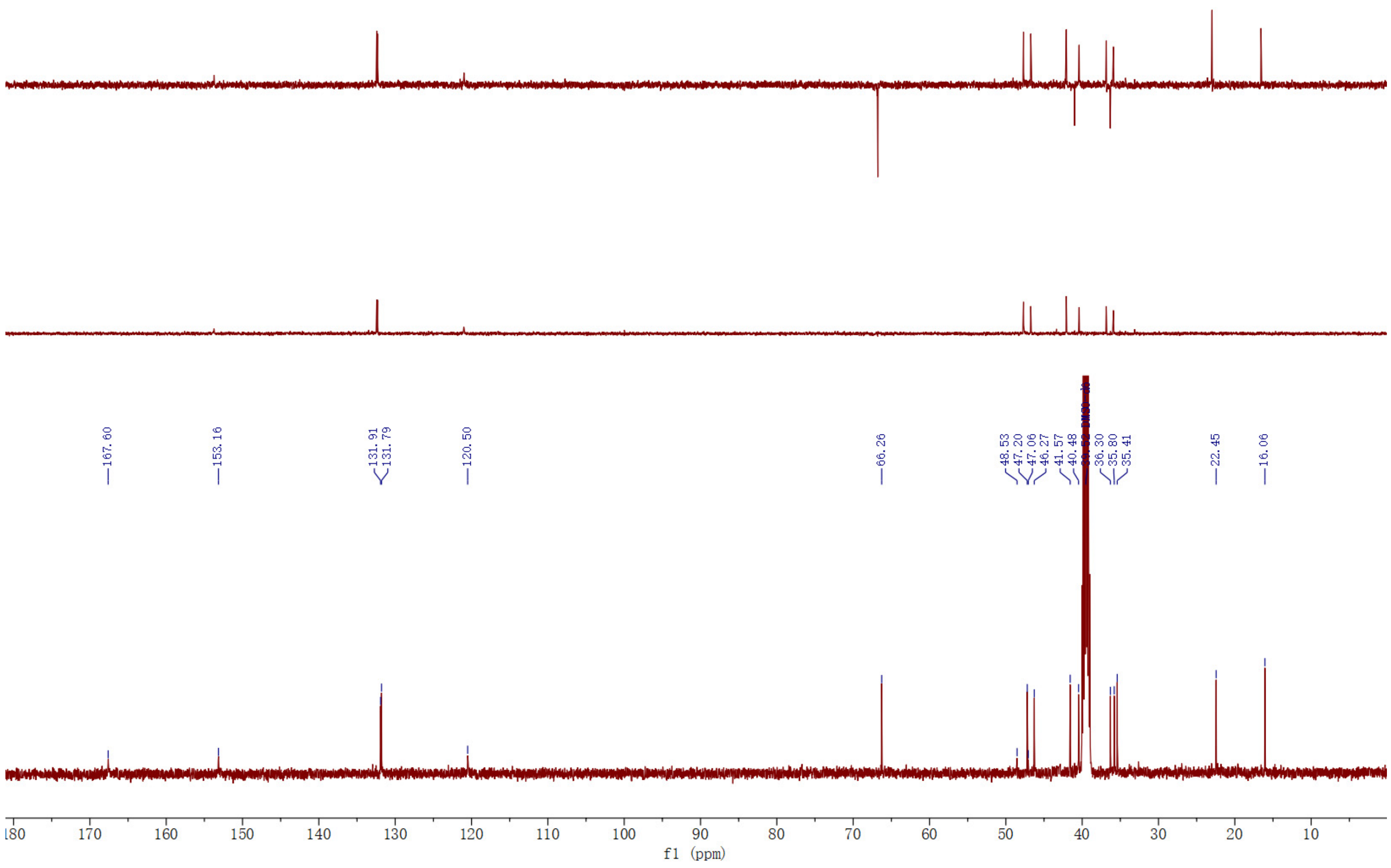

**Figure S18**. COSY spectrum of compound **3**;

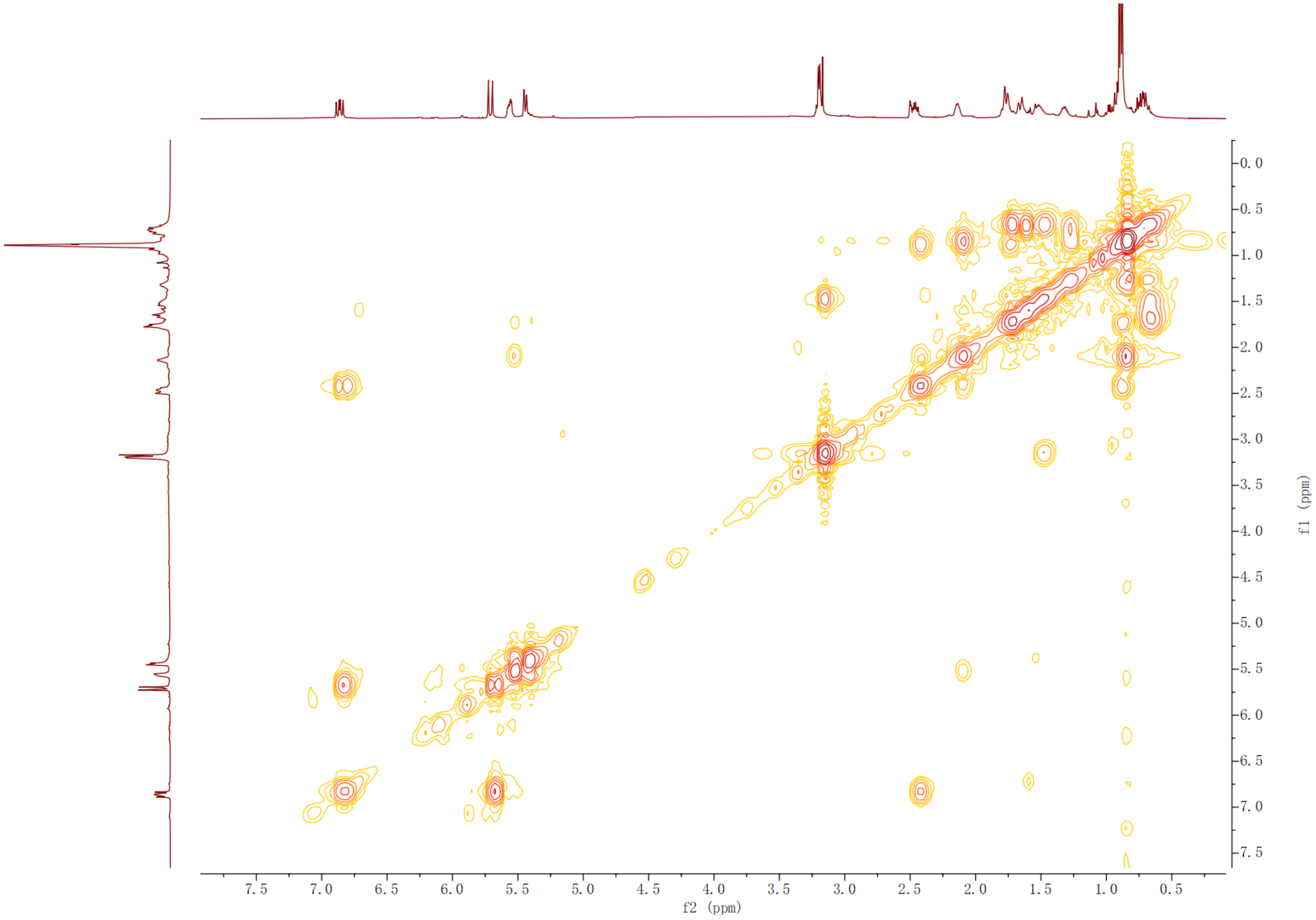

**Figure S19**. HSQC spectrum of compound **3**;

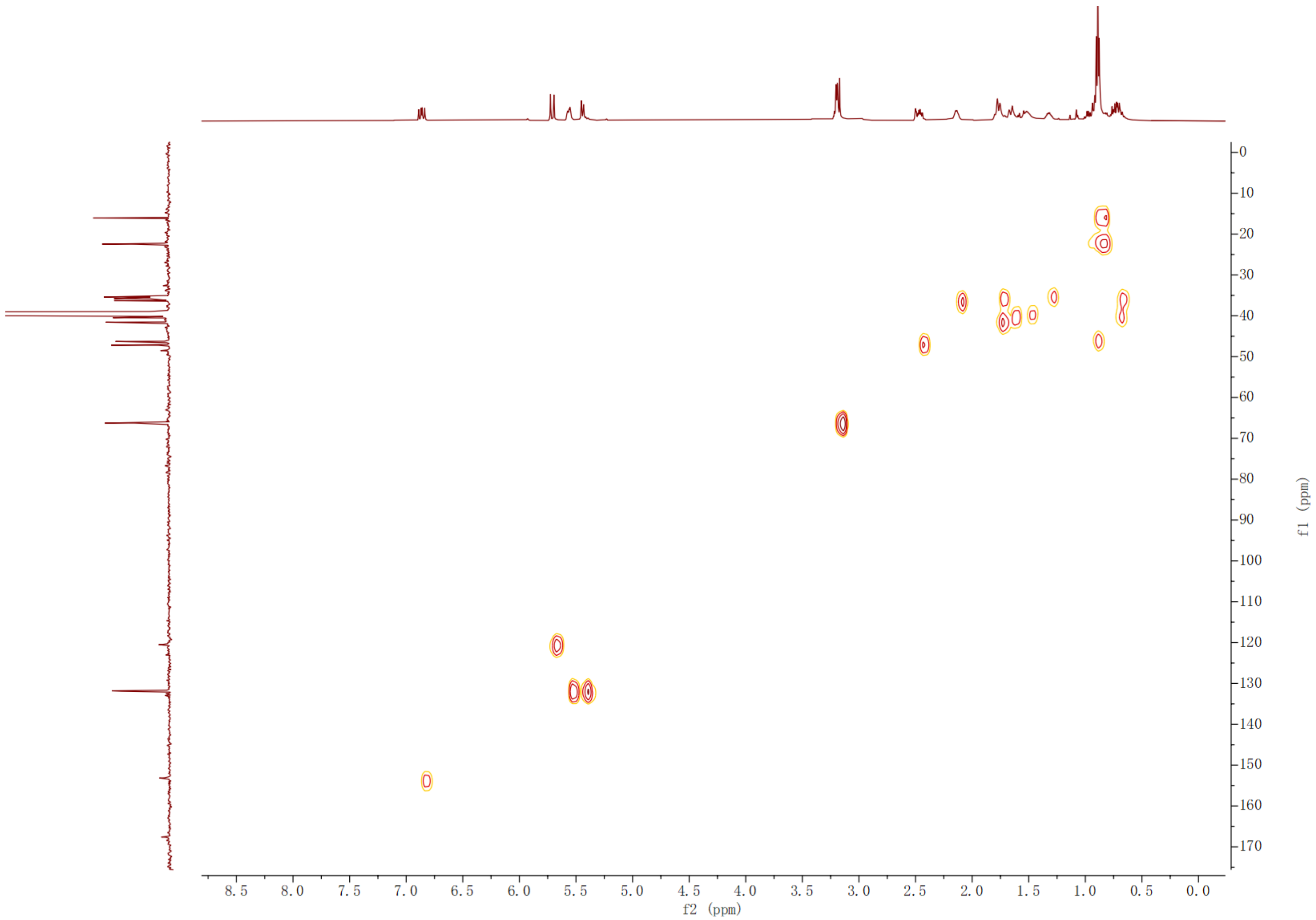

**Figure S20.** HMBC spectrum of compound **3**;

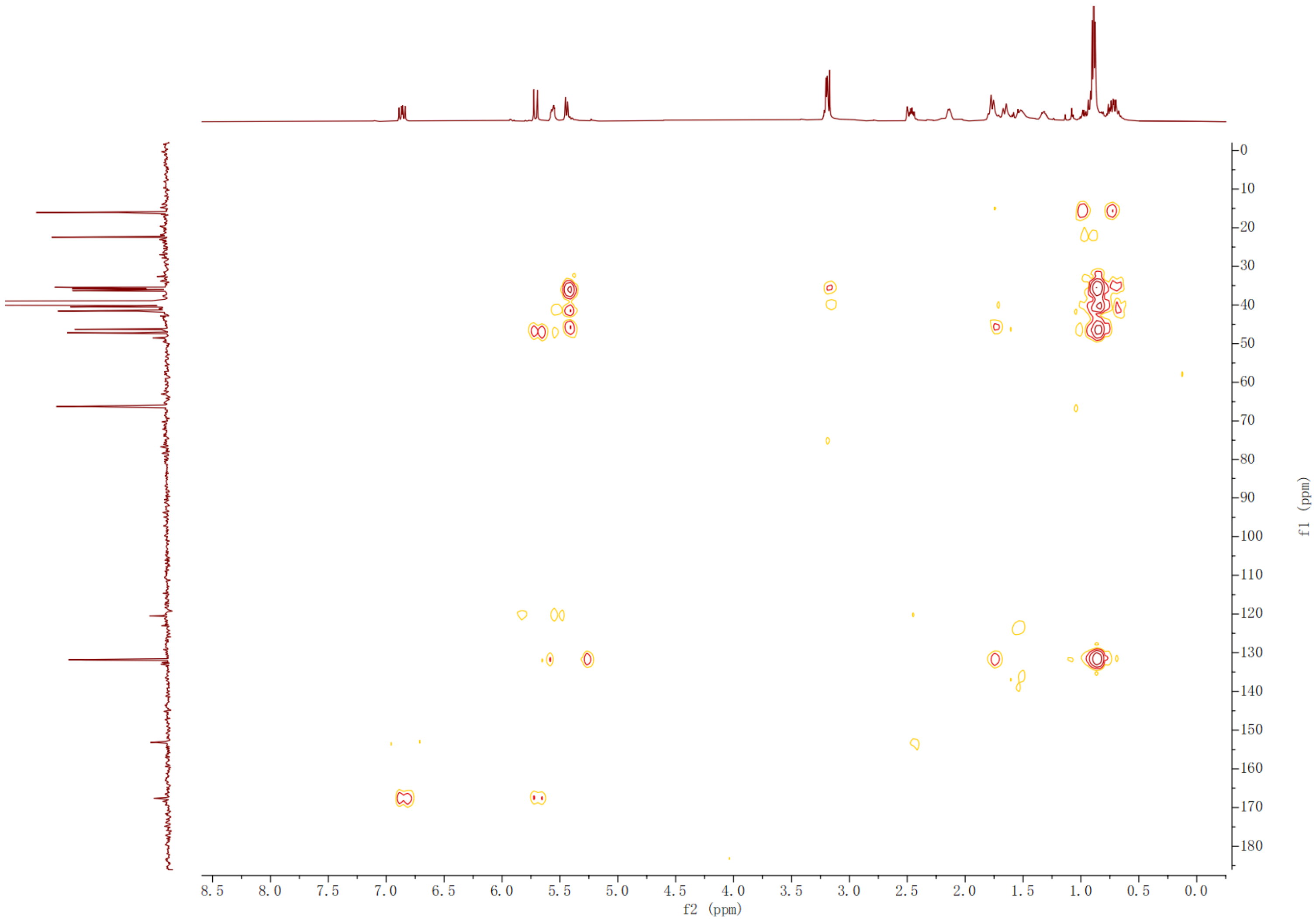

**Figure S21**. NOESY spectrum of compound **3**;

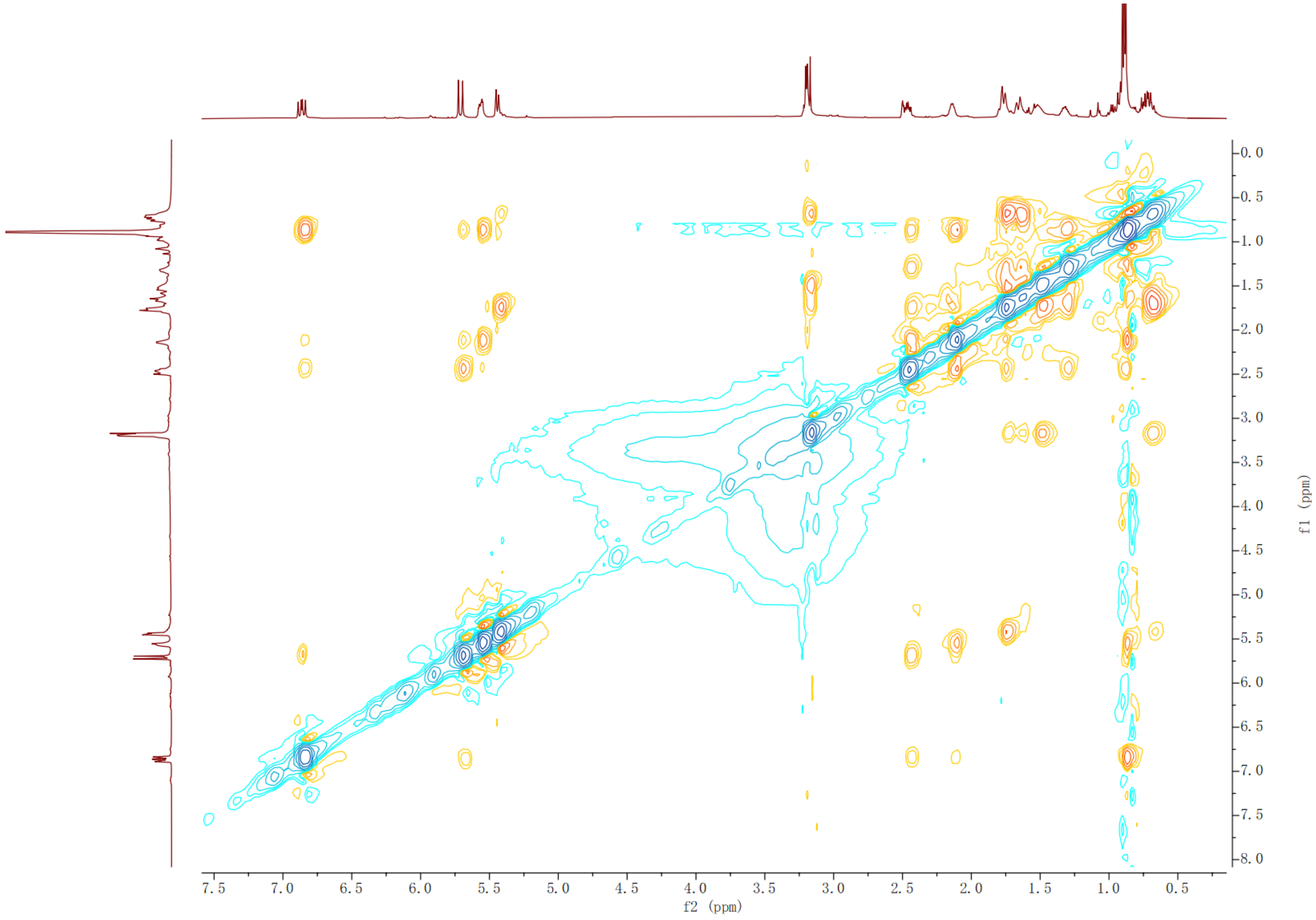

**Figure S22**. HRESI mass spectrum of compound **4**;

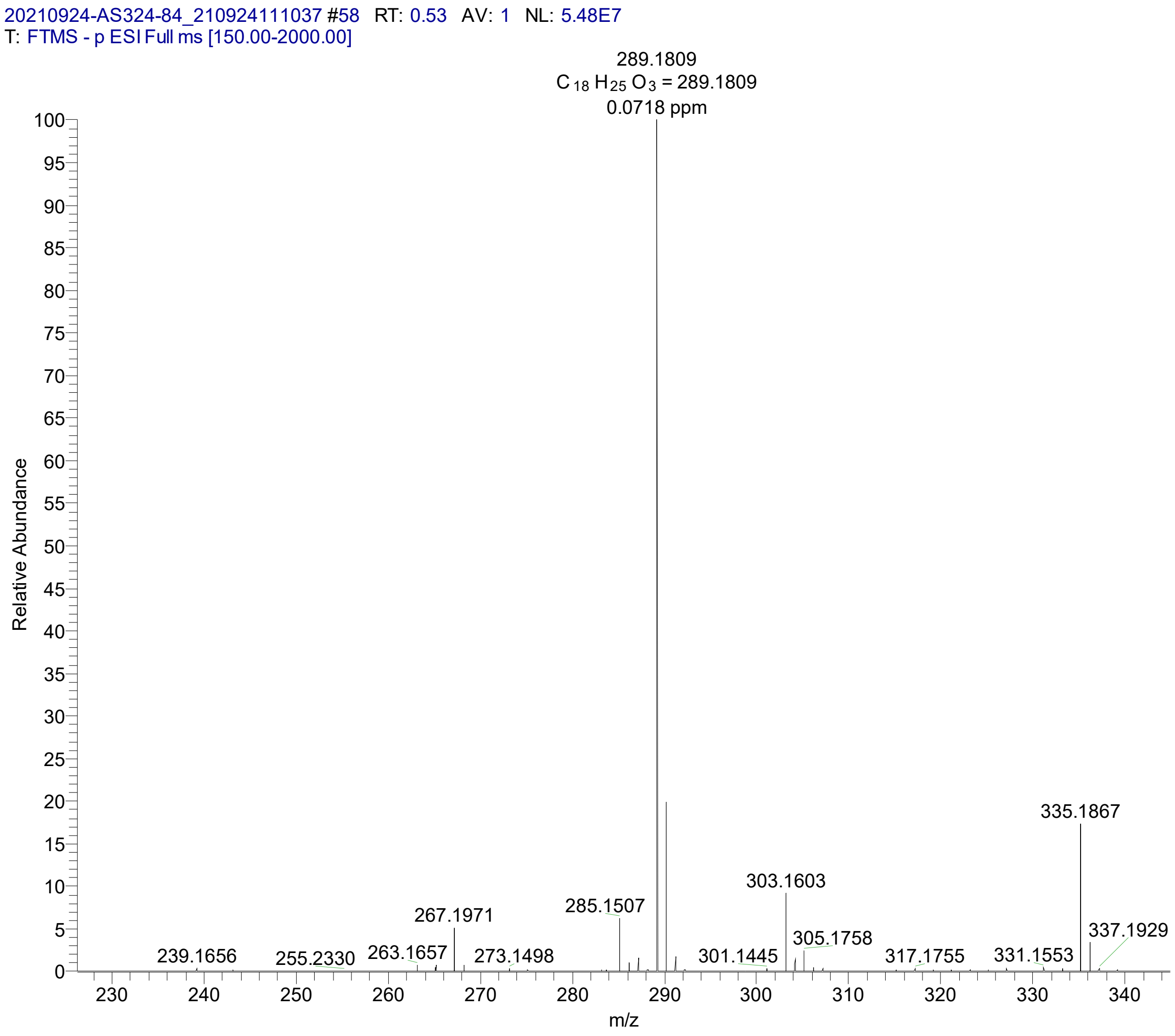

**Figure S23**. $^{1}$H NMR (500 MHz, DMSO-$d_6$) spectrum of compound **4**;

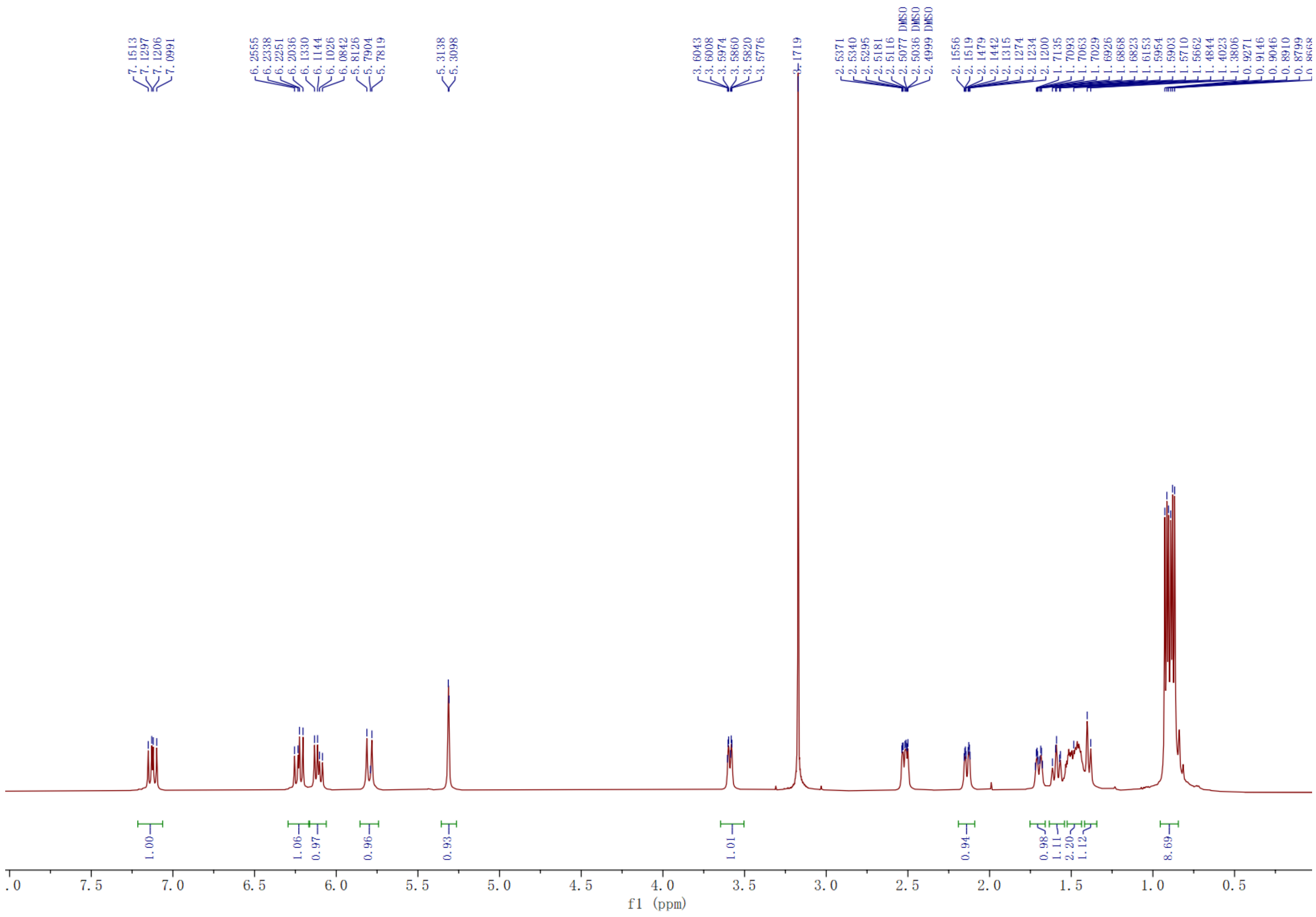

**Figure S24**. $^{13}$C NMR (125 MHz, DMSO-$d_6$) and DEPT spectra of compound **4**;

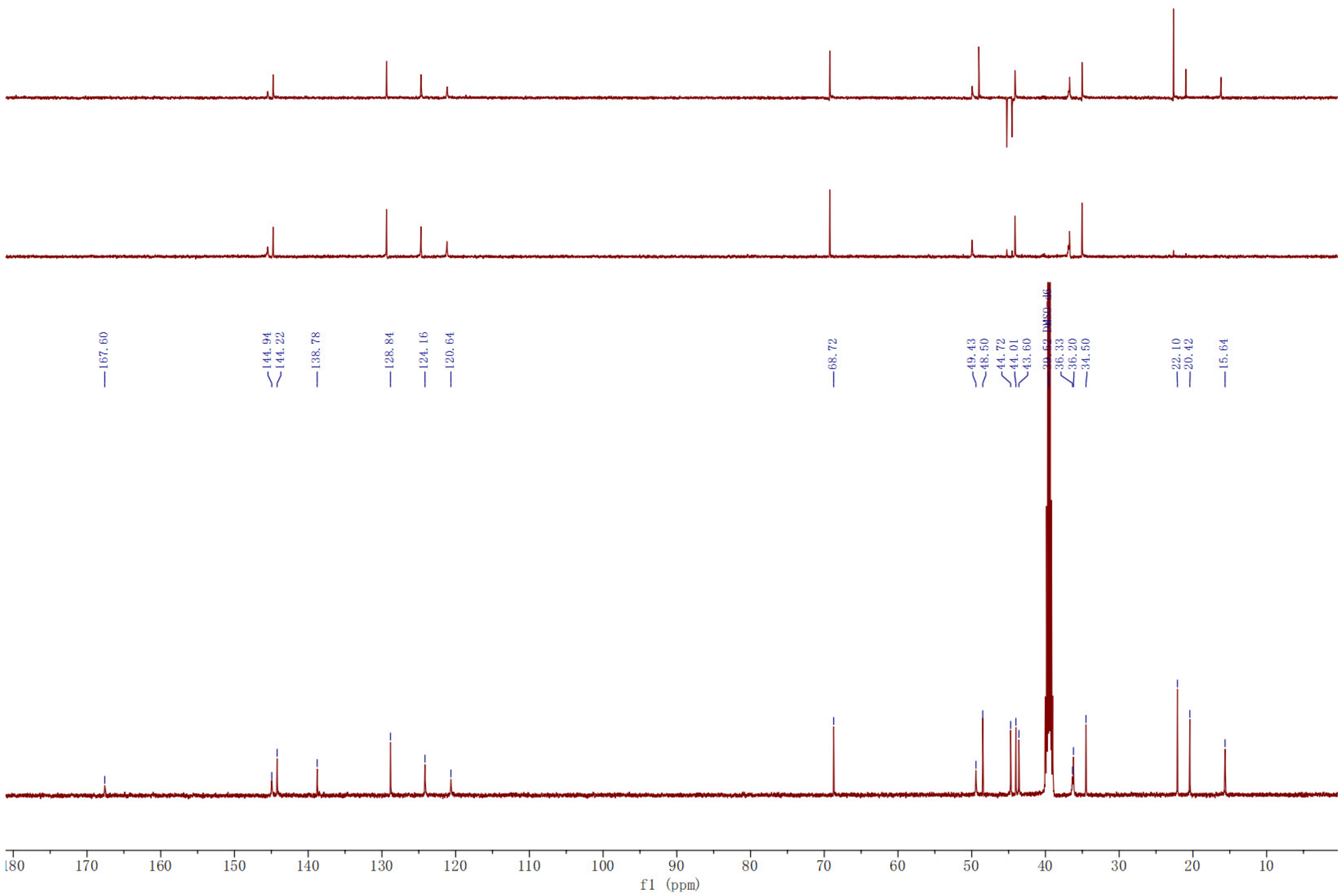

**Figure S25**. COSY spectrum of compound **4**;

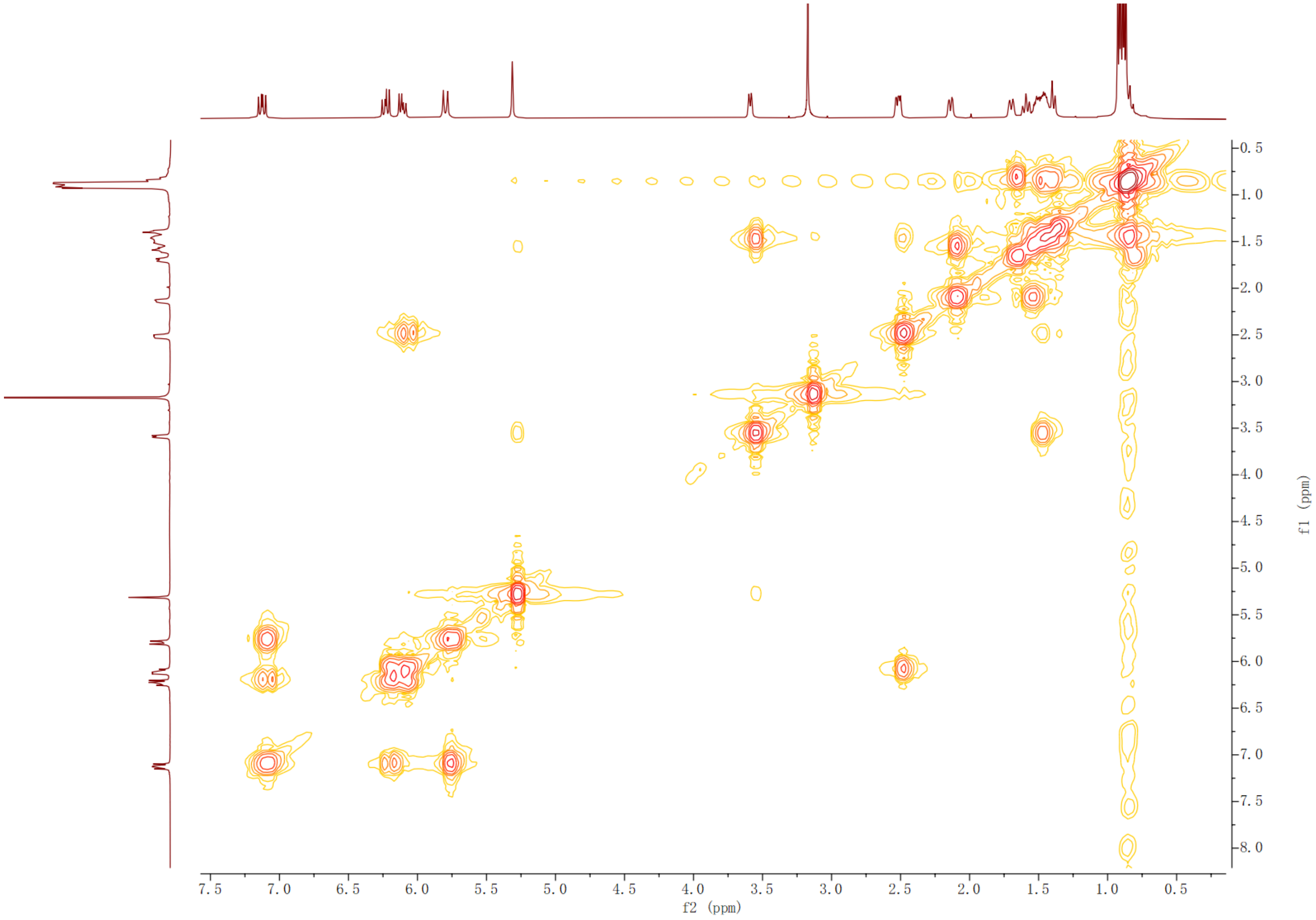

**Figure S26.** HSQC spectrum of compound **4**;

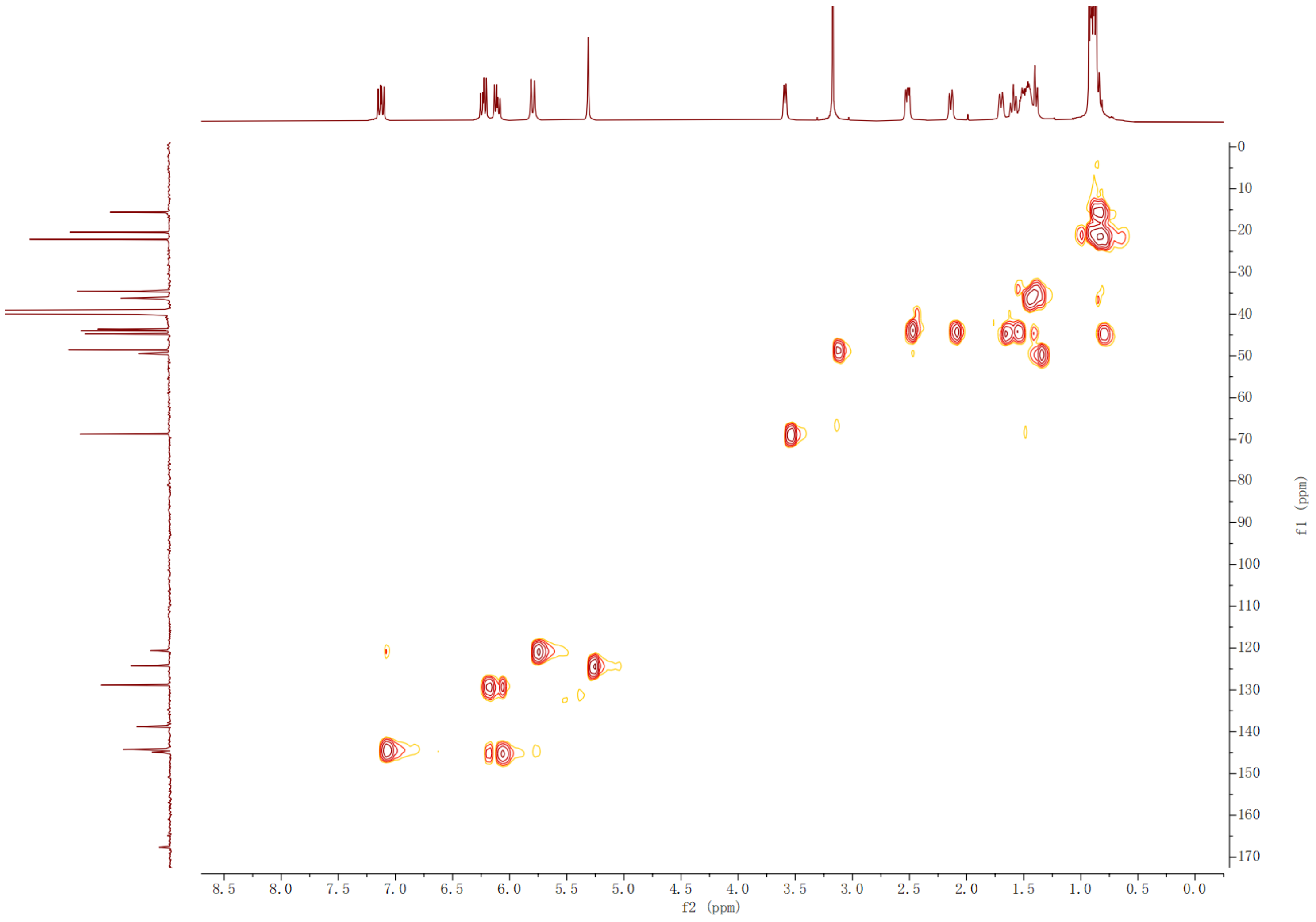

**Figure S27**. HMBC spectrum of compound **4**;

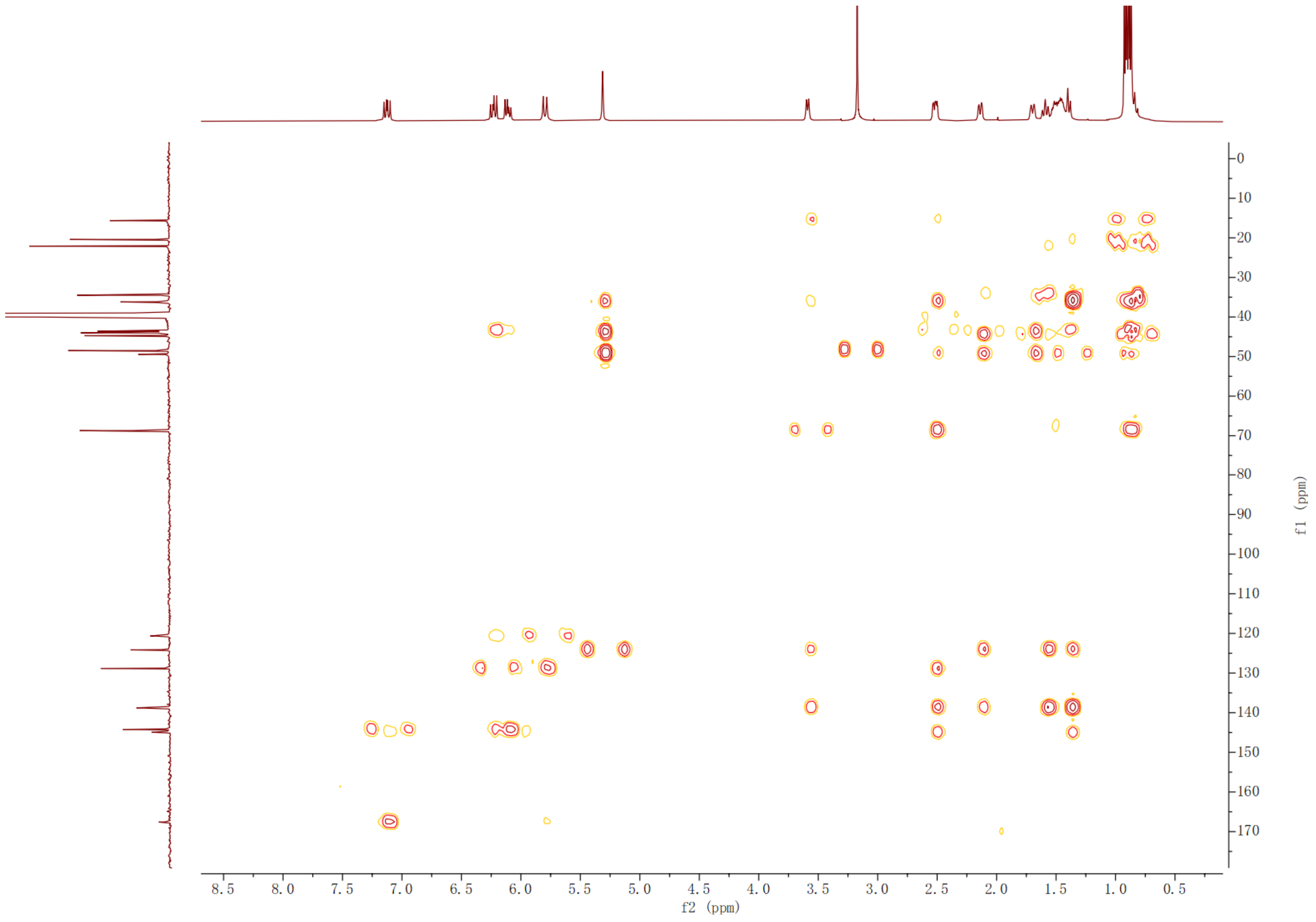

**Figure S28.** NOESY spectrum of compound **4**;

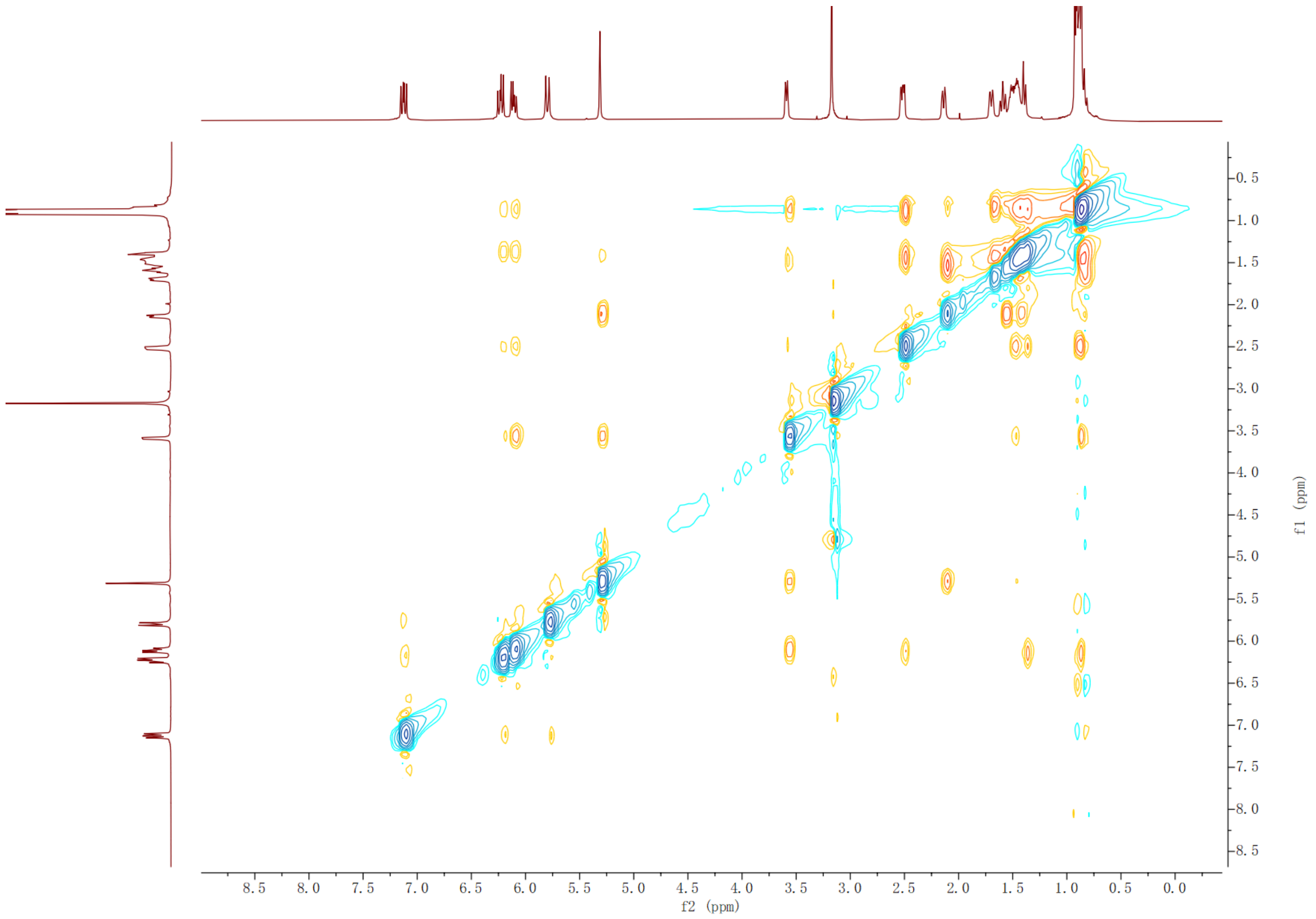

**Figure S29**. HRESI mass spectrum of compound **5**;

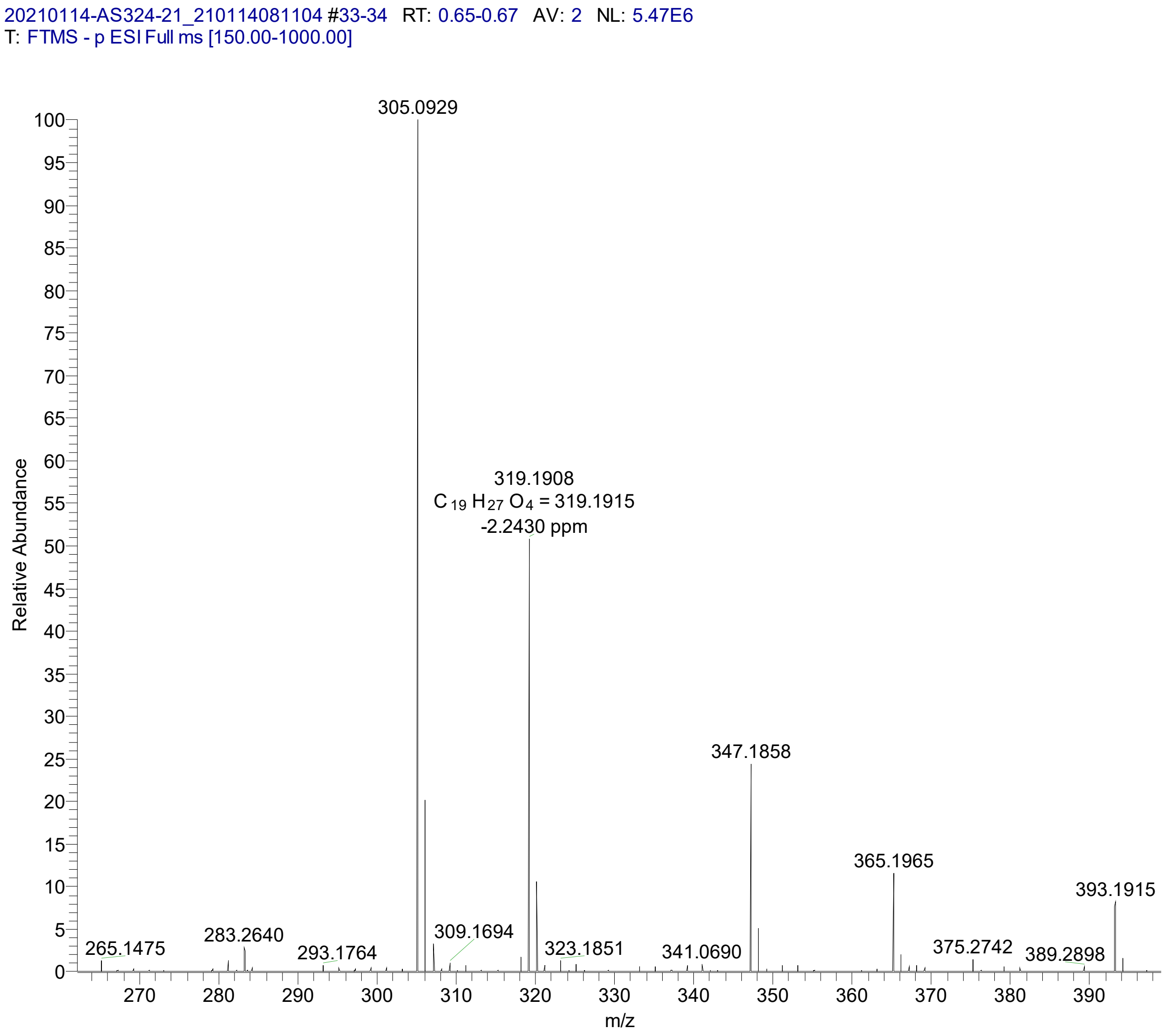

**Figure S30**. $^{1}$H NMR (500 MHz, DMSO-$d_6$) spectrum of compound **5**;

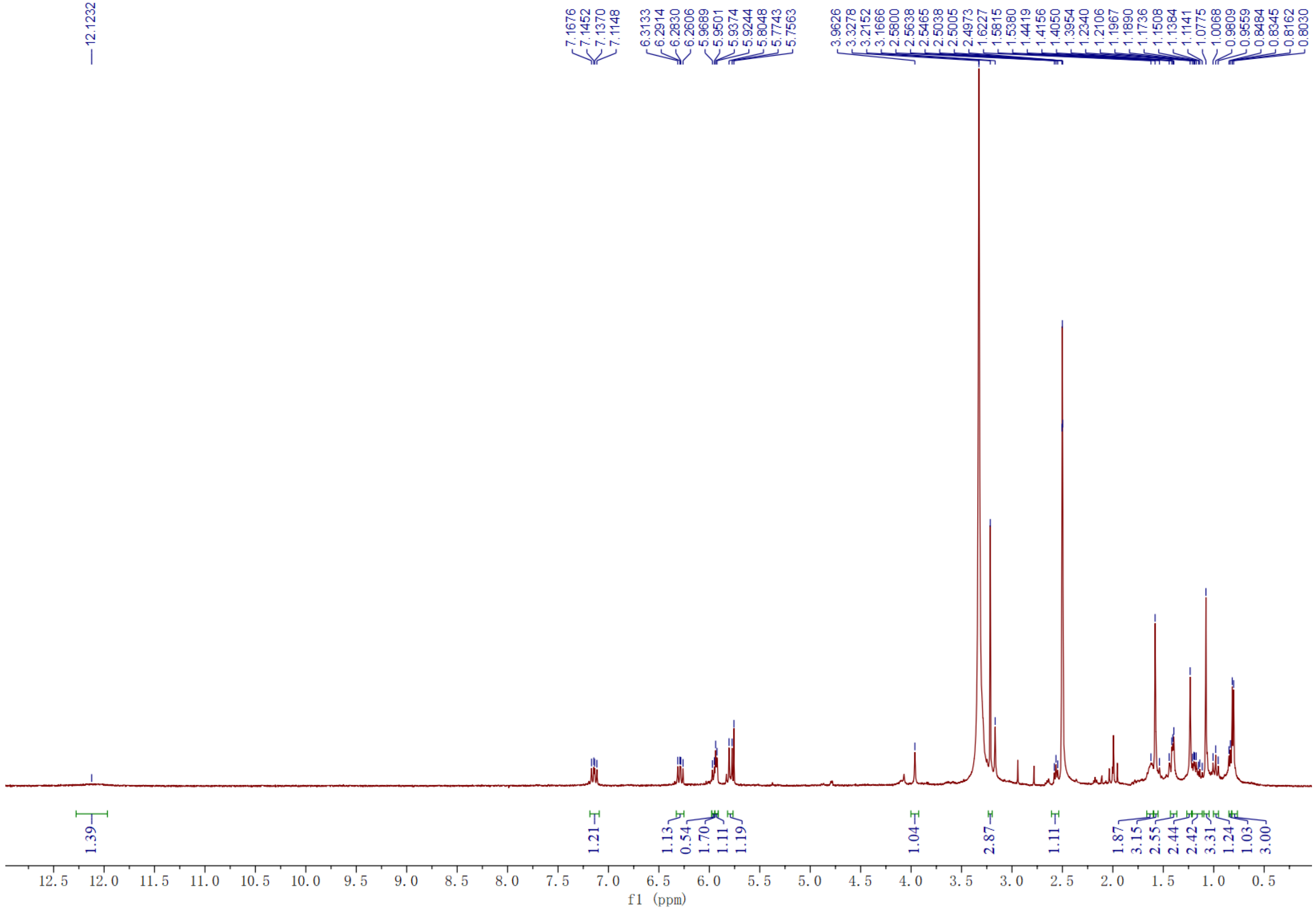

**Figure S31**. $^{13}$C NMR (125 MHz, DMSO-$d_6$) and DEPT spectra of compound **5**;

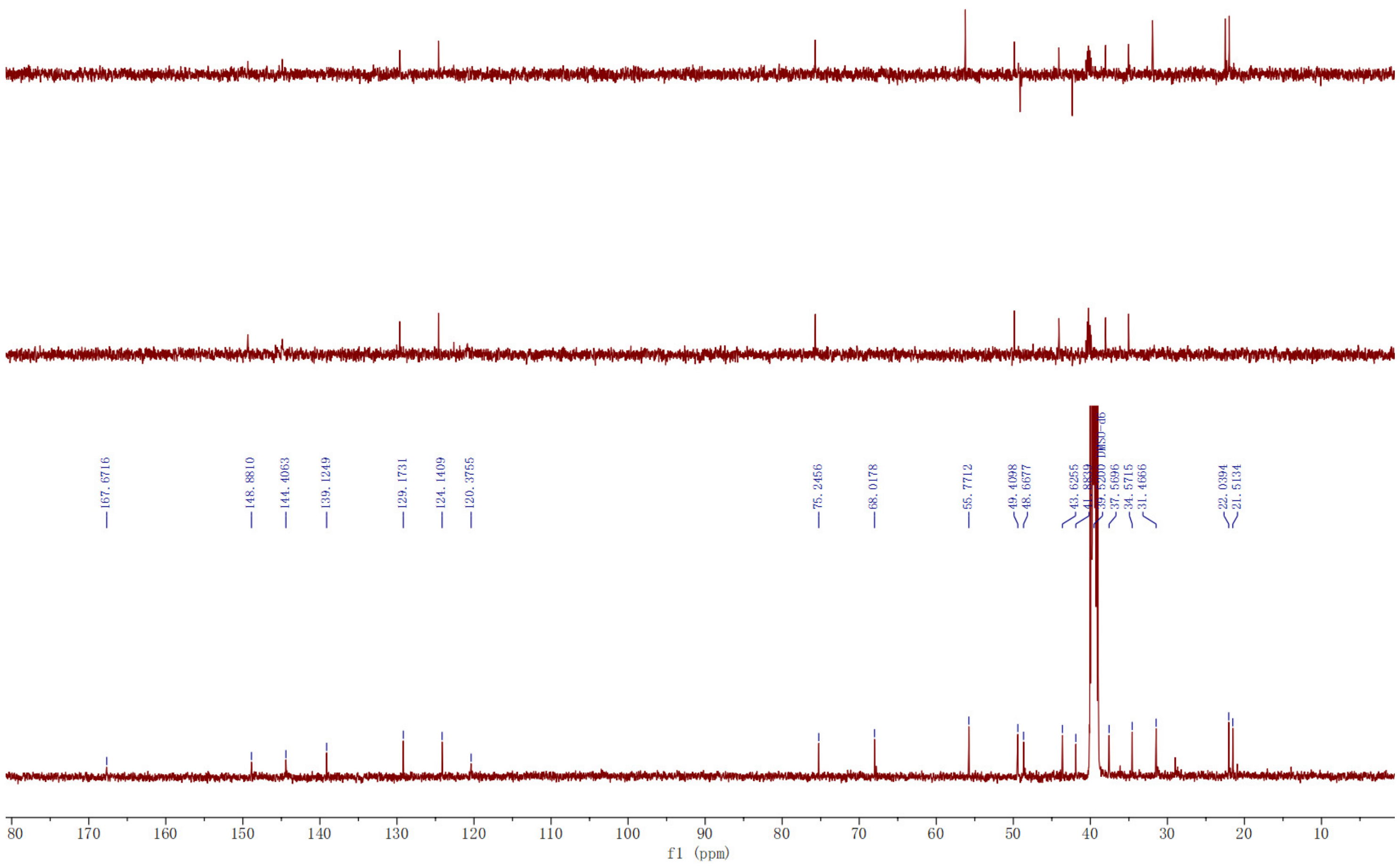

**Figure S32**. COSY spectrum of compound **5**;

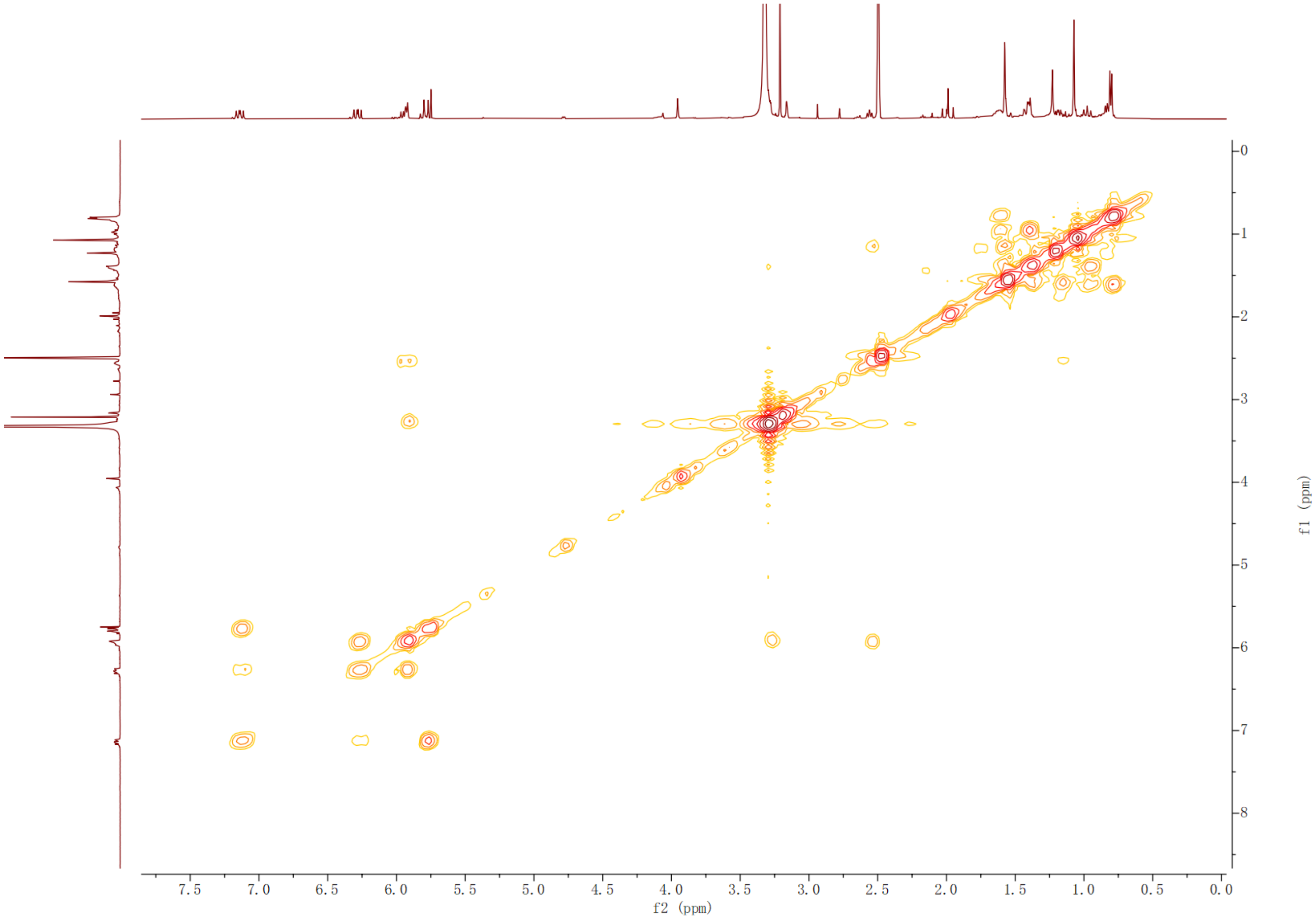

**Figure S33.** HSQC spectrum of compound **5**;

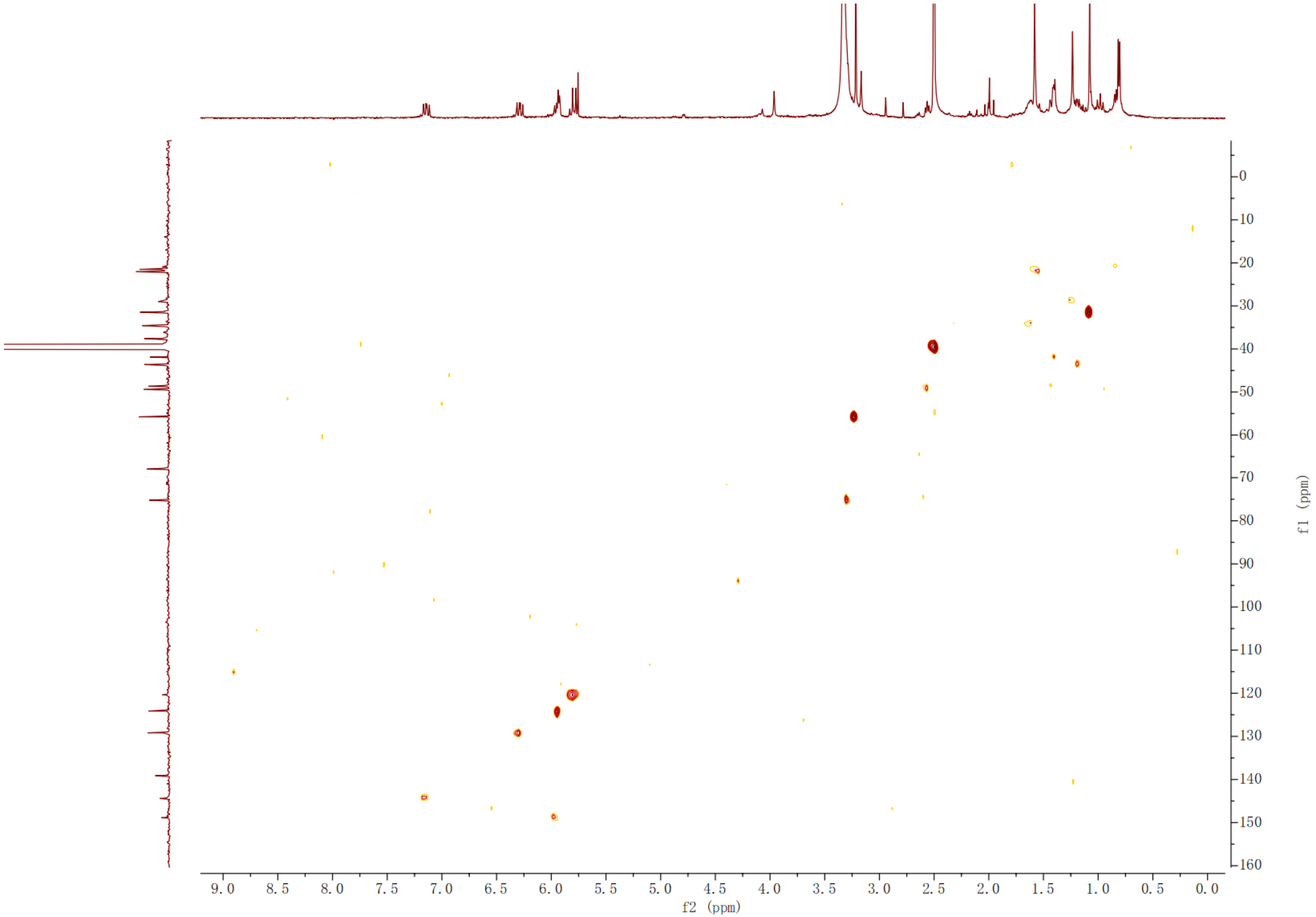

**Figure S34**. HMBC spectrum of compound **5**;

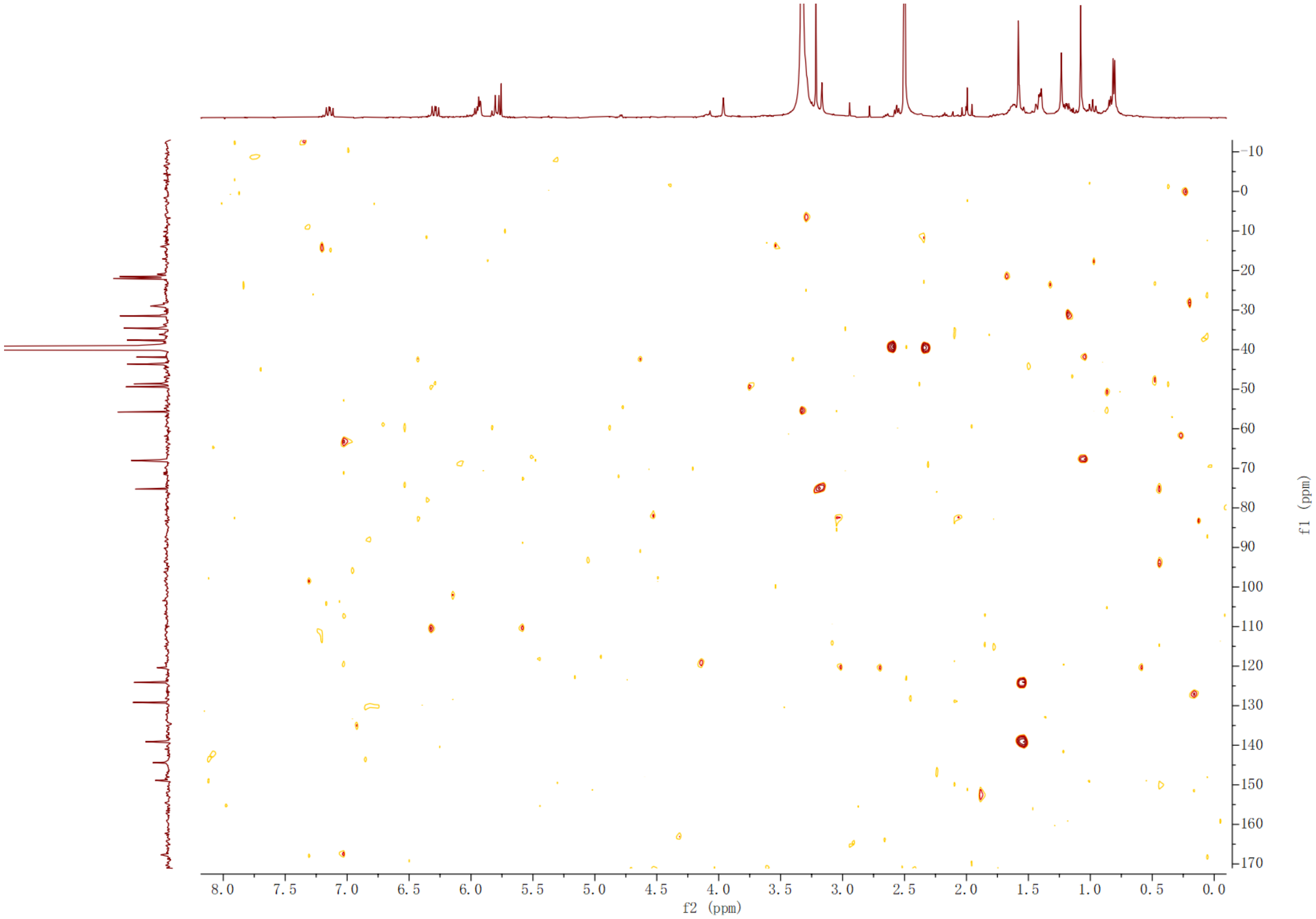

**Figure S35**. NOESY spectrum of compound **5**;

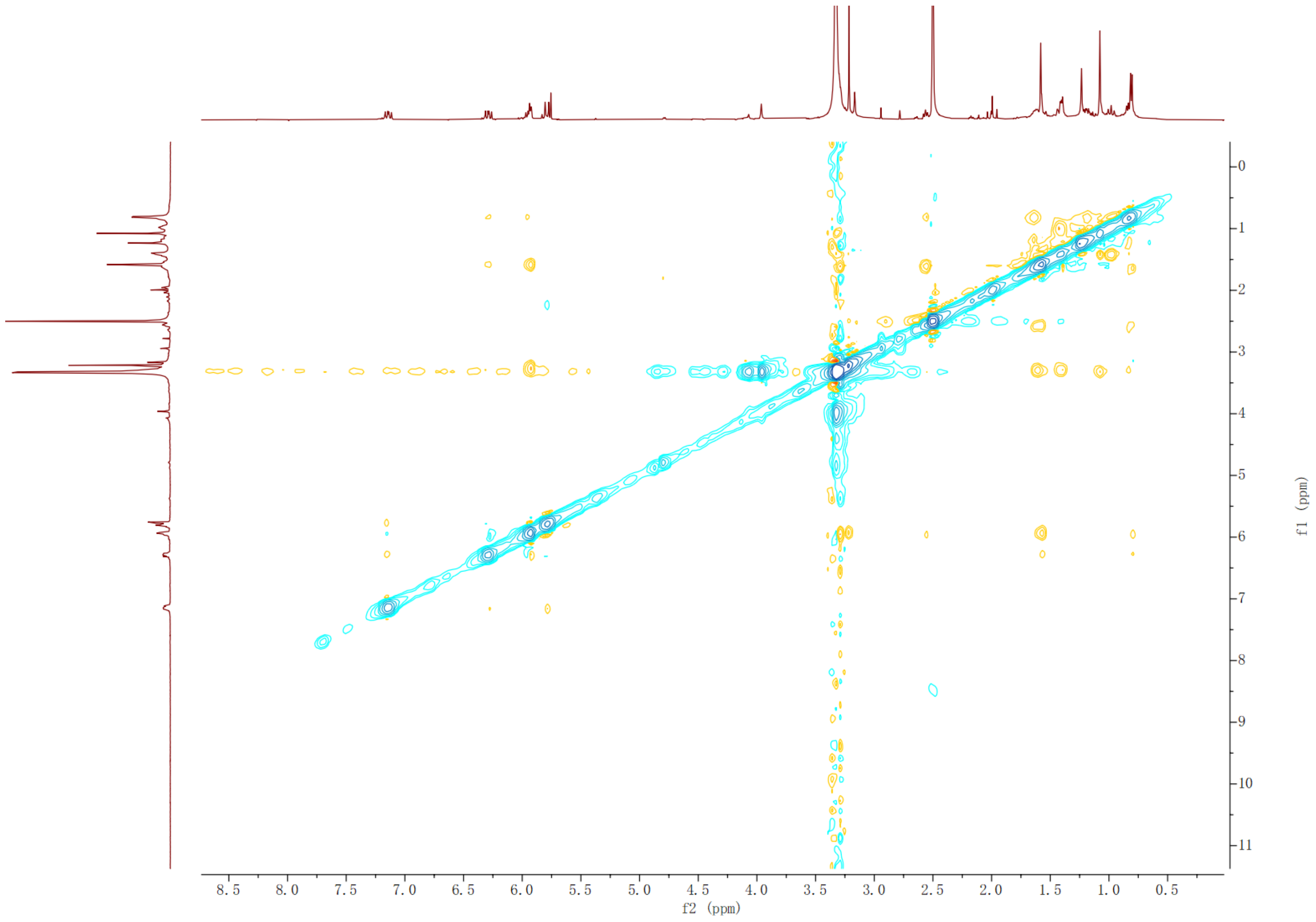

**Figure S36**. HRESI mass spectrum of compound **6**;

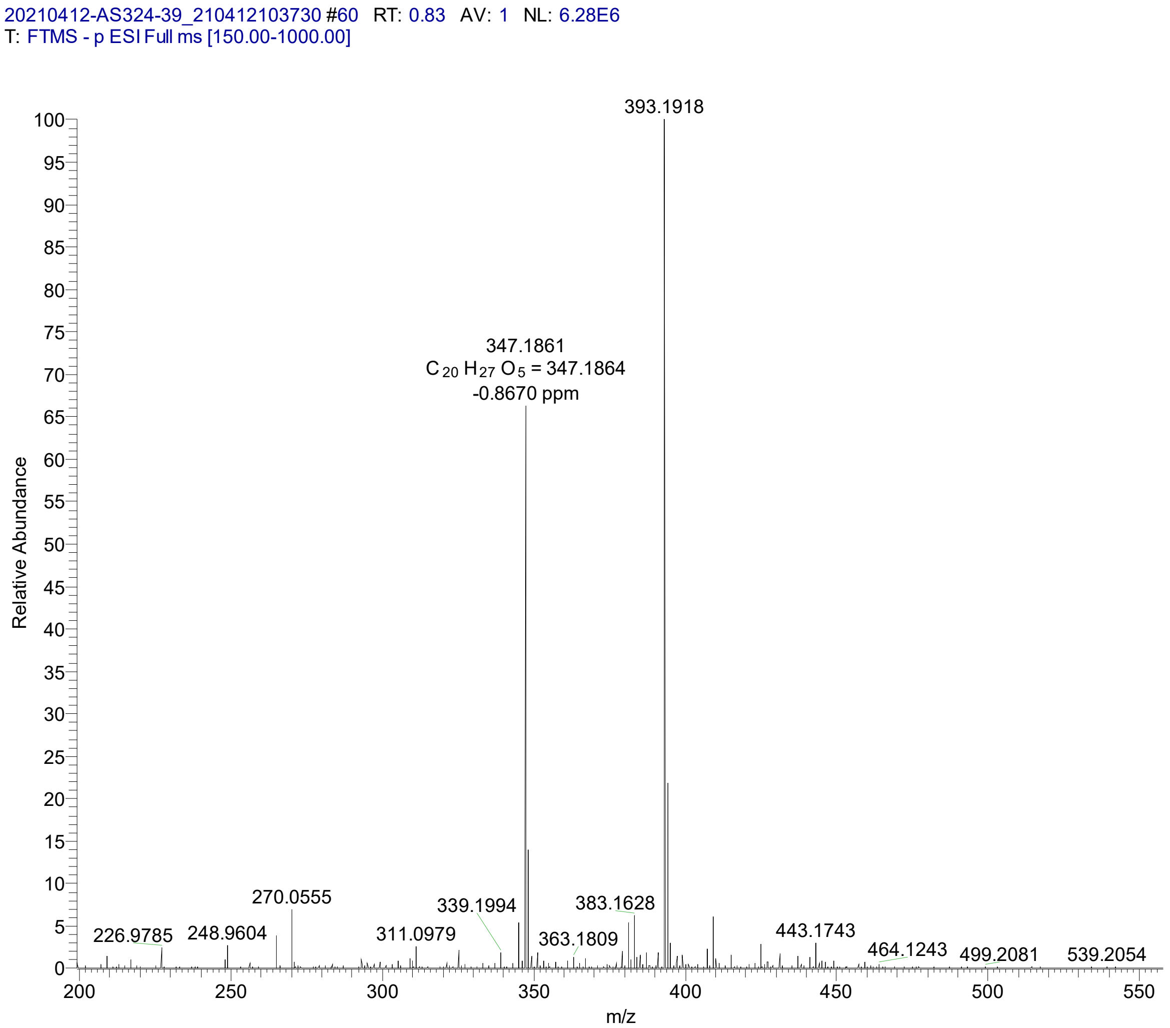

**Figure S37**. $^{1}$H NMR (500 MHz, DMSO-$d_6$) spectrum of compound **6**;

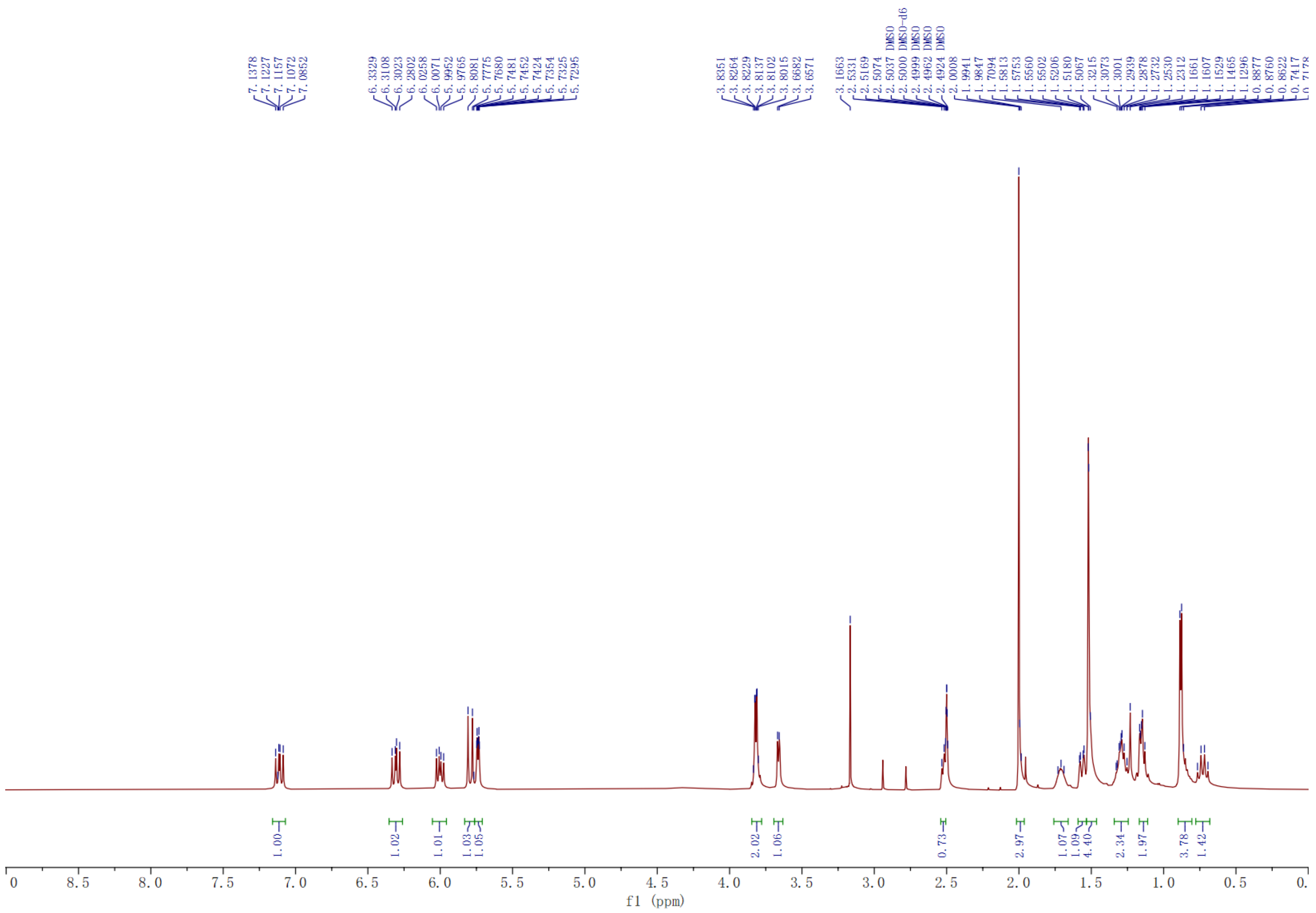

**Figure S38**. $^{13}$C NMR (125 MHz, DMSO-$d_6$) and DEPT spectra of compound **6**;

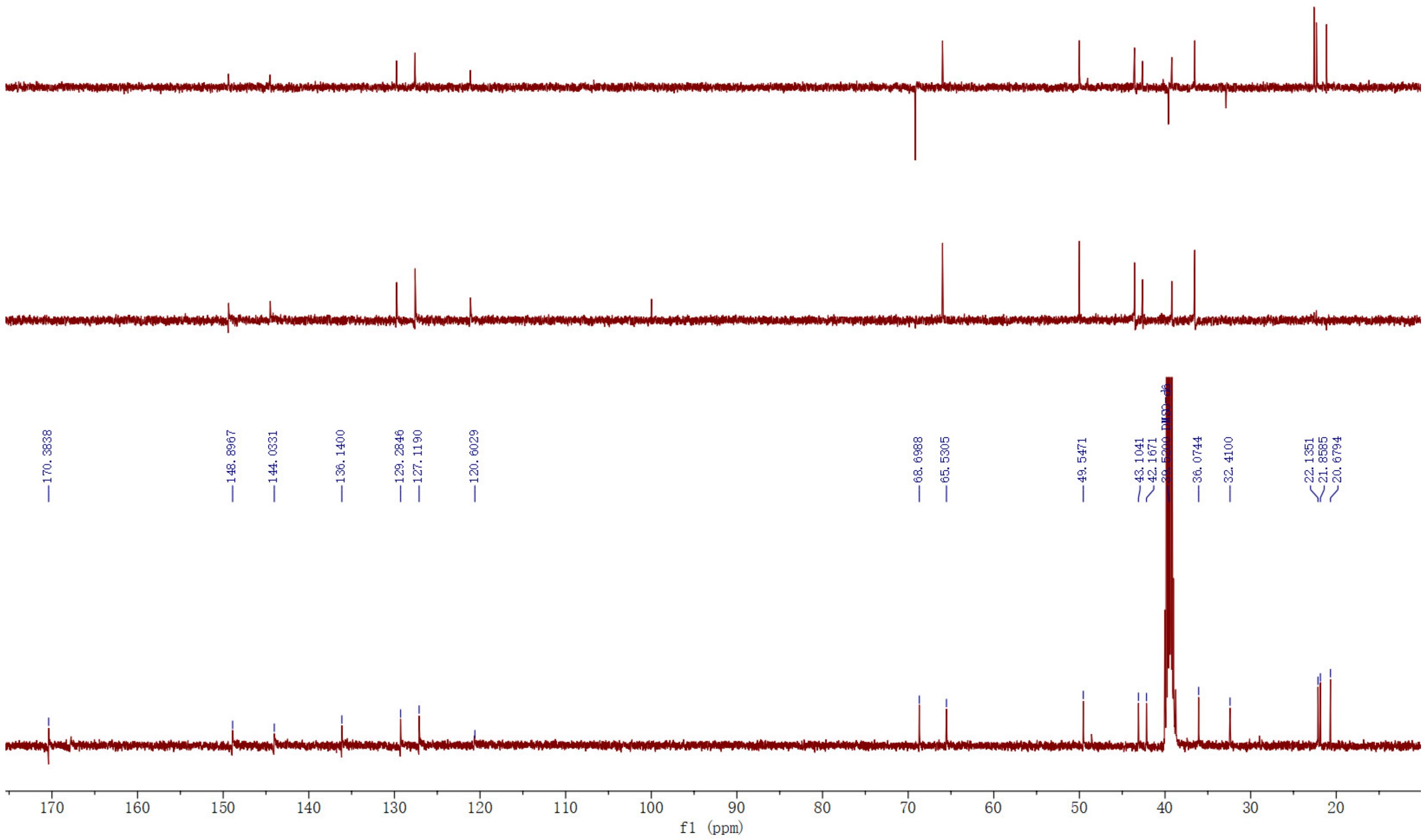

**Figure S39**. COSY spectrum of compound **6**;

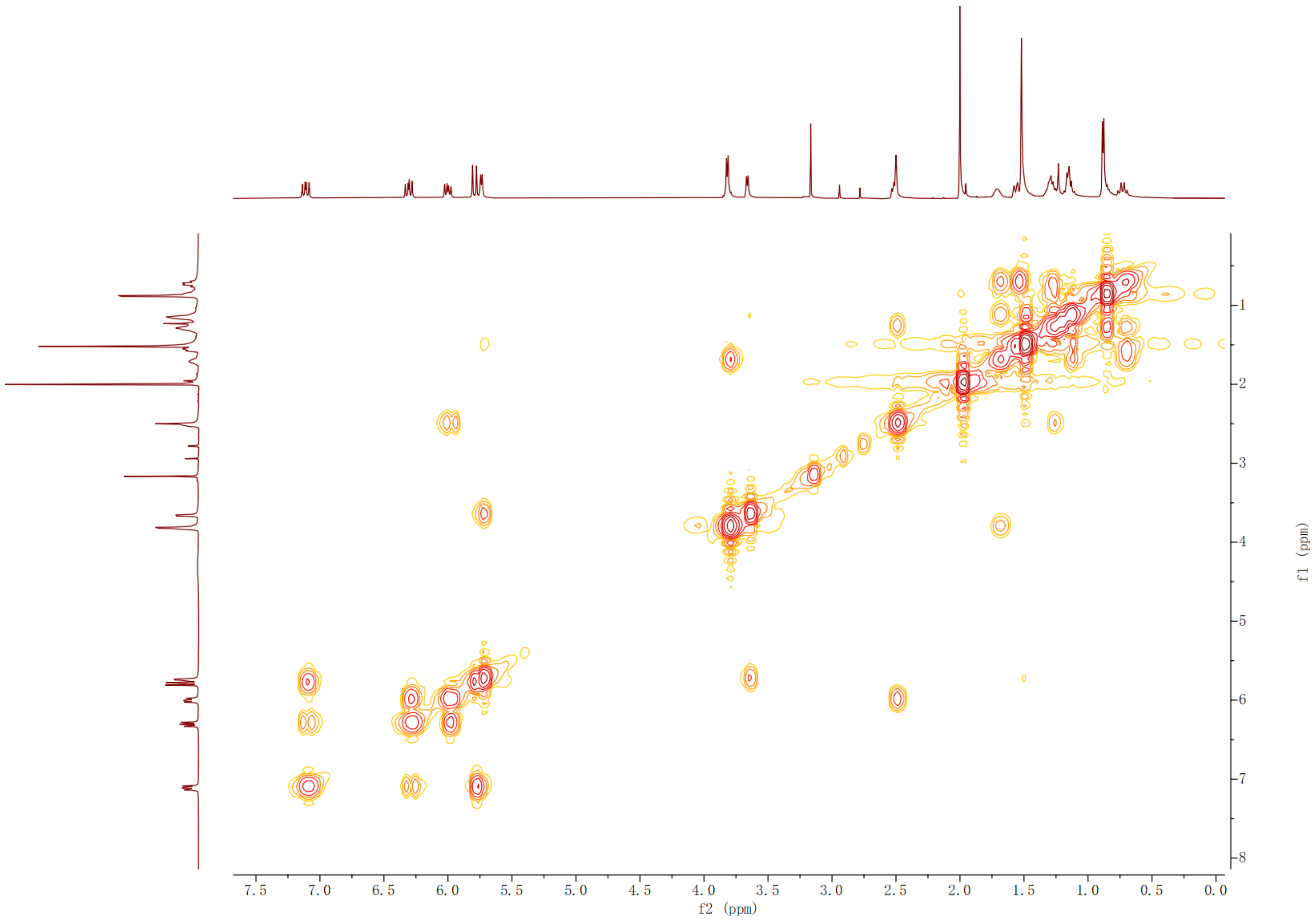

**Figure S40.** HSQC spectrum of compound **6**;

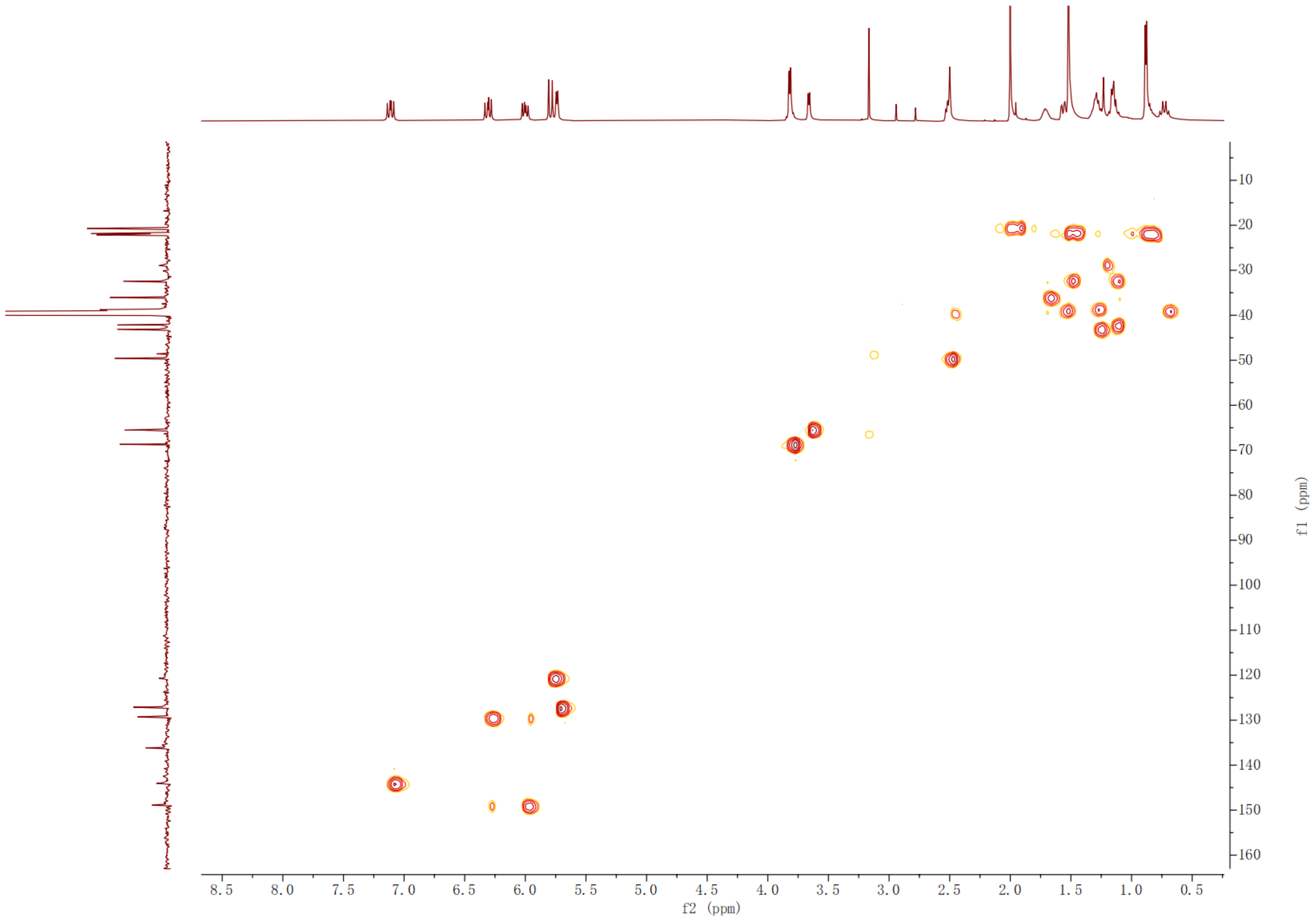

**Figure S41**. HMBC spectrum of compound **6**;

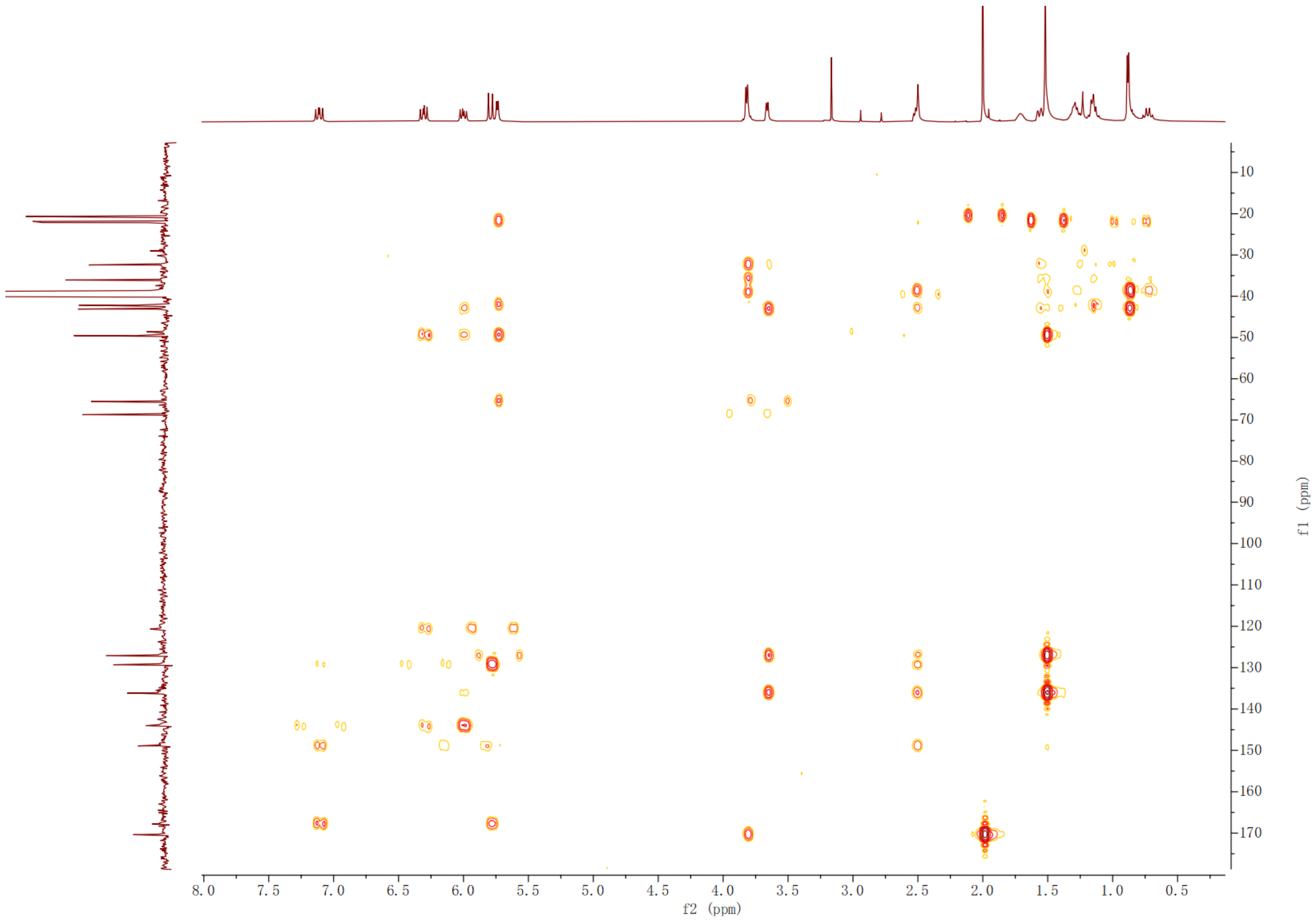

**Figure S42**. NOESY spectrum of compound **6;**

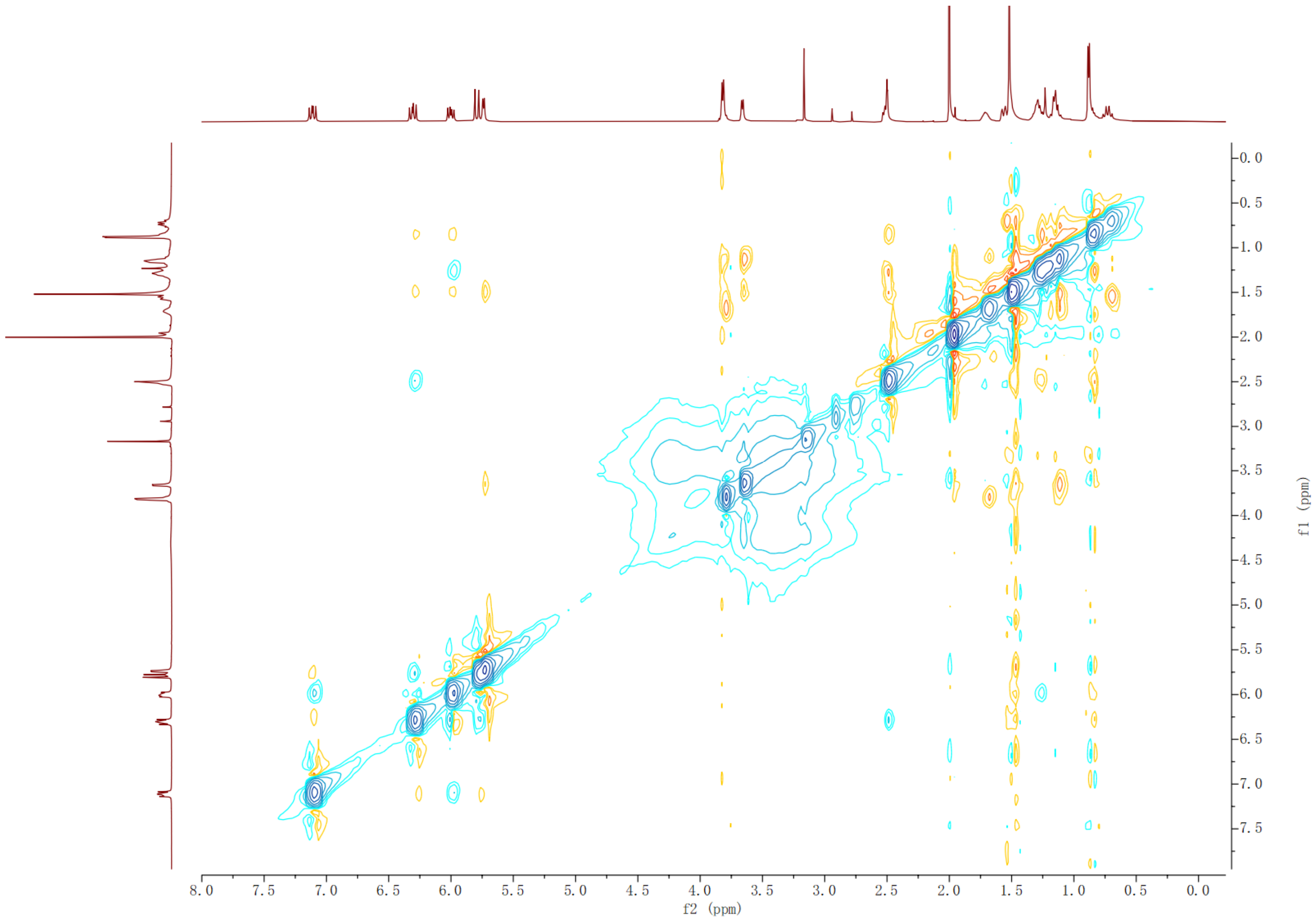

**Figure S43**. HRESI mass spectrum of compound **7**;

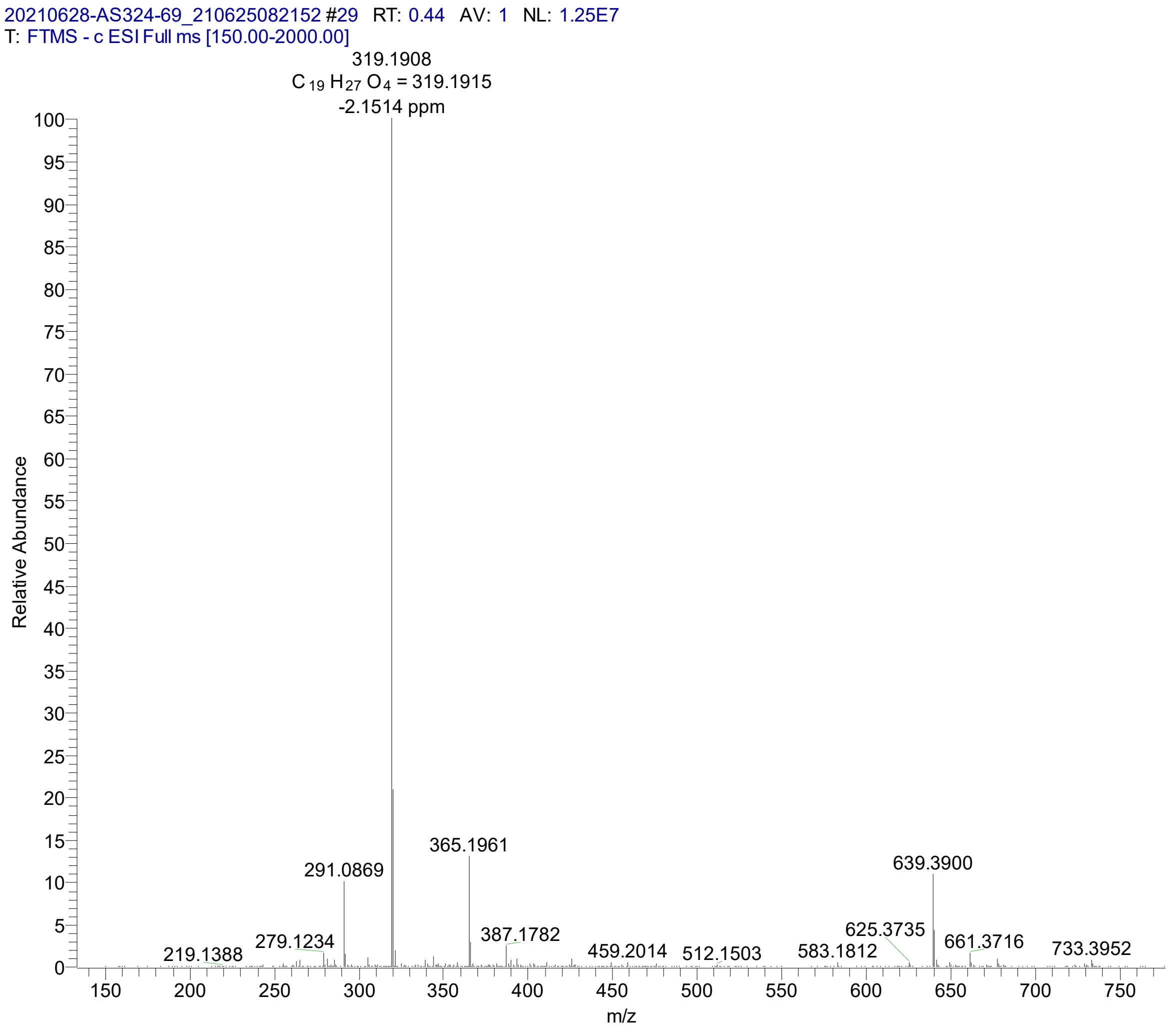

**Figure S44**. $^{1}$H NMR (500 MHz, DMSO-$d_6$) spectrum of compound **7**;

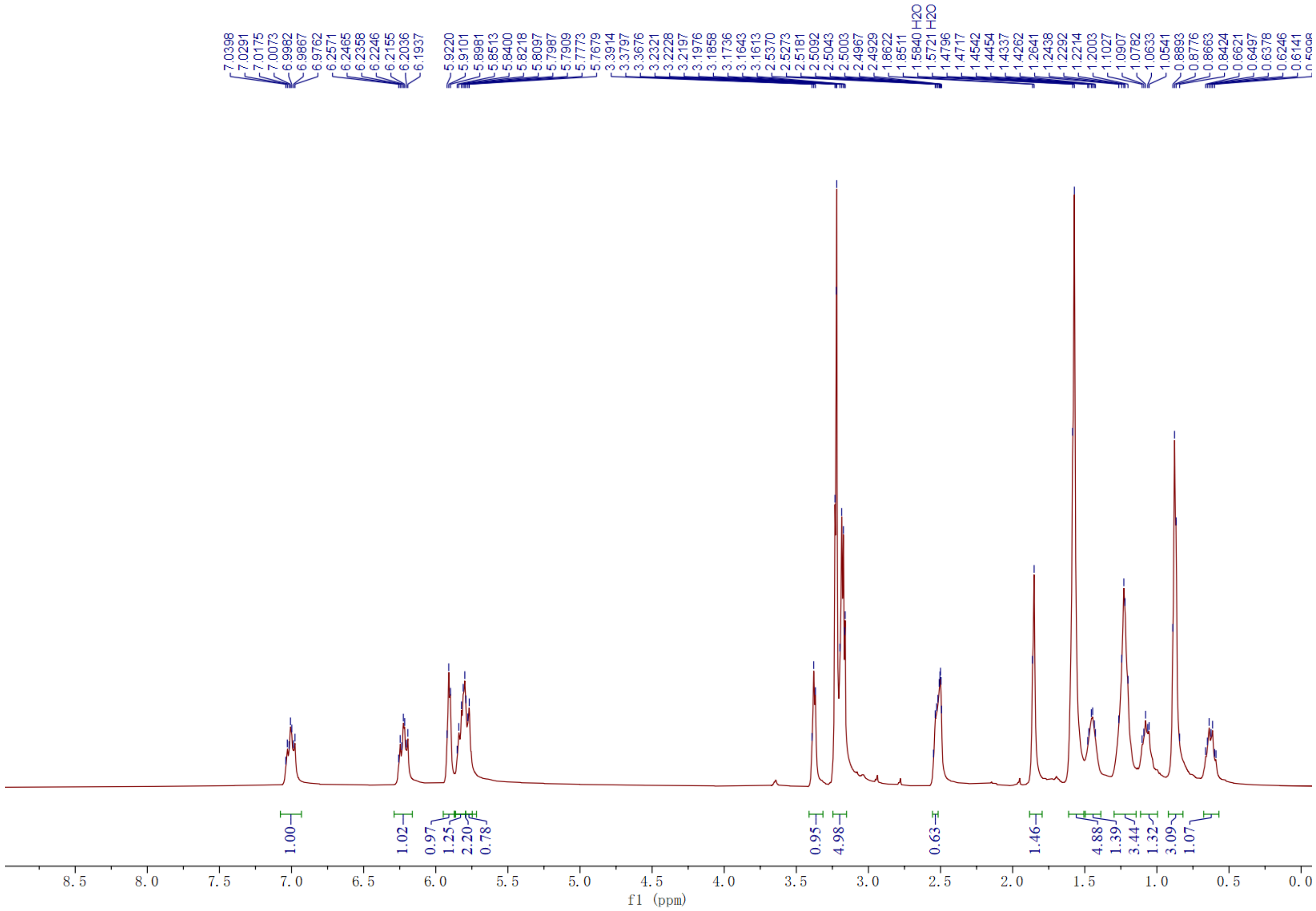

**Figure S45**. $^{13}$C NMR (125 MHz, DMSO-$d_6$) and DEPT spectra of compound **7**;

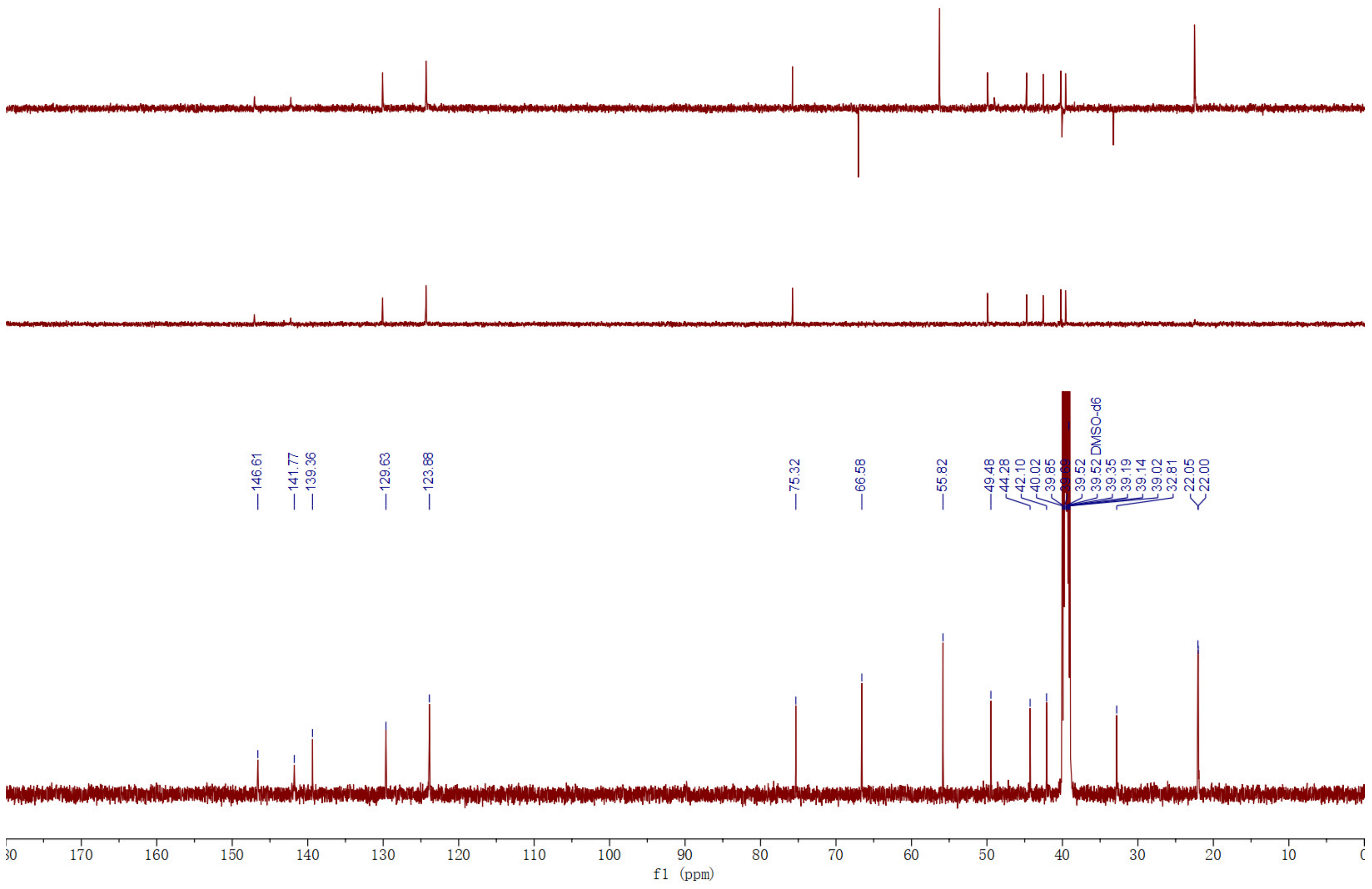

**Figure S46**. COSY spectrum of compound **7**;

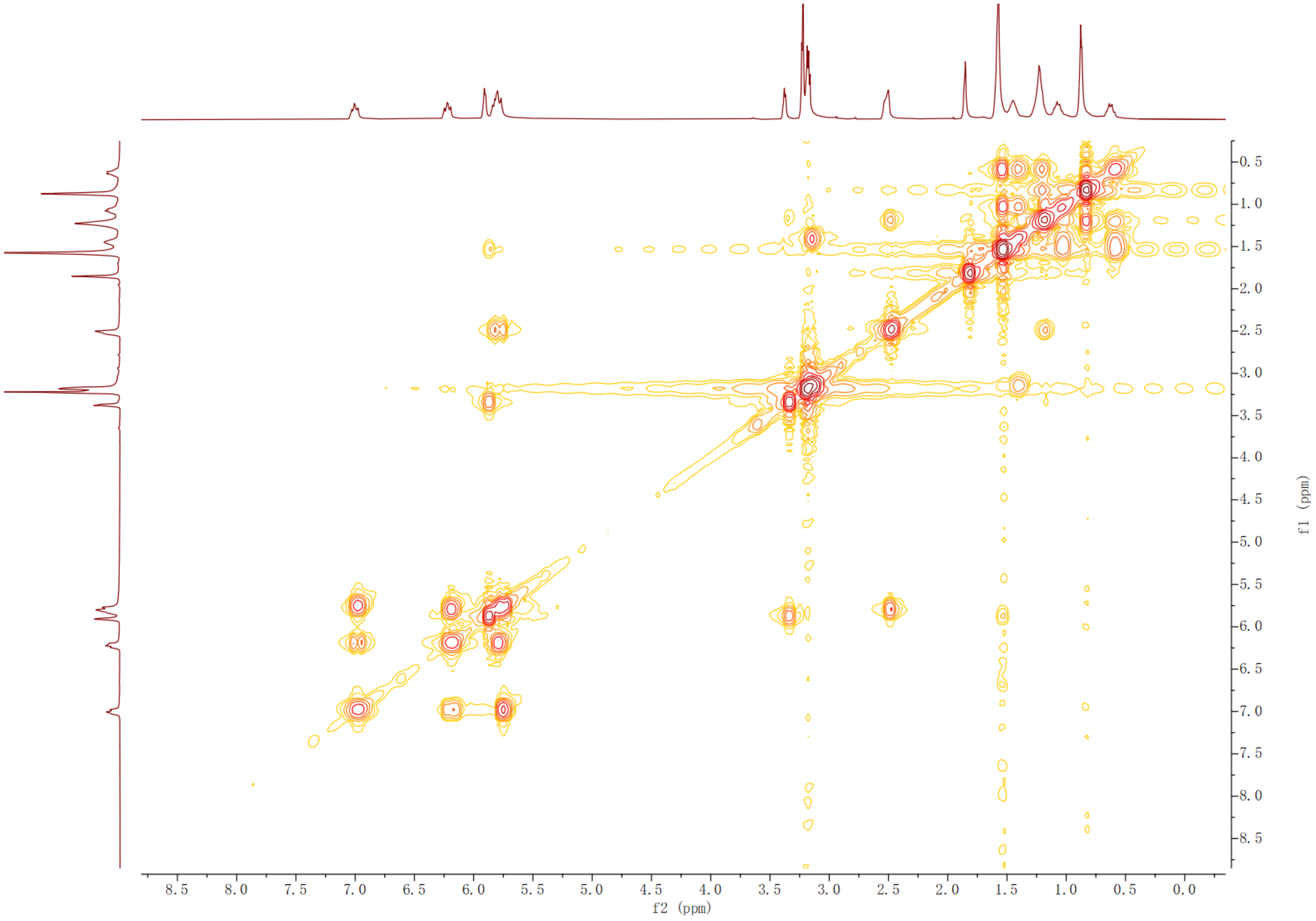

**Figure S47.** HSQC spectrum of compound **7**;

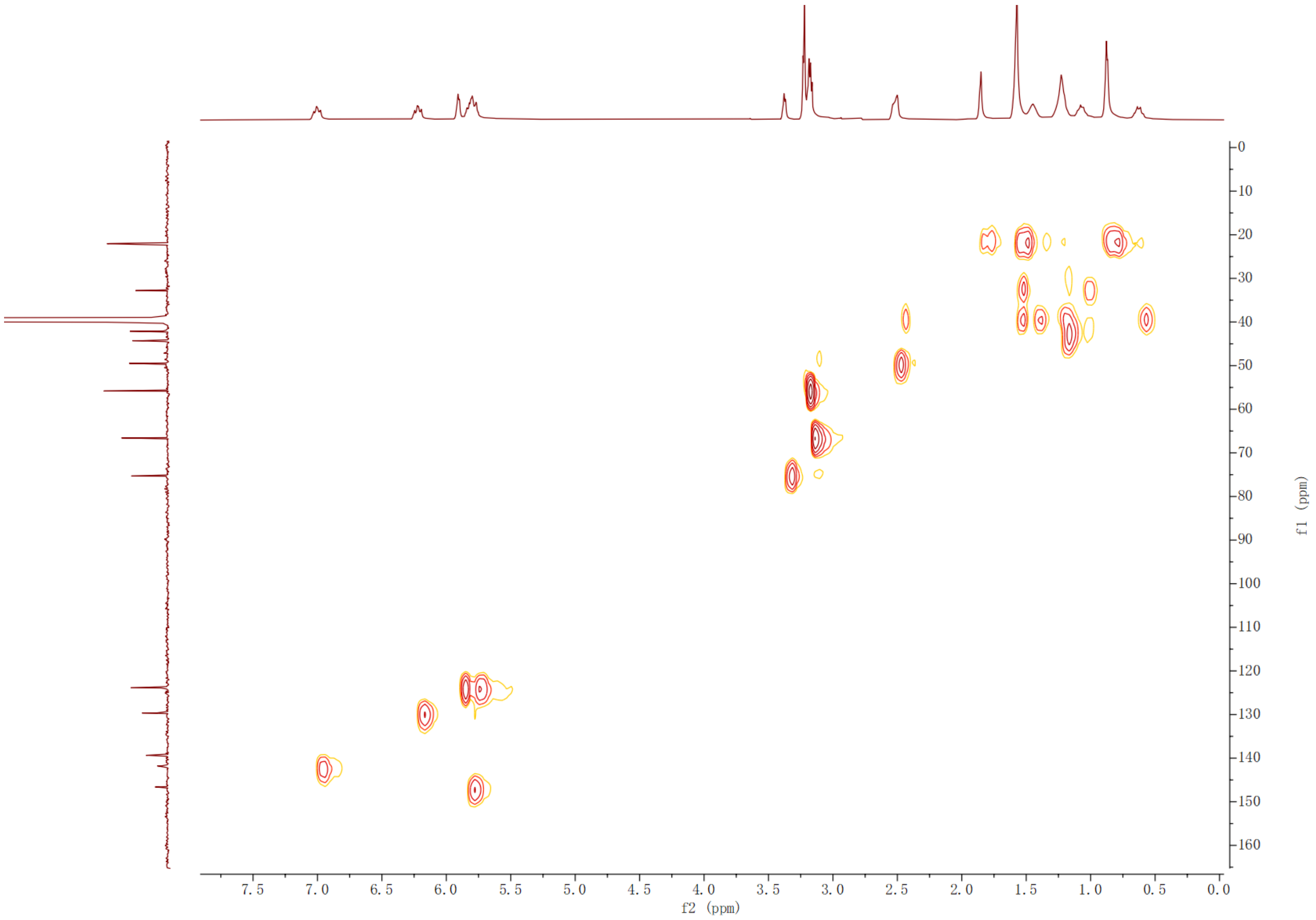

**Figure S48**. HMBC spectrum of compound **7**;

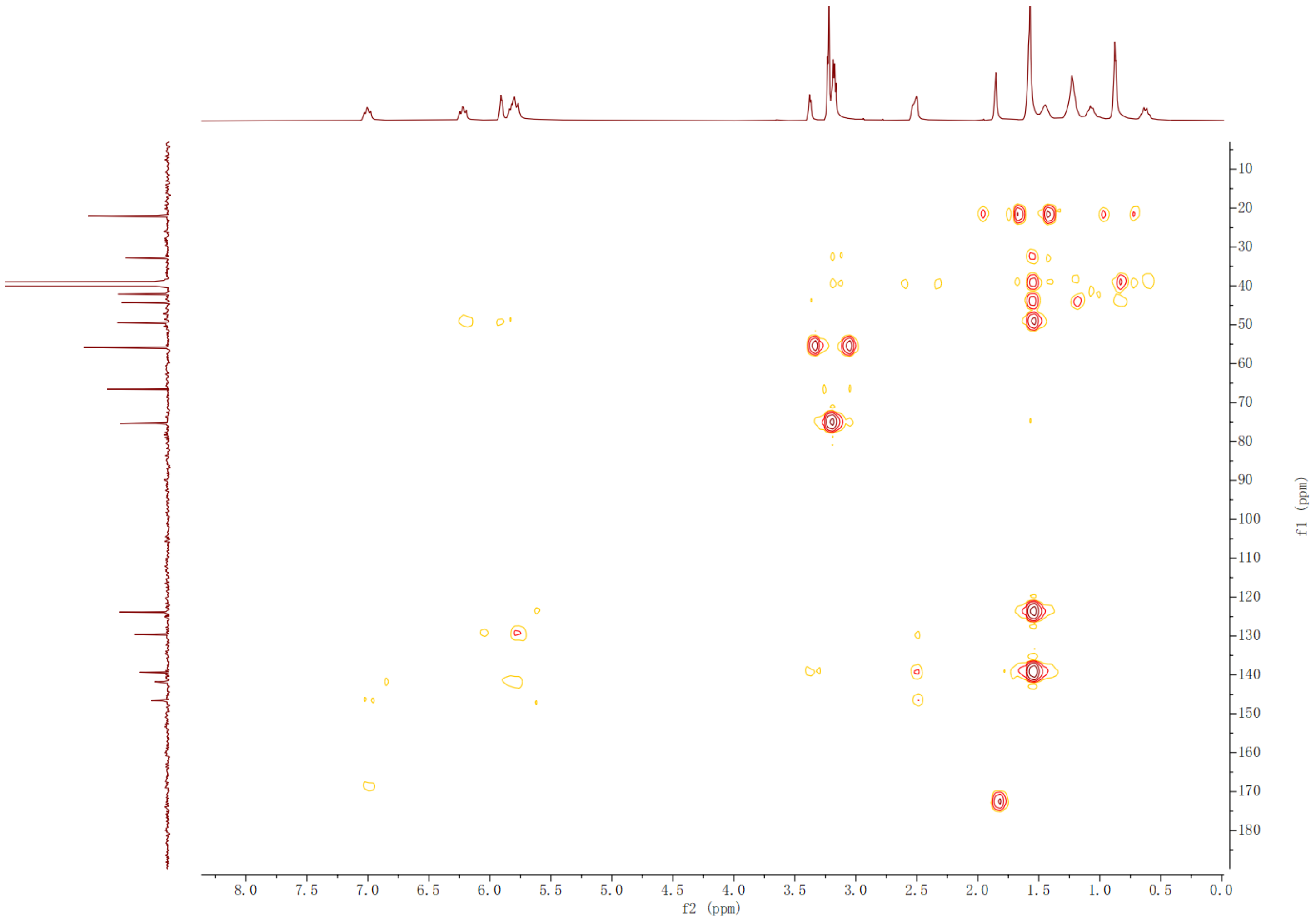

**Figure S49**. NOESY spectrum of compound **7**;

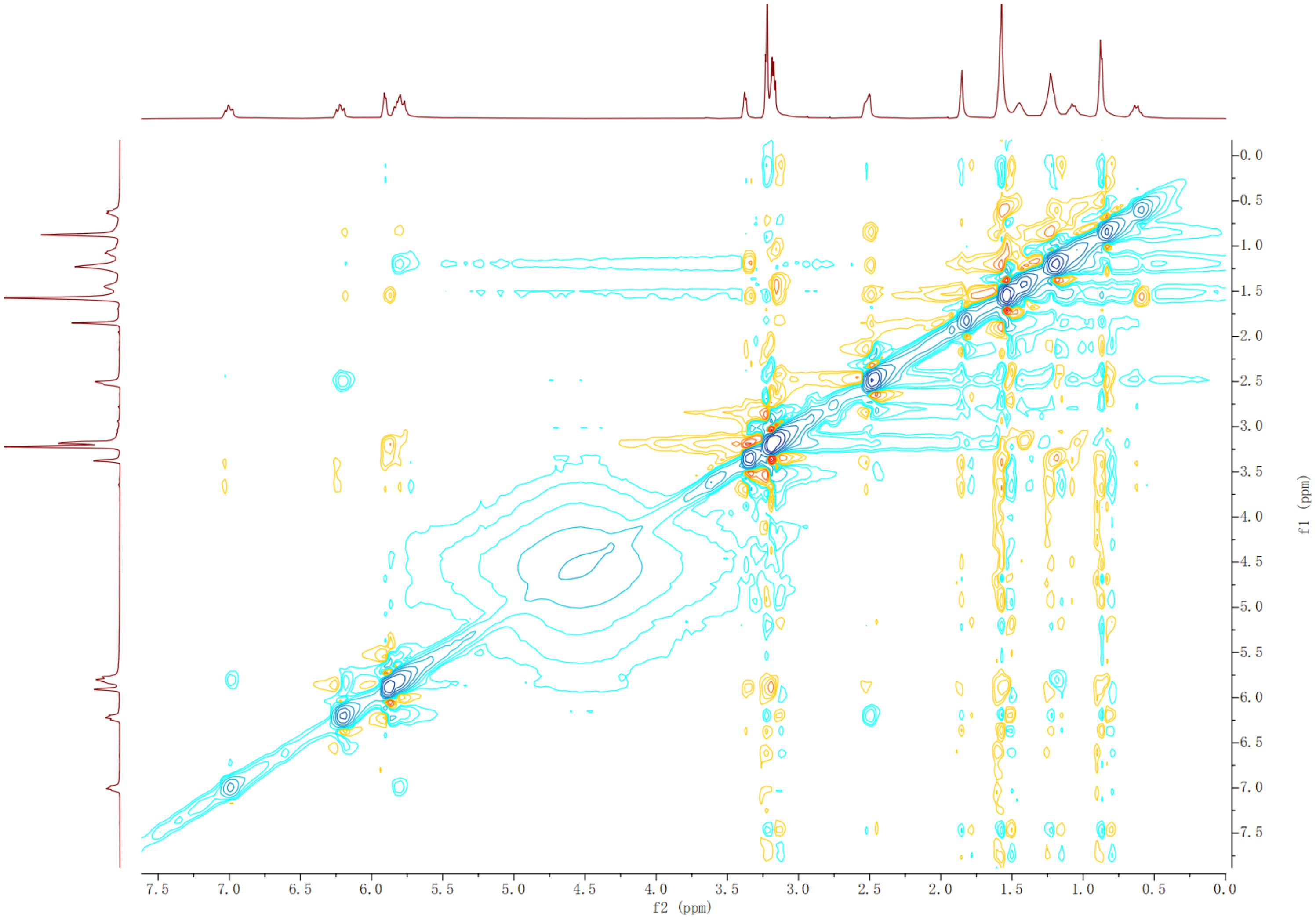

**Figure S50**. HRESI mass spectrum of compound **8**;

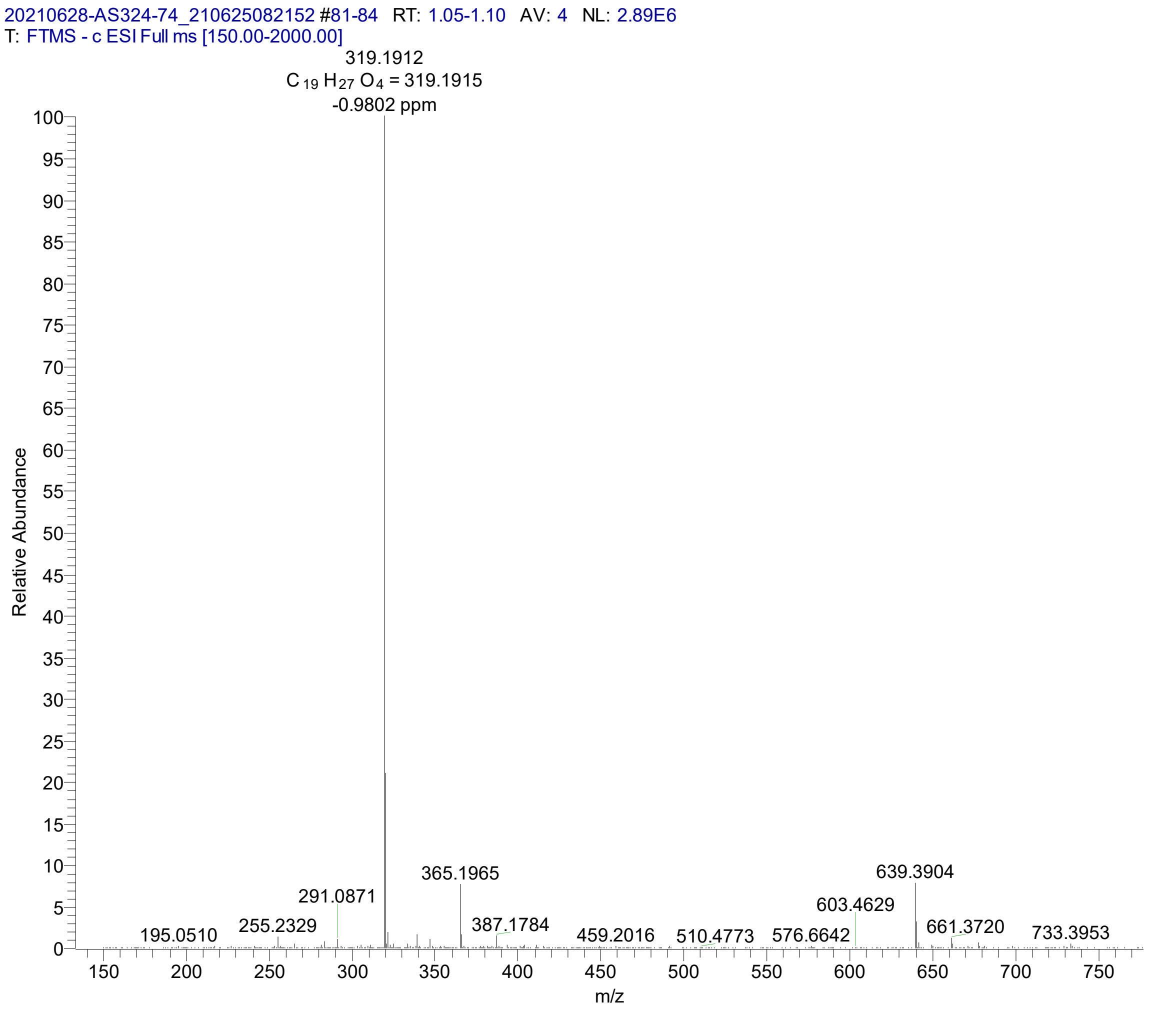

**Figure S51**. $^{1}$H NMR (500 MHz, DMSO-$d_6$) spectrum of compound **8**;

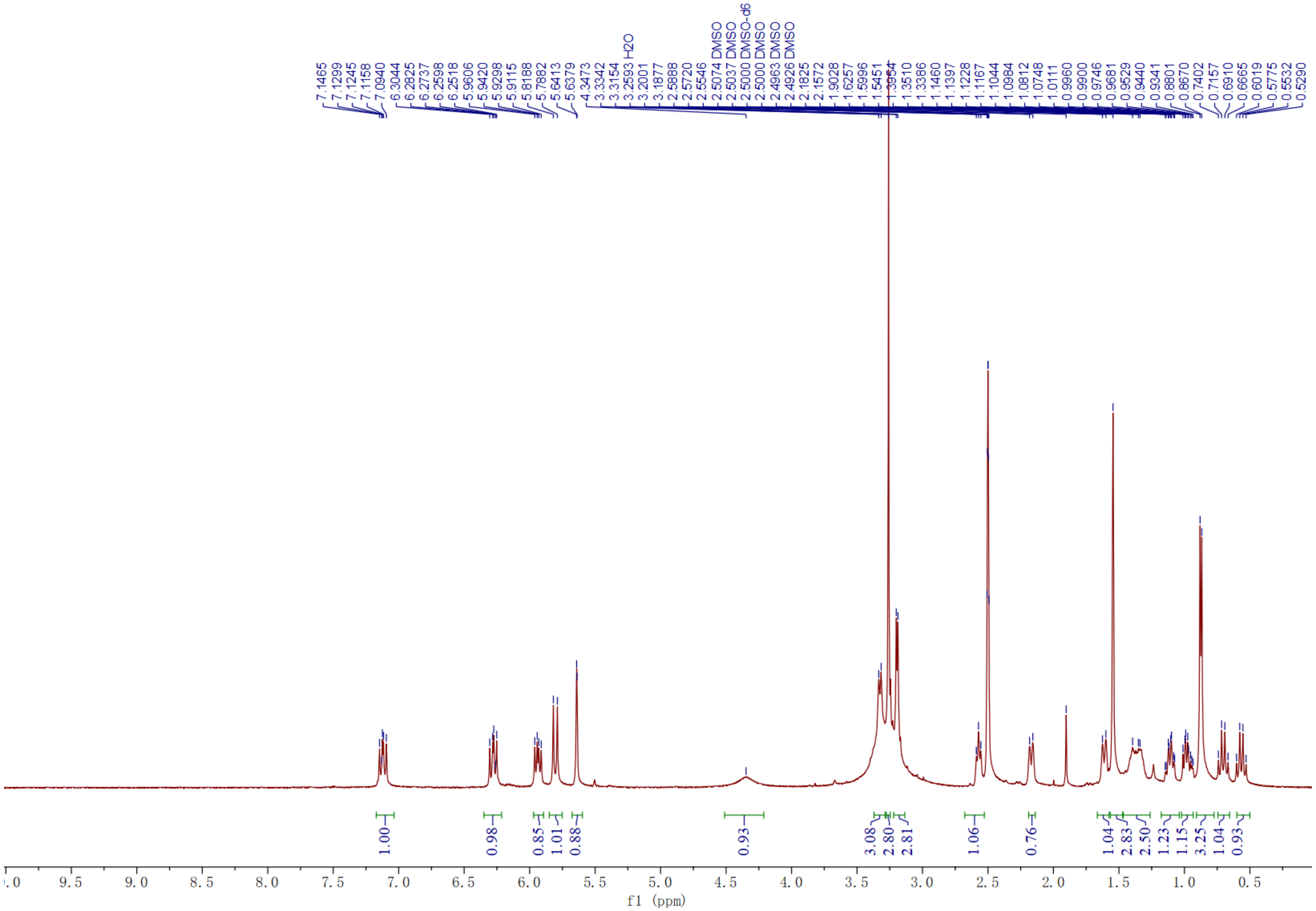

**Figure S52**. $^{13}$C NMR (125 MHz, DMSO-$d_6$) and DEPT spectra of compound **8**;

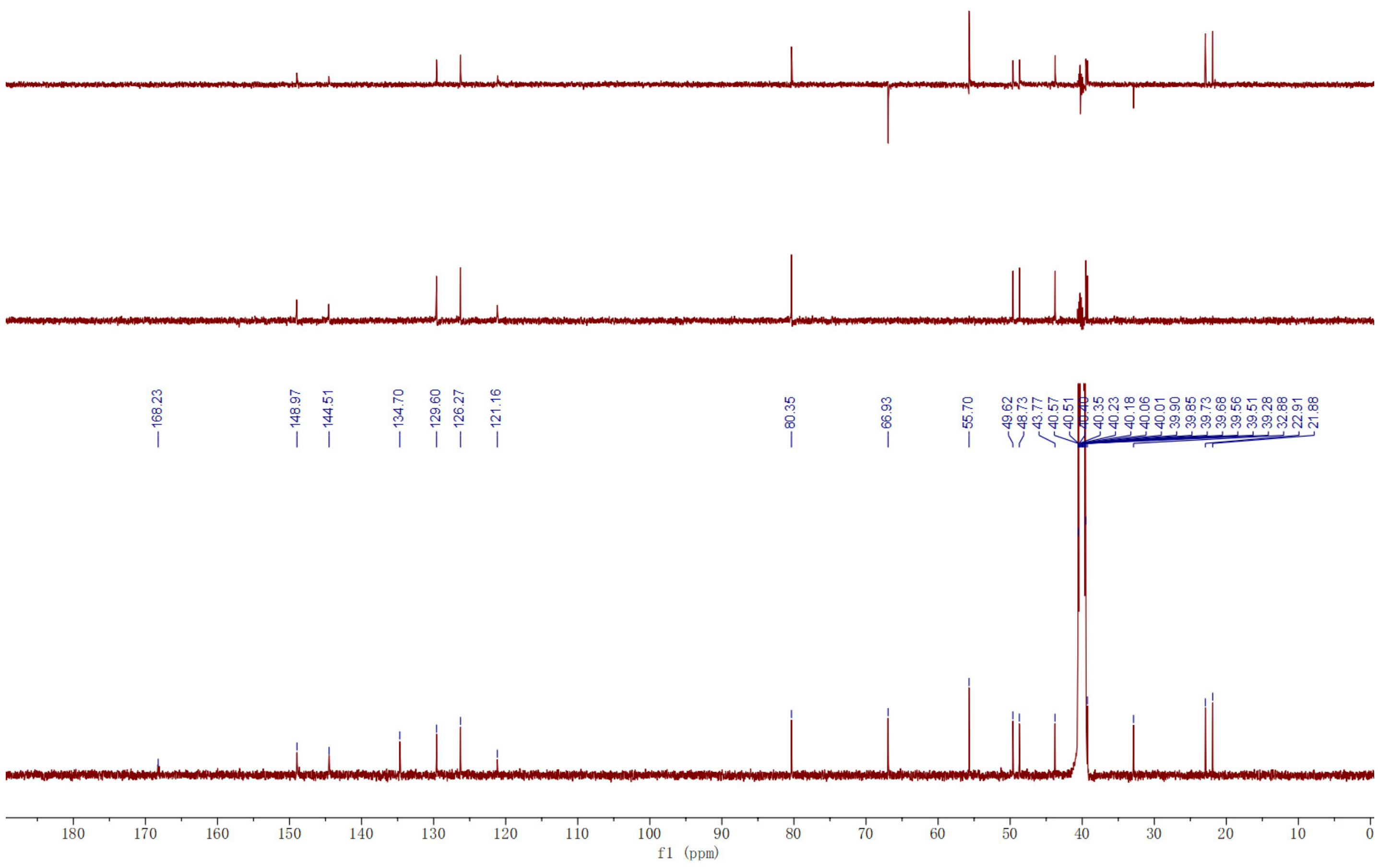

**Figure S53**. COSY spectrum of compound **8**;

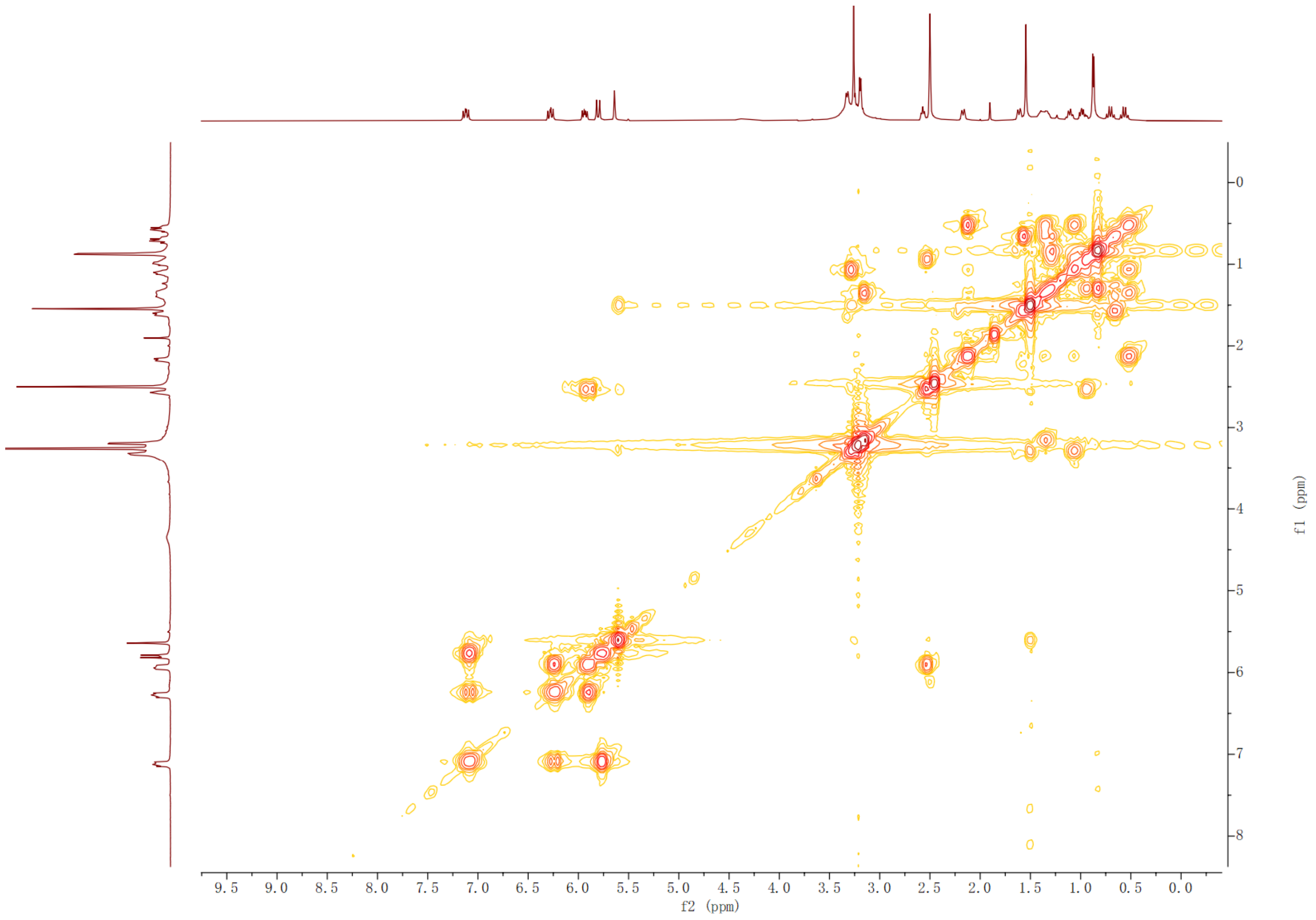

**Figure S54.** HSQC spectrum of compound **8**;

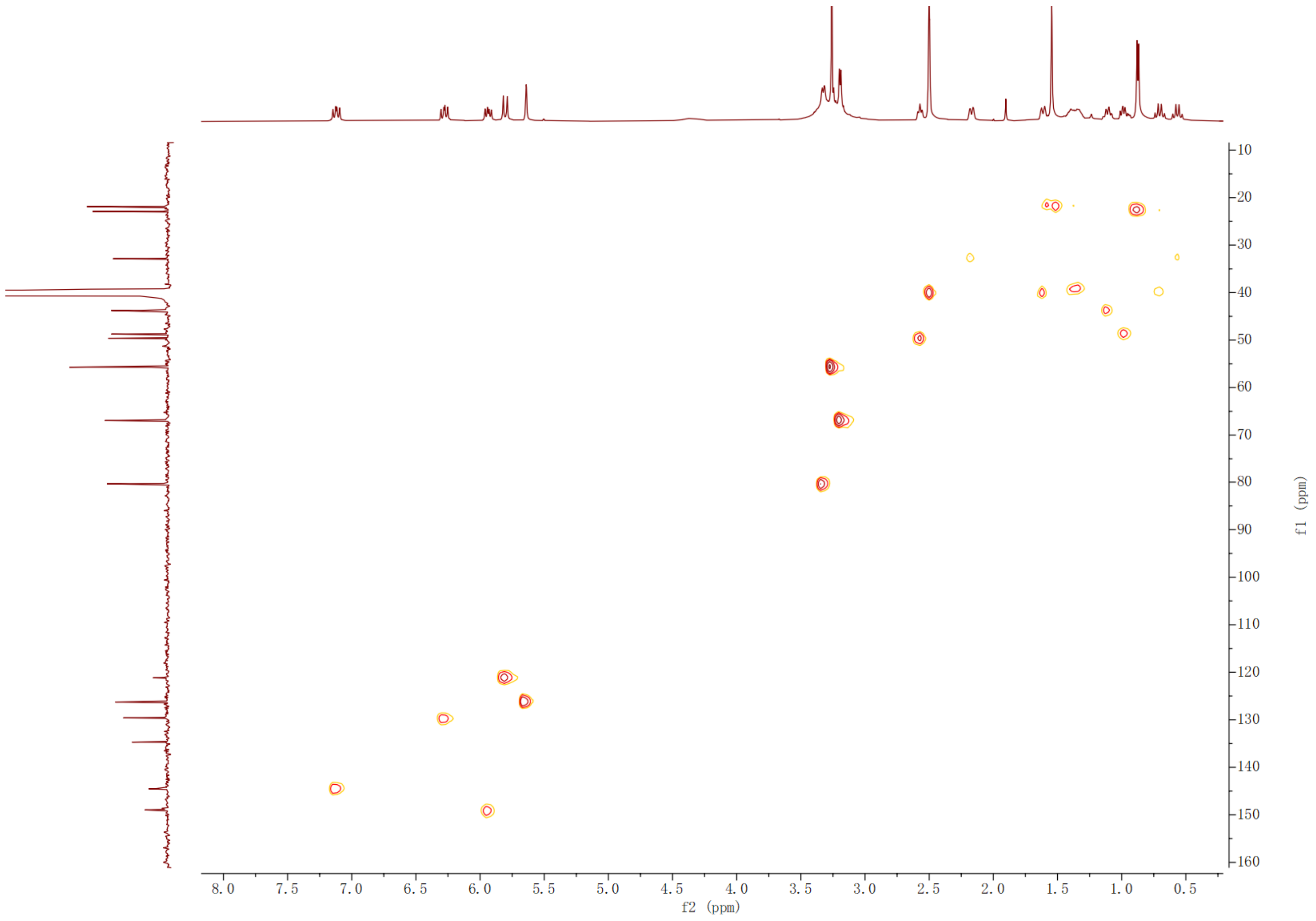

**Figure S55**. HMBC spectrum of compound **8**;

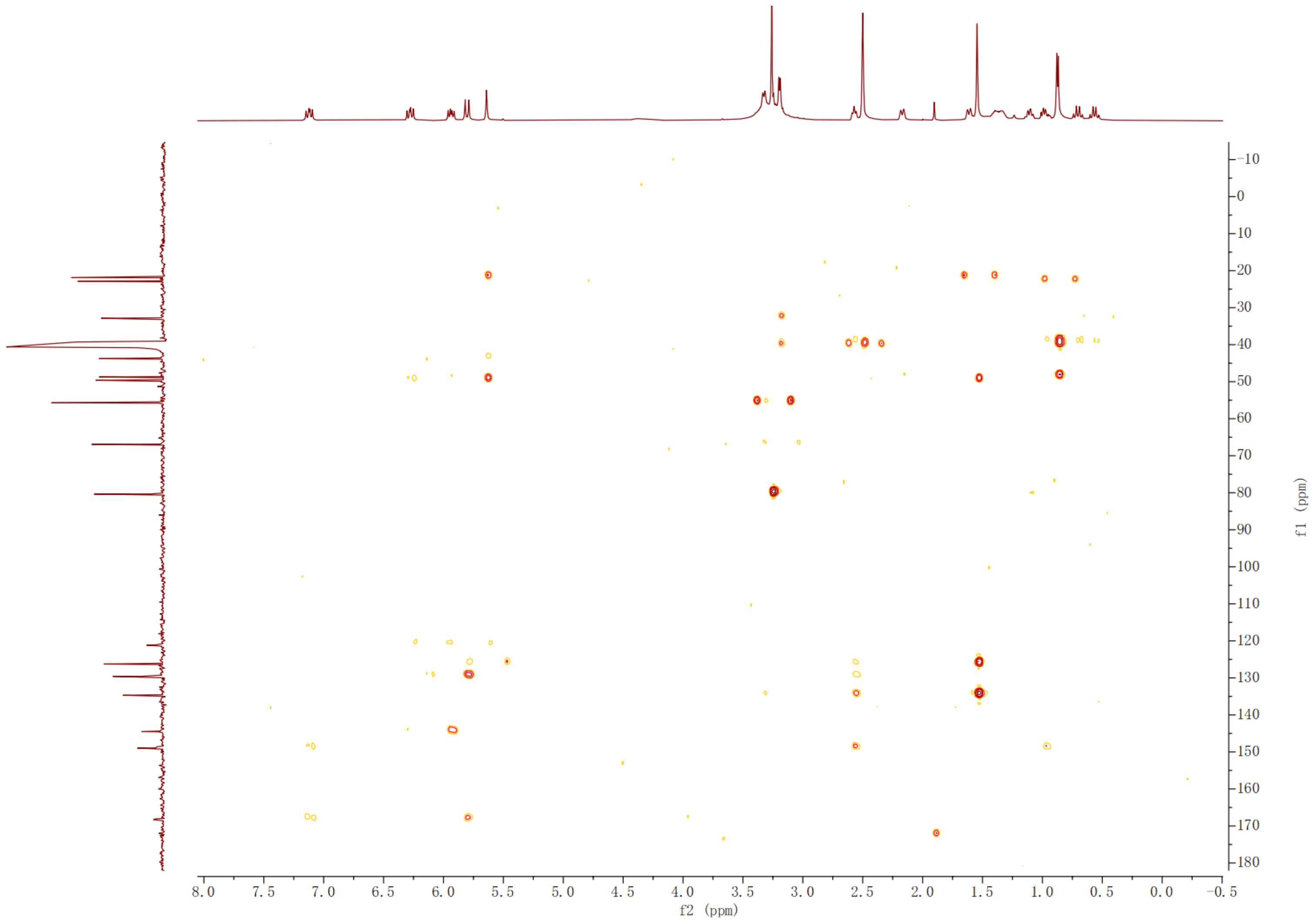

**Figure S56**. NOESY spectrum of compound **8**;

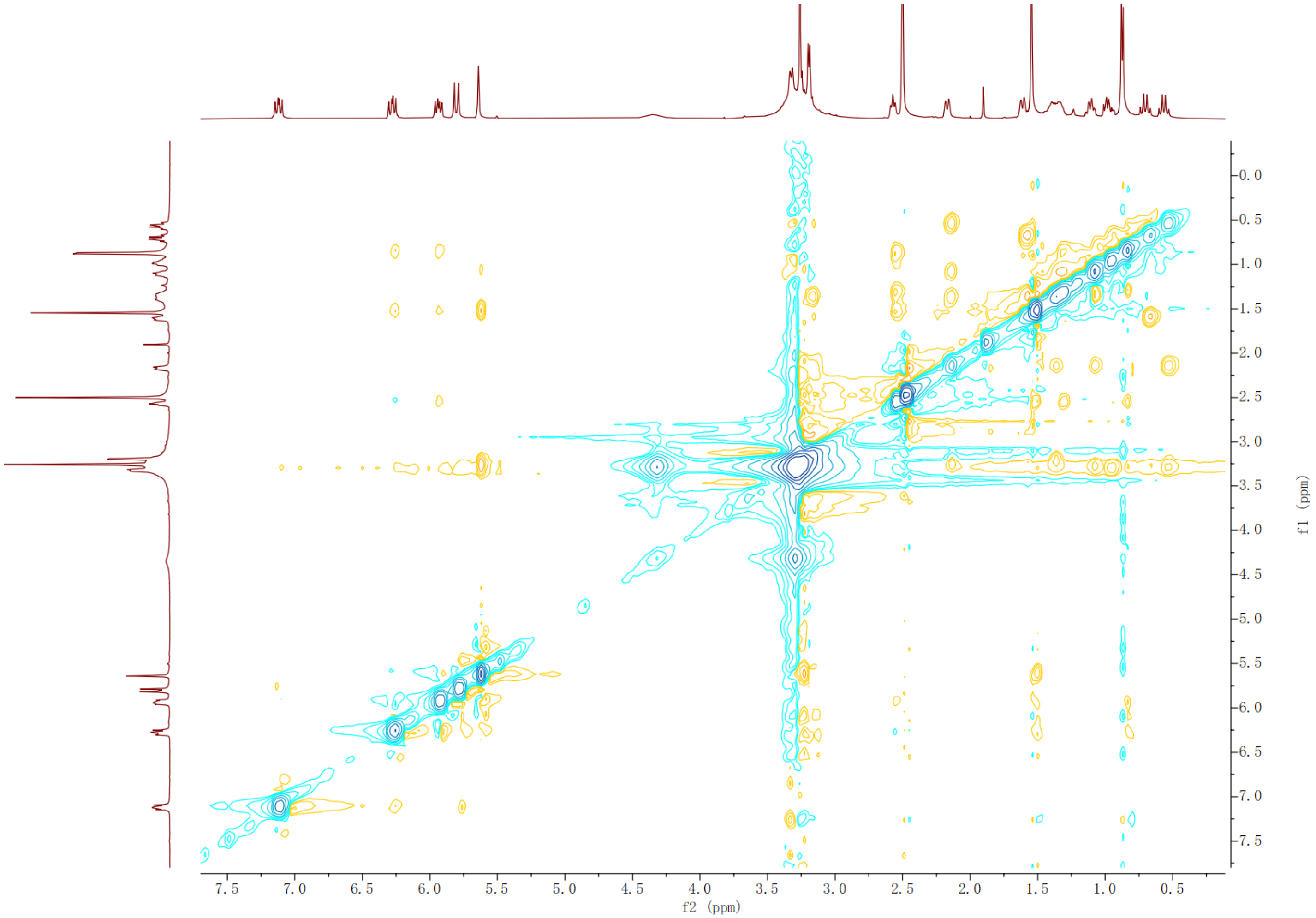

**Figure S57**. Structures and populations of conformers obtained by gas phase B3LYP/6-31G(d) reoptimization of the initial MMFF conformers of (6*R*, 7*R*, 8*R*, 10*S*, 14*S*, and 15*R*)-**4** above 2% population;

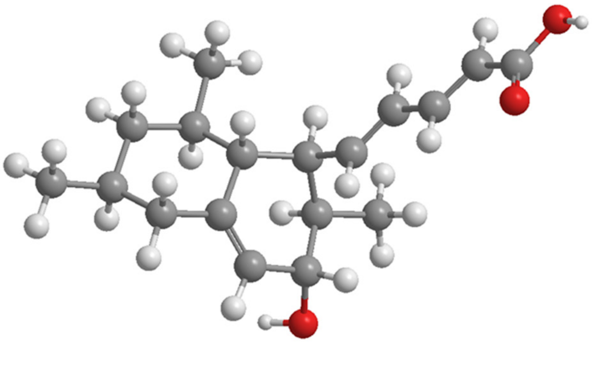

49.04%

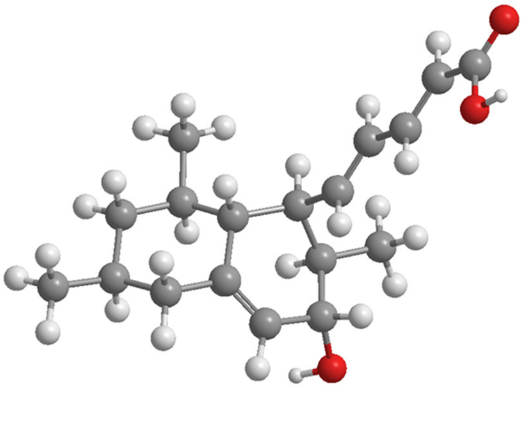

48.96%

**Figure S58**. Structures and populations of conformers obtained by gas phase B3LYP/6-31G(d) reoptimization of the initial MMFF conformers of (6*R*, 7*R*, 8*R*, 10*R*, 12*S*, and 13*S*)-**5** above 2% population;

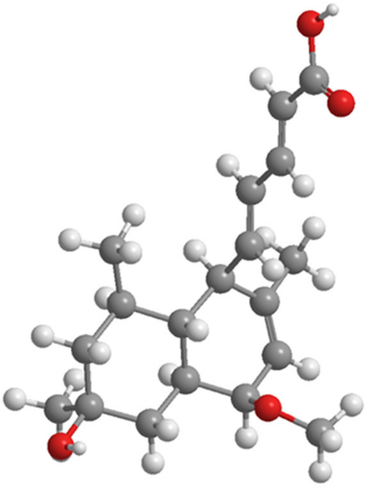

49.03%

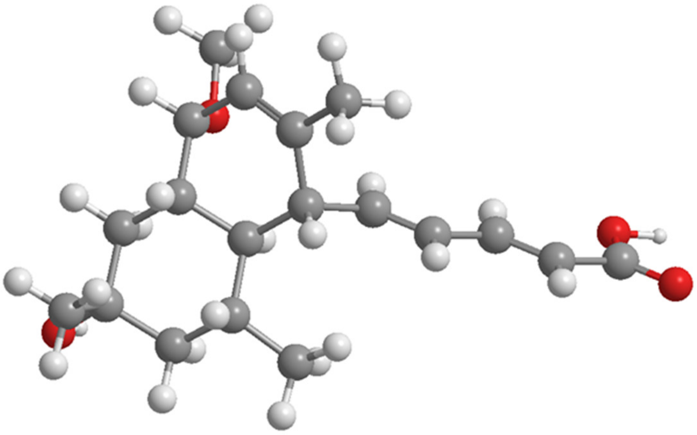

48.96%

**Figure S59**. Experimental and calculated ECD at different levels of compound **4** and **5**;

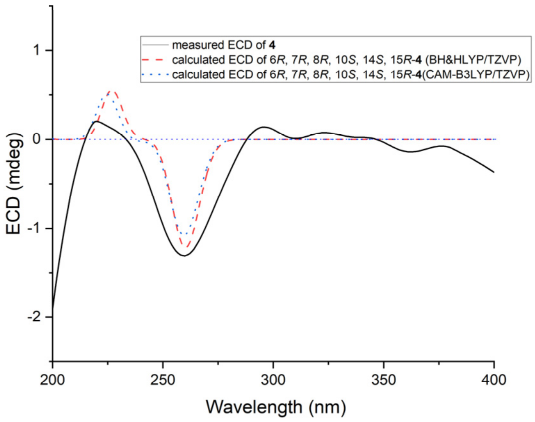


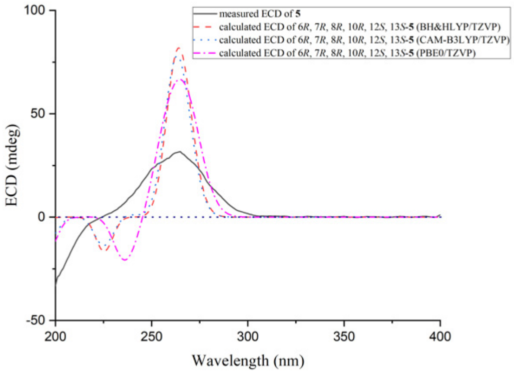

**Table S1**. Cartesian coordinates for the low-energy reoptimized MMFF conformers of **4** calculated at B3LYP/6-31G(d) level of theory in vacuo;

| Compound **4** Conformer A | | Standard Orientation (Ångstroms) | | |
|---|---|---|---|---|
| | Type | X | Y | Z |
| 1 | C | -3.430922 | -1.837529 | 0.712851 |
| 2 | C | -4.059837 | -1.357744 | -0.606239 |
| 3 | C | -2.952493 | -0.941961 | -1.601788 |
| 4 | C | -2.020619 | 0.05162 | -0.955533 |
| 5 | C | -1.347473 | -0.451088 | 0.311031 |
| 6 | C | -2.439744 | -0.842037 | 1.356015 |
| 7 | C | -1.866771 | 1.303371 | -1.416432 |
| 8 | C | -1.069721 | 2.383223 | -0.73 |
| 9 | C | -0.739164 | 2.007703 | 0.725231 |
| 10 | C | -0.248961 | 0.533243 | 0.814727 |
| 11 | C | 1.029539 | 0.293616 | 0.053187 |
| 12 | C | 2.213847 | -0.033953 | 0.60963 |
| 13 | C | 3.410356 | -0.289913 | -0.165473 |
| 14 | C | 4.610791 | -0.617972 | 0.360385 |
| 15 | C | 5.773553 | -0.863044 | -0.505587 |
| 16 | O | 6.883488 | -1.176705 | 0.223041 |
| 17 | O | 5.799353 | -0.807608 | -1.723458 |
| 18 | C | -1.860688 | -1.45082 | 2.643084 |
| 19 | C | -4.993126 | -2.415714 | -1.207568 |
| 20 | C | 0.229723 | 3.001208 | 1.37557 |
| 21 | O | -1.764761 | 3.641097 | -0.805019 |
| 22 | H | -0.841116 | -1.397317 | 0.055109 |
| 23 | H | -0.040815 | 0.325403 | 1.870414 |
| 24 | H | -4.227312 | -2.066325 | 1.434845 |
| 25 | H | -2.90193 | -2.787401 | 0.529343 |
| 26 | H | -4.656574 | -0.457787 | -0.389301 |
| 27 | H | -2.393826 | -1.843969 | -1.900694 |
| 28 | H | -3.397438 | -0.521658 | -2.512304 |
| 29 | H | -2.997709 | 0.068116 | 1.624841 |
| 30 | H | -2.364009 | 1.608164 | -2.338174 |
| 31 | H | -0.139485 | 2.583923 | -1.279196 |
| 32 | H | -1.690382 | 2.049466 | 1.27908 |
| 33 | H | 0.97333 | 0.363427 | -1.034784 |
| 34 | H | 2.294515 | -0.11753 | 1.694588 |
| 35 | H | 3.334591 | -0.212912 | -1.250102 |
| 36 | H | 4.761884 | -0.712071 | 1.432308 |
| 37 | H | 7.605445 | -1.322876 | -0.416178 |
| 38 | H | -1.220087 | -2.313845 | 2.417072 |
| 39 | H | -1.267493 | -0.734501 | 3.221202 |
| 40 | H | -2.669538 | -1.801253 | 3.295946 |
| 41 | H | -4.442906 | -3.335115 | -1.448514 |
| 42 | H | -5.460199 | -2.054297 | -2.132199 |
| 43 | H | -5.796208 | -2.68065 | -0.508534 |

| | | | | |
|---|---|---|---|---|
| 44 | H | -0.165301 | 4.019704 | 1.30783 |
| 45 | H | 1.209711 | 2.984947 | 0.884052 |
| 46 | H | 0.38448 | 2.762651 | 2.43551 |
| 47 | H | -2.666003 | 3.50618 | -0.467216 |

B3LYP Energy = -926.904287.

| Compound **4** Conformer **B** | | Standard Orientation (Ångstroms) | | |
|---|---|---|---|---|
| | Type | X | Y | Z |
| 1 | C | -3.438337 | -1.817075 | 0.713268 |
| 2 | C | -4.049904 | -1.347314 | -0.617575 |
| 3 | C | -2.929109 | -0.948922 | -1.605235 |
| 4 | C | -1.99836 | 0.045779 | -0.959102 |
| 5 | C | -1.342052 | -0.447745 | 0.319849 |
| 6 | C | -2.448254 | -0.820701 | 1.356758 |
| 7 | C | -1.832898 | 1.29224 | -1.430364 |
| 8 | C | -1.037528 | 2.374594 | -0.745623 |
| 9 | C | -0.725583 | 2.011468 | 0.71691 |
| 10 | C | -0.243269 | 0.535565 | 0.824689 |
| 11 | C | 1.041059 | 0.284169 | 0.076582 |
| 12 | C | 2.218638 | -0.045285 | 0.645434 |
| 13 | C | 3.42329 | -0.310101 | -0.116254 |
| 14 | C | 4.612486 | -0.640691 | 0.435162 |
| 15 | C | 5.836165 | -0.91104 | -0.32913 |
| 16 | O | 5.685405 | -0.799254 | -1.681453 |
| 17 | O | 6.907297 | -1.211453 | 0.170876 |
| 18 | C | -1.887311 | -1.418779 | 2.65675 |
| 19 | C | -4.983199 | -2.405852 | -1.217891 |
| 20 | C | 0.240475 | 3.006348 | 1.36932 |
| 21 | O | -1.723902 | 3.635625 | -0.841732 |
| 22 | H | -0.838781 | -1.399761 | 0.079871 |
| 23 | H | -0.045418 | 0.336155 | 1.883958 |
| 24 | H | -4.24382 | -2.033645 | 1.428869 |
| 25 | H | -2.913118 | -2.771895 | 0.545272 |
| 26 | H | -4.643285 | -0.44146 | -0.416691 |
| 27 | H | -2.372822 | -1.857565 | -1.888117 |
| 28 | H | -3.36141 | -0.535695 | -2.52506 |
| 29 | H | -3.003598 | 0.095665 | 1.609229 |
| 30 | H | -2.319137 | 1.590898 | -2.359972 |
| 31 | H | -0.099705 | 2.564221 | -1.285888 |
| 32 | H | -1.682865 | 2.062812 | 1.259375 |
| 33 | H | 0.994664 | 0.347667 | -1.012302 |
| 34 | H | 2.289702 | -0.123274 | 1.731433 |
| 35 | H | 3.3511 | -0.234671 | -1.200145 |
| 36 | H | 4.734617 | -0.728053 | 1.511276 |
| 37 | H | 6.55594 | -0.999105 | -2.07135 |
| 38 | H | -2.705457 | -1.756577 | 3.304652 |
| 39 | H | -1.250126 | -2.288542 | 2.447479 |
| 40 | H | -1.29565 | -0.699975 | 3.233329 |

| | | | | |
|---|---|---|---|---|
| 41 | H | -5.438671 | -2.051215 | -2.150903 |
| 42 | H | -5.794929 | -2.658962 | -0.524516 |
| 43 | H | -4.43598 | -3.33088 | -1.44368 |
| 44 | H | 1.22613 | 2.980898 | 0.889657 |
| 45 | H | 0.381668 | 2.776803 | 2.4331 |
| 46 | H | -0.14868 | 4.026082 | 1.287698 |
| 47 | H | -2.62863 | 3.510785 | -0.5094 |

B3LYP Energy = -926.903351

**Table S2**. Cartesian coordinates for the low-energy reoptimized MMFF conformers of **5** calculated at B3LYP/6-31G(d) level of theory in vacuo.

| Compound **5** Conformer A | | Standard Orientation (Ångstroms) | | |
|---|---|---|---|---|
| | Type | X | Y | Z |
| 1 | C | -2.525044 | -2.343825 | -0.304048 |
| 2 | C | -3.839207 | -1.541247 | -0.291768 |
| 3 | C | -3.536702 | -0.074297 | -0.645076 |
| 4 | C | -2.435889 | 0.542965 | 0.231887 |
| 5 | C | -1.124398 | -0.269974 | 0.1488 |
| 6 | C | -1.384496 | -1.747887 | 0.54896 |
| 7 | C | -2.18696 | 2.018805 | -0.106032 |
| 8 | C | -1.164282 | 2.588991 | 0.846472 |
| 9 | C | -0.181039 | 1.8455 | 1.37401 |
| 10 | C | 0.041965 | 0.382508 | 0.971719 |
| 11 | C | -0.147279 | -2.653031 | 0.434109 |
| 12 | O | -4.753537 | -2.095299 | -1.261258 |
| 13 | C | 1.338448 | 0.321689 | 0.19481 |
| 14 | C | 2.513397 | -0.170044 | 0.637061 |
| 15 | C | 3.722494 | -0.164458 | -0.159089 |
| 16 | C | 4.915053 | -0.649168 | 0.251946 |
| 17 | C | 6.09368 | -0.600888 | -0.625384 |
| 18 | O | 7.190265 | -1.142664 | -0.020964 |
| 19 | O | 6.142298 | -0.146814 | -1.756117 |
| 20 | C | 0.772671 | 2.402713 | 2.402085 |
| 21 | O | -1.790601 | 2.097956 | -1.485266 |
| 22 | C | -4.58891 | -1.682629 | 1.036614 |
| 23 | C | -1.702285 | 3.418328 | -1.991239 |
| 24 | H | -0.824276 | -0.268134 | -0.909049 |
| 25 | H | 0.17723 | -0.182061 | 1.90721 |
| 26 | H | -2.781026 | 0.540332 | 1.27591 |
| 27 | H | -2.173718 | -2.407282 | -1.346834 |
| 28 | H | -2.732083 | -3.373574 | 0.014679 |
| 29 | H | -4.460179 | 0.51509 | -0.567161 |
| 30 | H | -3.216295 | -0.022058 | -1.694823 |
| 31 | H | -1.702732 | -1.752716 | 1.603967 |

| | | | | |
|---|---|---|---|---|
| 32 | H | -3.132976 | 2.576242 | 0.004996 |
| 33 | H | -1.270904 | 3.628594 | 1.154595 |
| 34 | H | -0.412761 | -3.689292 | 0.677222 |
| 35 | H | 0.657964 | -2.352733 | 1.110754 |
| 36 | H | 0.257926 | -2.642961 | -0.585918 |
| 37 | H | -4.360453 | -2.003648 | -2.144874 |
| 38 | H | 1.292605 | 0.755932 | -0.805681 |
| 39 | H | 2.576457 | -0.603734 | 1.63633 |
| 40 | H | 3.664518 | 0.261476 | -1.160724 |
| 41 | H | 5.047499 | -1.088627 | 1.236873 |
| 42 | H | 7.923886 | -1.075199 | -0.66004 |
| 43 | H | 0.713732 | 1.828149 | 3.33774 |
| 44 | H | 0.541635 | 3.447706 | 2.632396 |
| 45 | H | 1.814529 | 2.348155 | 2.064071 |
| 46 | H | -3.998391 | -1.320355 | 1.883709 |
| 47 | H | -4.834425 | -2.735359 | 1.214256 |
| 48 | H | -5.526255 | -1.116653 | 1.000671 |
| 49 | H | -1.482349 | 3.333268 | -3.05868 |
| 50 | H | -2.654275 | 3.958452 | -1.863068 |
| 51 | H | -0.899597 | 3.991253 | -1.5047 |

B3LYP Energy = -1041.394654.

| Compound **5** Conformer B | | Standard Orientation (Ångstroms) | | |
|---|---|---|---|---|
| | Type | X | Y | Z |
| 1 | C | -2.523748 | -2.337095 | -0.308258 |
| 2 | C | -3.834307 | -1.528663 | -0.303353 |
| 3 | C | -3.523085 | -0.063135 | -0.655028 |
| 4 | C | -2.424419 | 0.549236 | 0.228095 |
| 5 | C | -1.116178 | -0.269638 | 0.152862 |
| 6 | C | -1.385492 | -1.746442 | 0.551445 |
| 7 | C | -2.167136 | 2.023966 | -0.108441 |
| 8 | C | -1.146482 | 2.589802 | 0.848741 |
| 9 | C | -0.169748 | 1.841941 | 1.382245 |
| 10 | C | 0.048099 | 0.377429 | 0.982909 |
| 11 | C | -0.151959 | -2.657466 | 0.444179 |
| 12 | O | -4.745656 | -2.078465 | -1.278051 |
| 13 | C | 1.349663 | 0.307974 | 0.21499 |
| 14 | C | 2.518441 | -0.19019 | 0.665516 |
| 15 | C | 3.734675 | -0.195252 | -0.121911 |
| 16 | C | 4.916597 | -0.689647 | 0.310536 |
| 17 | C | 6.153054 | -0.697785 | -0.48001 |
| 18 | O | 6.022991 | -0.152686 | -1.725056 |
| 19 | O | 7.217833 | -1.144477 | -0.087094 |
| 20 | C | 0.7804 | 2.395154 | 2.415754 |
| 21 | O | -1.7639 | 2.101972 | -1.486061 |
| 22 | C | -4.59225 | -1.666566 | 1.020713 |
| 23 | C | -1.670529 | 3.422161 | -1.991315 |
| 24 | H | -0.809652 | -0.269221 | -0.903211 |

| | | | | |
|---|---|---|---|---|
| 25 | H | 0.173634 | -0.186808 | 1.91993 |
| 26 | H | -2.775507 | 0.548375 | 1.27012 |
| 27 | H | -2.166707 | -2.401861 | -1.34905 |
| 28 | H | -2.7372 | -3.365959 | 0.009011 |
| 29 | H | -4.444236 | 0.530545 | -0.582533 |
| 30 | H | -3.196387 | -0.012615 | -1.702941 |
| 31 | H | -1.710157 | -1.749728 | 1.60447 |
| 32 | H | -3.111171 | 2.585576 | -0.002022 |
| 33 | H | -1.249676 | 3.630135 | 1.155587 |
| 34 | H | -0.423953 | -3.692505 | 0.685171 |
| 35 | H | 0.650385 | -2.361462 | 1.126061 |
| 36 | H | 0.259755 | -2.649051 | -0.573271 |
| 37 | H | -4.346783 | -1.989506 | -2.159335 |
| 38 | H | 1.312441 | 0.741083 | -0.786467 |
| 39 | H | 2.572758 | -0.622517 | 1.665844 |
| 40 | H | 3.678688 | 0.228979 | -1.123175 |
| 41 | H | 5.022282 | -1.12307 | 1.301297 |
| 42 | H | 6.90018 | -0.217086 | -2.144818 |
| 43 | H | 1.824341 | 2.332214 | 2.085783 |
| 44 | H | 0.710504 | 1.823427 | 3.352371 |
| 45 | H | 0.555069 | 3.442305 | 2.641862 |
| 46 | H | -4.004951 | -1.307155 | 1.871256 |
| 47 | H | -4.843711 | -2.718128 | 1.196868 |
| 48 | H | -5.526735 | -1.096246 | 0.979444 |
| 49 | H | -2.621554 | 3.9647 | -1.866374 |
| 50 | H | -0.868205 | 3.993105 | -1.501732 |
| 51 | H | -1.447181 | 3.336998 | -3.058091 |

B3LYP Energy = -1041.393827.
